# Gender-stratified analysis of sepsis mortality in cancer: a 45-year population-based cohort study

**DOI:** 10.1093/jncics/pkaf109

**Published:** 2025-11-10

**Authors:** Yadong Guo, Ziyou Lin, Wentao Zhang, Haotian Chen, Yongqiang Liu, Ji Liu, Junfeng Zhang, Aihong Zhang, Shiyu Mao, Xudong Yao

**Affiliations:** Department of Urology, Shanghai Tenth People’s Hospital, School of Medicine, Tongji University, Shanghai, PR China; Urologic Cancer Institute, Tongji University School of Medicine, Shanghai, PR China; Department of Respiratory and Critical Care Medicine, Shanghai Chest Hospital, Shanghai Jiao Tong University School of Medicine, Shanghai, PR China; Department of Urology, Shanghai Tenth People’s Hospital, School of Medicine, Tongji University, Shanghai, PR China; Urologic Cancer Institute, Tongji University School of Medicine, Shanghai, PR China; Department of Urology, Shanghai Tenth People’s Hospital, School of Medicine, Tongji University, Shanghai, PR China; Urologic Cancer Institute, Tongji University School of Medicine, Shanghai, PR China; Department of Urology, Shanghai Tenth People’s Hospital, School of Medicine, Tongji University, Shanghai, PR China; Urologic Cancer Institute, Tongji University School of Medicine, Shanghai, PR China; Department of Urology, Shanghai Tenth People’s Hospital, School of Medicine, Tongji University, Shanghai, PR China; Urologic Cancer Institute, Tongji University School of Medicine, Shanghai, PR China; Department of Urology, Shanghai Tenth People’s Hospital, School of Medicine, Tongji University, Shanghai, PR China; Urologic Cancer Institute, Tongji University School of Medicine, Shanghai, PR China; Department of Colorectal Surgery, Fudan University Shanghai Cancer Center, Shanghai, PR China; Department of Urology, Shanghai Tenth People’s Hospital, School of Medicine, Tongji University, Shanghai, PR China; Urologic Cancer Institute, Tongji University School of Medicine, Shanghai, PR China; Department of Urology, Shanghai Tenth People’s Hospital, School of Medicine, Tongji University, Shanghai, PR China; Urologic Cancer Institute, Tongji University School of Medicine, Shanghai, PR China

## Abstract

**Background:**

Sepsis is a major cause of death in cancer patients, yet its variation by cancer type and patient characteristics remains underexplored. We analyzed sepsis mortality in a large cancer cohort, focusing on gender and demographic disparities.

**Methods:**

We analyzed 3 577 100 cancer cases from the SEER database (1975-2019) and calculated the standardized mortality ratio (SMR) and absolute excess risk (AER), stratified by gender, cancer type, and demographics. Logistic regression identified factors linked to sepsis mortality odds, while Cox proportional hazards models evaluated their time-dependent effects.

**Results:**

Cancer patients experienced an excess sepsis mortality rate of 1.68 deaths per 10 000 person-years compared to the general population. Among 11 926 cancer patients who died from sepsis (0.39% of 3.07 million cases), males had consistently higher mortality than females. Risk was highest in older adults, Black, unmarried, or widowed males with high-grade cancer. Liver and pancreatic cancers showed the highest SMR and AER, followed by stomach, lung, and hematologic cancers, whereas breast and prostate cancers had lower mortality. Patients diagnosed within the first year of cancer diagnosis faced the greatest risk. Logistic regression identified protective factors including female sex, younger age, localized cancer, marriage, and radiation therapy, while Cox models highlighted the time-dependent protective effects of these factors.

**Conclusions:**

Sepsis mortality varied significantly by gender, cancer type, and demographic characteristics. These findings emphasize the need for gender-specific and personalized management strategies to reduce sepsis mortality in high-risk cancer patients.

## Introduction

Cancer is a major social, public health, and economic issue in the 21st century, accounting for nearly one-sixth (16.8%) and one-quarter (22.8%) of non-communicable disease (NCD) deaths globally. By 2040, global cancer incidence is projected to rise to 28.4 million cases, a 47% increase from 2020, with a larger rise in transitioning countries owing to demographic changes and increasing risk factors.^1^ Sepsis, one of the leading causes of death worldwide, is responsible for up to 11 million deaths annually, surpassing cancer-related mortality.^2^ Cancer patients are especially prone to sepsis due to compromised immune function, with a higher incidence of infections, such as bacteremia and fungemia.^3-5^ Up to 21% of sepsis cases are associated with cancer.^6^ Studies have shown that sepsis mortality is significantly higher in cancer patients than in non-cancer patients, with 30% of hospitalized cancer patients dying from sepsis.^5^^,^^7^^,^^8^ Cancer treatment can suppress the host immune response, potentially increasing the risk of infections.^9^^,^^10^ Preclinical studies suggest cancer may also heighten sepsis mortality by disrupting the adaptive immune system.^11^ Given that cancer patients constitute a substantial proportion of the sepsis patient population, they represent a critical group that needs to be represented and characterized in sepsis-related clinical trials.

Although prior research has shed light on the relationship between sepsis and cancer,^12^ critical gaps remain, particularly in terms of gender-specific data, cancer-type differences, and the role of treatment modalities in sepsis outcomes. Our study leverages the latest SEER database to analyze sepsis-specific mortality in cancer patients with a primary focus on gender stratification, while also considering demographics, cancer type/stage, and treatments to enhance understanding of sepsis risk.

## Methods

### Data source and patient inclusion criteria

**Figure 1.**
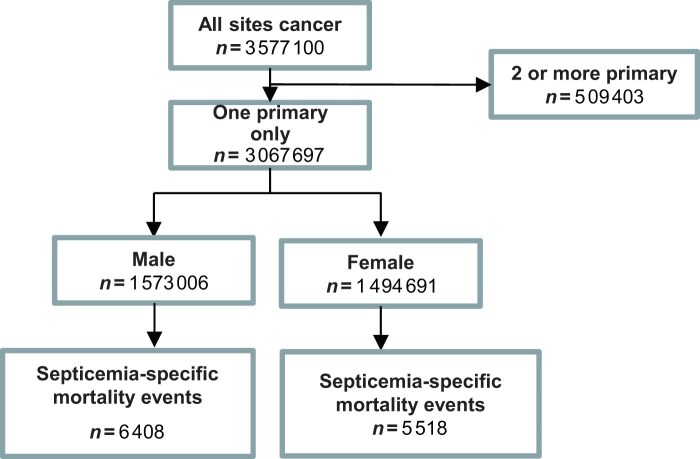
Flow diagram of the study selection process.

This study utilized data from the Surveillance, Epidemiology, and End Results (SEER) database. We extracted information on patients diagnosed with cancer between 1975 and 2019, including clinical and pathological details such as sex, race, tumor stage, metastatic status, age at diagnosis, date of diagnosis, calendar year, and follow-up status. Diagnoses were made according to the International Classification of Diseases for Oncology, 3rd Edition (ICD-O-3). To focus on sepsis-related mortality, we identified cases where sepsis was recorded as the cause of death. The inclusion criteria were as follows: (1) histologically confirmed single primary malignancy; (2) available follow-up information and cause of death; and (3) complete demographic and clinical variables including sex, race, tumor stage, and treatment details. The exclusion criteria were as follows: (1) diagnosis identified solely by autopsy or death certificate and (2) multiple primary tumors or missing essential clinical data. After applying these criteria, a total of 3 067 697 eligible patients were included in the final cohort for analysis. The patient selection flowchart is presented in Figure 1.

The SEER database used in this study contains publicly available, de-identified data (https://seer.cancer.gov/); therefore, ethical review was not required.

### Patient follow-up and sepsis identification

Patients were followed from 2 months after their cancer diagnosis until death or the end of the study period. Sepsis-related deaths were identified using the International Classification of Diseases, Ninth and Tenth Revisions (ICD-9: 995.91, 995.92; ICD-10: A40-A41). These codes were selected based on established sepsis identification algorithms used in previous epidemiologic studies (PMID: 29982318). The primary cancer site was classified based on the International Classification of Diseases for Oncology, Third Edition (ICD-O-3), as recorded in the SEER database. “Overall sepsis” includes all cases of sepsis recorded after cancer diagnosis, excluding any non-sepsis causes.

### Outcome measures

The primary outcomes of this study were the sepsis-specific standardized mortality ratio (SMR) and the absolute excess risk (AER) of sepsis among cancer patients. SMRs were calculated against the age-, sex-, and calendar year-specific mortality rates in the general US population (National Center for Health Statistics). Stratified analyses were conducted to explore potential heterogeneity in sepsis-related mortality across clinically relevant subgroups, including age group, sex, cancer type, race and ethnicity, tumor stage and grade, treatment modalities (surgery, chemotherapy, radiotherapy), marital status, and socioeconomic factors. These subgroup comparisons aimed to identify population-level disparities and high-risk profiles associated with increased sepsis mortality in cancer patients.

In SEER, treatment variables are abstracted from medical records and reflect whether patients received surgery, chemotherapy, or radiotherapy as part of their initial course of therapy. Each treatment modality is recorded independently, a value of “Yes” indicates that the patient received the specified treatment, either alone or in combination with other modalities.

### Statistical analysis

Descriptive statistics were used to summarize baseline demographic and clinical characteristics. Categorical variables were expressed as counts and percentages, and differences across groups were assessed using the chi-square test. Continuous variables were summarized as means with standard deviations or medians with interquartile ranges, depending on data distribution, and compared using the *t* test or Mann-Whitney U test as appropriate.

Sepsis-specific mortality rate was calculated by dividing the number of sepsis-related deaths by total person-years at risk and multiplying by 10 000. The SMR and AER were estimated using indirect standardization, based on age-, sex-, and calendar year-specific mortality rates from the general US population, as obtained from SEER-linked national vital statistics, with 95% confidence intervals (CIs) derived from Poisson regression. Subgroup analyses were conducted by sex, cancer type, age, treatment modality, tumor grade, and sociodemographic factors. Multivariable logistic regression models were applied to estimate adjusted odds ratios (ORs) with 95% CIs for factors associated with sepsis-related mortality, treating it as a binary outcome over the entire study period. A multivariable Cox proportional hazards model was used to estimate hazard ratios (HRs) for sepsis-related mortality. Deaths from causes other than sepsis were treated as censored observations, consistent with a cause-specific hazard modeling framework. Presenting both models provides complementary perspectives: logistic regression reflects overall occurrence risk, while Cox regression captures temporal dynamics and hazard over follow-up.

Confounding variables adjusted for in both regression models included age at diagnosis, sex, race, year of diagnosis, marital status, tumor grade and stage, treatment modalities (surgery, radiotherapy, chemotherapy), and socioeconomic variables (household income and urban/rural classification). These covariates were selected a priori based on clinical relevance and prior studies demonstrating associations with sepsis outcomes. Model diagnostics, including Schoenfeld residuals, were performed to confirm assumptions.

All statistical analyses were performed using SEER*Stat software (version 8.3.9), IBM SPSS Statistics (version 26), and R software (version 4.0.3). A 2-tailed *P* value less than .05 was considered statistically significant.

## Results

### Patient characteristics

Overall, 3 067 697 cancer patients were included in this study, with a nearly even distribution between sexes (51.28% male and 48.72% female), ranging in age from 0 to 80+ years (Table 1). Among these patients, 11 926 (0.39%) deaths were attributed to sepsis. Of these sepsis-related deaths, 78.61% (*n* = 9375) occurred in patients aged ≥ 60 years. The sepsis-specific mortality rate was 204.59 per 100 000 person-years, and the average survival time following cancer diagnosis was 5.90 years. The overall SMR for the population was 1.40 (95% CI = 1.37 to 1.42), and the AER per 10 000 person-years was 1.68 (95% CI = 1.54 to 1.82). We conducted a separate analysis for male and female cancer patients, and the results showed that compared to female cancer patients, male cancer patients had a higher probability of sepsis-related mortality. Variations in sepsis-related mortality were observed based on age, race, marital status, year of diagnosis, tumor stage, and treatment modality, such as surgery, chemotherapy, and radiation (Tables S1 and S2).

**Table 1.** Sepsis-related SMRs and AERs per 10 000 person-years by patient characteristics for all cancers combined.

Characteristics	Patients with cancer, *n* (%)	Death of sepsis, *n* (%)	Proportion of sepsis in all cause death (%)	Mortality of sepsis (per 100 000)	Person-years	SMR (95% CI)	AER (95% CI)
Total	3 067 697 (100)	11 926 (100)	0.61	204.59	582 916.58	1.40* (1.37 to 1.42)	1.68 (1.54 to 1.82)
Sex							
Male	1 573 006 (51.28)	6408 (53.73)	0.61	6.84	9 370 220.61	1.41* (1.38 to 1.45)	2 (1.78 to 2.22)
Female	1494691 (48.72)	5518 (46.27)	0.61	5.08	10858486.12	1.38* (1.35 to 1.42)	1.41 (1.23 to 1.59)
Age at diagnosis							
00-19	42 842 (1.4)	46 (0.39)	0.42	0.79	582 916.58	10.01* (7.33 to 13.35)	0.71 (0.47 to 0.95)
20-29	61 309 (2)	77 (0.65)	0.5	0.91	841 848.8	3.59* (2.83 to 4.48)	0.66 (0.43 to 0.89)
30-39	137 708 (4.49)	189 (1.58)	0.42	1.16	1 626 902.16	2.25* (1.94 to 2.59)	0.64 (0.44 to 0.84)
40-49	282 852 (9.22)	596 (5)	0.52	2.11	2 820 477.92	1.99* (1.84 to 2.16)	1.05 (0.84 to 1.26)
50-59	555511 (18.11)	1643 (13.78)	0.58	3.73	4399502.82	1.70* (1.62 to 1.78)	1.54 (1.31 to 1.77)
60-69	794972 (25.91)	3208 (26.9)	0.64	6.26	5122567.37	1.42* (1.37 to 1.47)	1.85 (1.57 to 2.13)
70-79	730681 (23.82)	3757 (31.5)	0.66	10.54	3564316.21	1.26* (1.22 to 1.3)	2.15 (1.7 to 2.6)
80+	461822 (15.05)	2410 (20.21)	0.58	18.97	1270174.88	1.27* (1.22 to 1.32)	4.06 (3.05 to 5.07)
Race							
White	2569722 (83.77)	9564 (80.19)	0.58	5.52	17331684.71	1.30* (1.28 to 1.33)	1.28 (1.13 to 1.43)
Black	229719 (7.49)	1373 (11.51)	0.96	10.94	1255507.19	1.76* (1.66 to 1.85)	4.7 (3.98 to 5.42)
Other (American Indian/AK Native-Asian/Pacific Islander)	268256 (8.74)	989 (8.29)	0.67	6.02	1641514.84	2.51* (2.35 to 2.67)	3.62 (3.18 to 4.06)
Marital status at diagnosis							
Single	419723 (13.68)	1424 (11.94)	0.64	9.16	1555418.91	2.25* (2.13 to 2.37)	2.67 (2.37 to 2.97)
Married	1714054 (55.87)	6300 (52.83)	0.6	10.22	6161689.58	1.22* (1.19 to 1.25)	0.92 (0.75 to 1.09)
Unmarried or Domestic Partner	4723 (0.15)	6 (0.05)	0.5	9.21	6517.78	2.48 (0.91 to 5.4)	2.85 (-1.68 to 7.38)
Divorced / Separated / Widowed	739367 (24.1)	3437 (28.82)	0.59	13.4	2565722.14	1.58* (1.53 to 1.64)	3.61 (3.19 to 4.03)
Unknown	189830 (6.19)	759 (6.36)	0.84	13.34	569137.7	1.35* (1.26 to 1.45)	1.54 (0.98 to 2.1)
Year of diagnosis							
1975-1979	215 917 (7.04)	749 (6.28)	0.37	4.52	1 658 186.17	1.27* (1.18 to 1.36)	0.95 (0.52 to 1.38)
1980-1989	519 135 (16.92)	2542 (21.31)	0.54	6.26	4 060 270.29	1.43* (1.38 to 1.49)	1.9 (1.58 to 2.22)
1990-1999	646 535 (21.08)	3287 (27.56)	0.63	5.88	5 594 256.14	1.22* (1.17 to 1.26)	1.04 (0.77 to 1.31)
2000-2009	766 810 (25)	3342 (28.02)	0.73	5.66	5 904 982.62	1.36* (1.32 to 1.41)	1.51 (1.26 to 1.76)
2010-2019	919 300 (29.97)	2006 (16.82)	0.66	6.66	3 011 011.51	2.01* (1.92 to 2.09)	3.34 (2.98 to 3.7)
Grade							
Grade I	264 685 (9.59)	1378 (12.22)	0.99	5.37	2 568 319.7	1.14* (1.08 to 1.2)	0.67 (0.28 to 1.06)
Grade II	688 779 (24.95)	3375 (29.92)	0.83	5.72	5 900 126.78	1.11* (1.07 to 1.14)	0.55 (0.28 to 0.82)
Grade III	560 197 (20.3)	2078 (18.42)	0.53	6.2	3 349 056.8	1.38* (1.32 to 1.44)	1.71 (1.36 to 2.06)
Grade IV	128 856 (4.67)	384 (3.4)	0.36	8.01	479 356.29	2.16* (1.95 to 2.38)	4.3 (3.33 to 5.27)
Unknown	1 117 727 (40.49)	4066 (36.04)	0.5	5.73	7 092 089.87	1.77* (1.72 to 1.83)	2.5 (2.28 to 2.72)
Stage							
Localized	770 658 (196.93)	3909 (246.94)	1	4.4	8 874 947.82	1.11* (1.07 to 1.14)	0.43 (0.24 to 0.62)
Regional	495 386 (126.59)	2246 (141.88)	0.62	6.44	3 489 843.19	1.76* (1.69 to 1.83)	2.78 (2.45 to 3.11)
Distant	549 584 (140.44)	1484 (93.75)	0.3	10.58	1 402 653.64	3.28* (3.11 to 3.45)	7.35 (6.74 to 7.96)
Localized/regional (Prostate cases)	227 922 (58.24)	1004 (63.42)	1.21	4.17	2 405 141.42	0.70* (0.66 to 0.75)	−1.76 (−2.16 to −1.36)
Unstaged	391 335 (100)	1583 (100)	0.52		2 027 930.4	2.42* (2.31 to 2.55)	4.59 (4.13 to 5.05)
Surgery							
No	3 067 697 (63.91)	11 926 (62.12)	0.61	5.9	2 022 8706.74	1.40* (1.37 to 1.42)	1.68 (1.54 to 1.82)
Yes	1 732 134 (36.09)	7272 (37.88)	0.79	4.7	15 471 047.46	1.18* (1.16 to 1.21)	0.72 (0.57 to 0.87)
Radiation recode							
No	896 287 (29.22)	2910 (24.4)	0.53	4.93	5 901 659.62	1.43* (1.4 to 1.46)	1.89 (1.72 to 2.06)
Yes	2 171 410 (70.78)	9016 (75.6)	0.64	6.29	1 432 7047.11	1.32* (1.27 to 1.37)	1.19 (0.95 to 1.43)
Chemotherapy recode							
No	2 230 227 (72.7)	9596 (80.46)	0.68	6.02	15 940 997.83	1.28* (1.25 to 1.3)	1.31 (1.15 to 1.47)
Yes	837 470 (27.3)	2330 (19.54)	0.43	5.43	4 287 708.91	2.29* (2.2 to 2.38)	3.06 (2.8 to 3.32)

Abbreviations: SMR = standardized mortality ratio; AER = absolute excess risk.

* 95% confidence interval does not include 1.0, indicating statistical significance.

Reference population: General US population, adjusted for age, sex, and calendar year (2000 US standard population).

Patients: Histologically confirmed single primary cancers in SEER.

### Sex-specific differences in sepsis mortality

Sepsis mortality rates varied across cancer types and sexes. Males showed higher mortality in liver cancer (SMR = 9.57, AER = 21.9), pancreatic cancer (SMR = 6.94, AER = 21.59), lung cancer (SMR = 3.81, AER = 11.51), stomach cancer (SMR = 3.03), colorectal cancer (SMR = 1.64), and leukemia (SMR = 2.29) than females. Conversely, females exhibited higher mortality in ALL (SMR = 13.59, AER = 2.39), tonsil cancer (SMR = 4.37, AER = 8.76), laryngeal cancer (SMR = 2.20, AER = 4.13), soft tissue cancers (SMR = 1.47), and brain cancer (SMR = 5.59, AER = 2.998). Sex-specific cancers showed varying patterns: prostate cancer had lower mortality (SMR = 0.84), while cervical and uterine cancers showed higher risks (SMR = 2.28 and 1.24, respectively). Breast cancer in females demonstrated lower mortality (SMR = 0.97), comparable to prostate cancer in males. These sex-specific differences warranted further investigation into cancer-sepsis relationships (Table S3).

### Impact of age on sepsis-related mortality across cancer types

**Figure 2.**
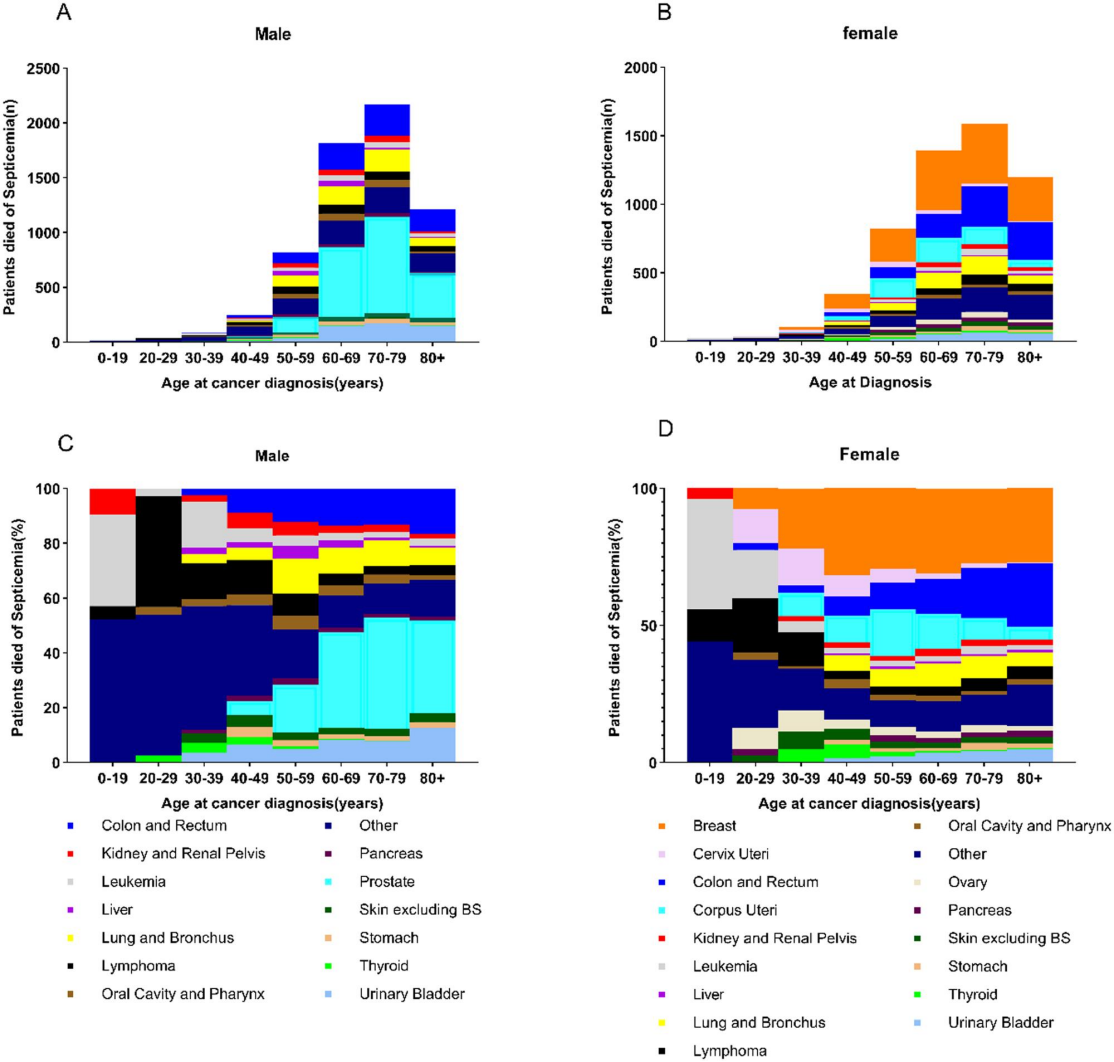
Sepsis-related mortality and cancer type distribution by age and sex. Sepsis-related deaths in male **(A)** and female **(B)** cancer patients across age groups. Cancer type distribution among male **(C)** and female **(D)** cancer patients who died of sepsis, stratified by age at diagnosis.

In our analysis, age played a significant role in influencing the distribution of cancer types associated with sepsis-related death (Figure 2). As patients aged, the absolute number of deaths due to sepsis increased, particularly in the 60-79 age group, for both male and female cancer patients. Colorectal cancer, liver cancer, and leukemia were the most common types of cancer contributing to sepsis-related mortality across all age groups. However, in females, breast cancer also became a dominant contributor in older age groups, while prostate cancer was prominent among older males. The data indicate that younger patients had a more limited range of cancers associated with sepsis-related deaths, primarily involving leukemia, but as age increased, the diversity of cancer types leading to sepsis-related mortality expanded, particularly in patients aged 50 years and above (Tables S4 and S5).

### Sepsis mortality risk among cancer patients following diagnosis

**Figure 3.**
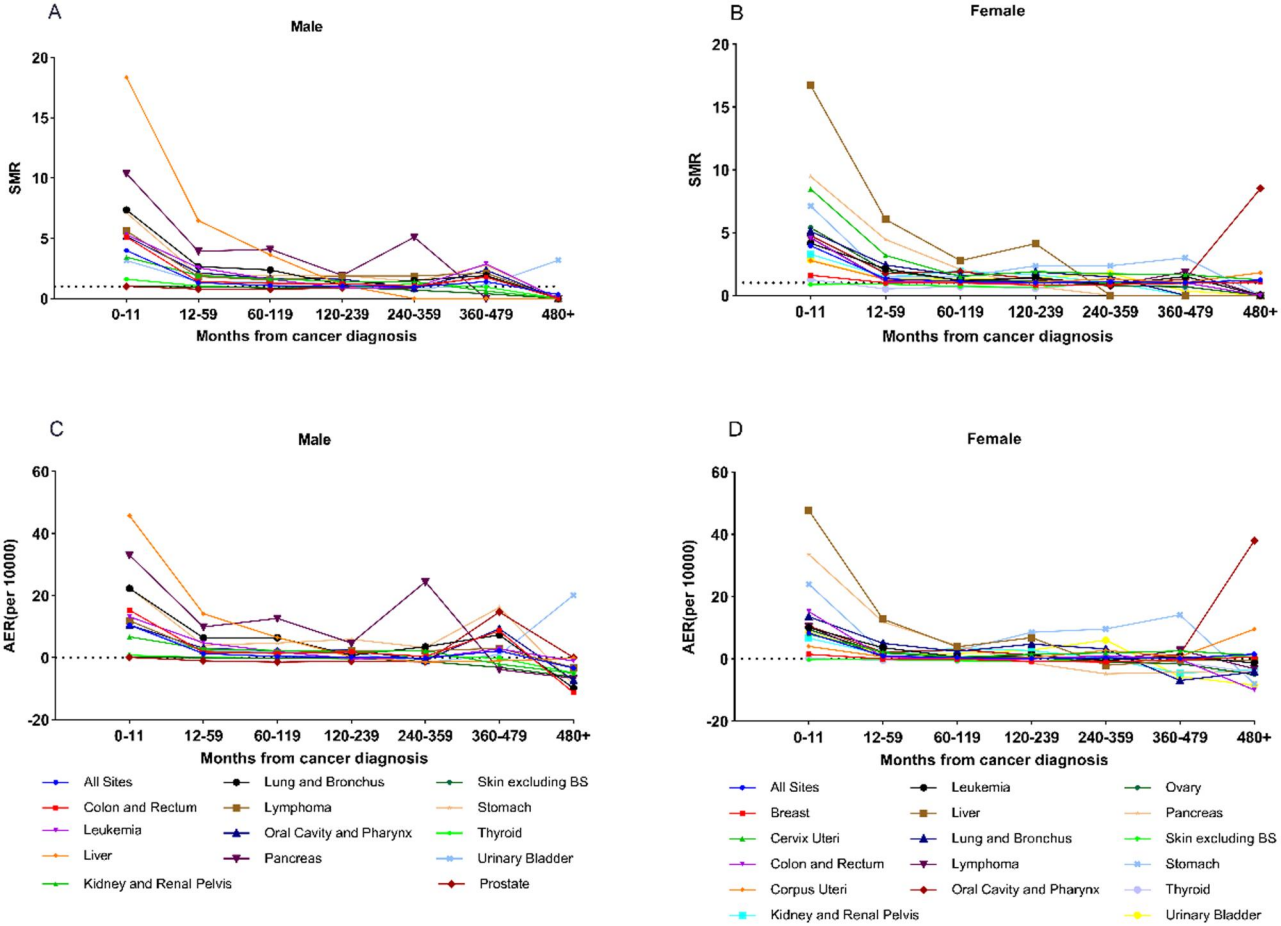
Sepsis-specific mortality trends by time since cancer diagnosis and sex. Sepsis-specific SMR trends in male **(A)** and female **(B)** cancer patients at different time intervals post-diagnosis. AER trends in male **(C)** and female **(D)** cancer patients at different time intervals post-diagnosis.

Temporal analysis of sepsis-specific mortality among cancer patients showed peak SMRs and AERs in the first year post-diagnosis in both sexes, followed by gradual decline (Figure 3). Males with pancreatic cancer and females with stomach cancer showed SMR/AER fluctuations over time. Liver cancer demonstrated particularly high early mortality, with SMRs of 18.35 (males) and 16.72 (females), and AERs of 45.75 and 47.68 per 10 000 person-years, respectively. During the early post-diagnosis period, liver, colorectal, lung, urinary bladder, and pancreatic cancers were most strongly associated with sepsis mortality in both sexes, underscoring the varying contributions of different cancers to sepsis mortality risk over time (Tables S6 and S7).

### Sepsis mortality risk among cancer patients by year of diagnosis

**Figure 4.**
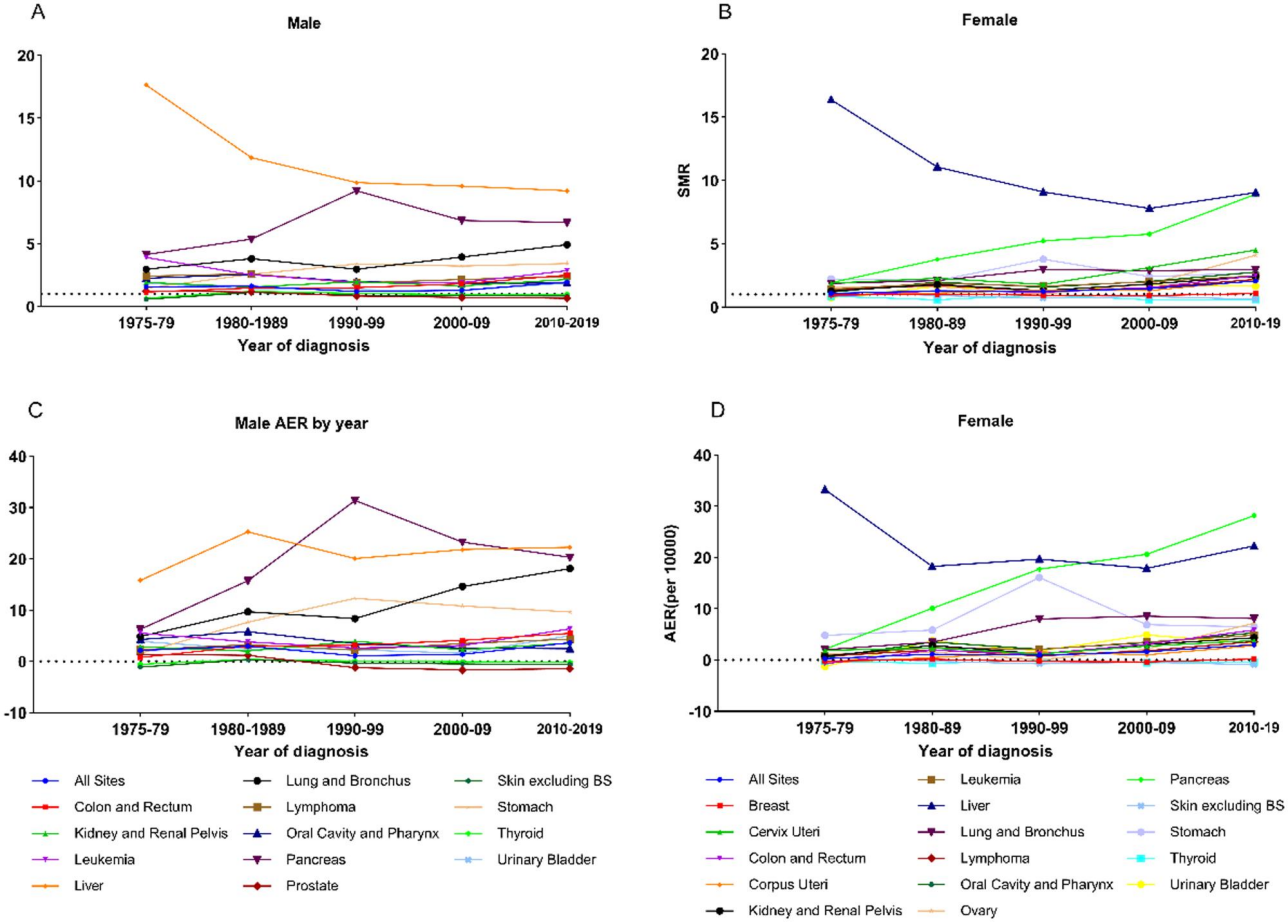
Temporal trends in sepsis-specific mortality by sex and year of diagnosis. Sepsis-specific SMR trends for male **(A)** and female **(B)** cancer patients across different decades of diagnosis. AER trends for male **(C)** and female **(D)** cancer patients by year of diagnosis.

We analyzed the temporal trends in cancer-related sepsis mortality stratified by sex over a 45-year period (1975-2019) (Figure 4). While liver, lung, and stomach cancers consistently showed strong associations with sepsis mortality, their impact diminished over time. Liver cancer demonstrated the most significant association, with male patients experiencing a notable SMR decrease from 17.64 to 9.22 between 1975-1979 and 2010-2019, although their AER increased from 15.83 to 22.99 per 10 000 person-years. Female liver cancer patients showed comparable declines in mortality rates (SMR decreased from 16.38 to 9.04; AER dropped from 33.28 to 22.27). In contrast, pancreatic cancer exhibited an increasing trend in mortality risk across both sexes, with 2010-2019 rates suggesting a potential to surpass liver cancer mortality in the future (males: SMR = 6.69, AER = 20.33; females: SMR = 8.91, AER = 28.17 per 10 000 person-years) (Tables S8 and S9).

### Sepsis mortality risk among cancer patients by treatment modality

**Figure 5.**
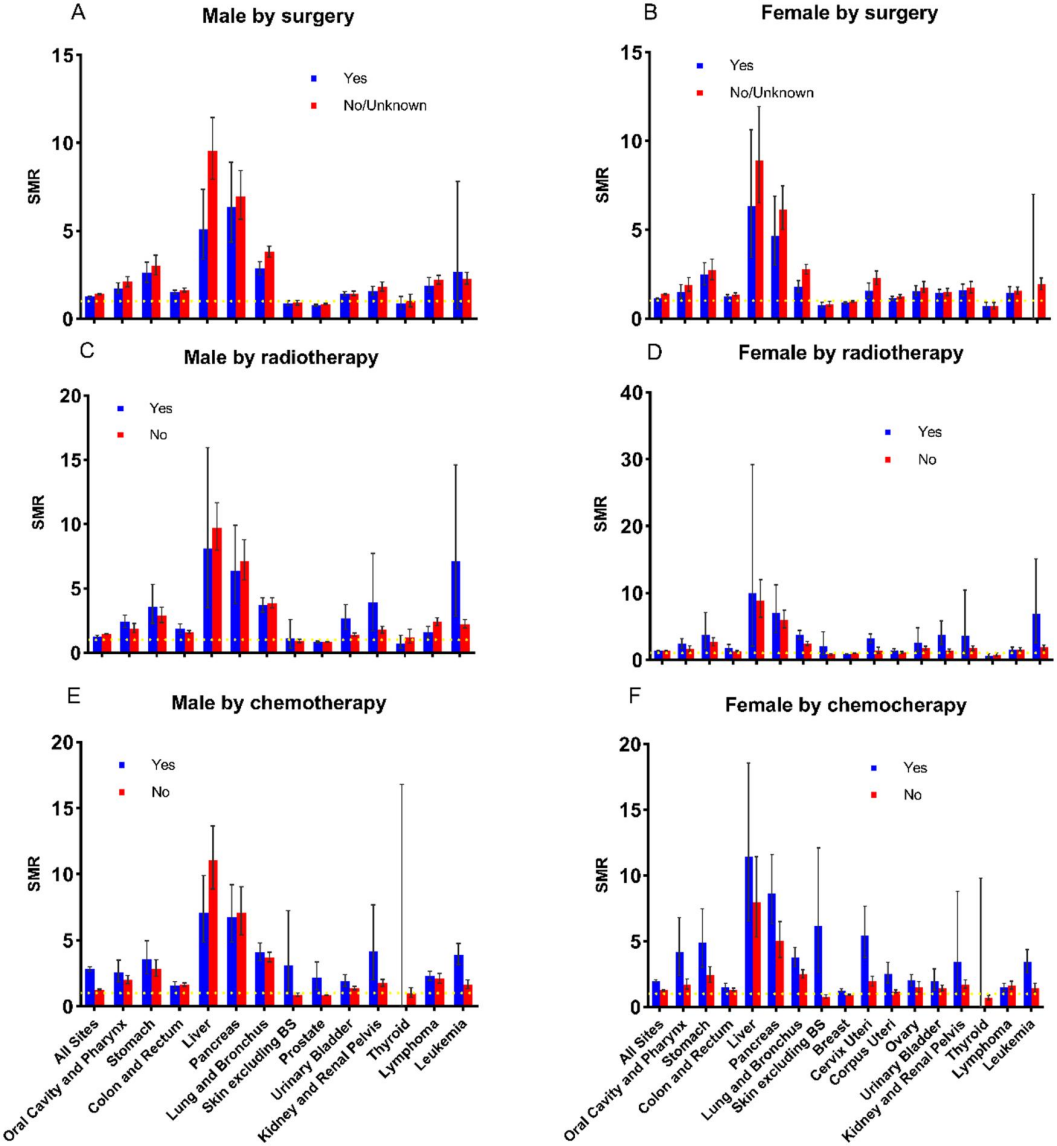
Gender-specific sepsis SMRs among cancer patients stratified by treatment modality. **(A and B)** SMRs for male and female cancer patients by surgery status (Yes vs. No/Unknown). **(C and D)** SMRs for male and female cancer patients by radiotherapy status (Yes vs. No). **(E and F)** SMRs for male and female cancer patients by chemotherapy status (Yes vs. No).

The impact of different treatment modalities on sepsis-specific mortality among cancer patients varied by sex (Figure 5). Both male and female patients who did not undergo surgery exhibited higher SMR and AER for sepsis-related mortality (Tables S10 and S11). Notable sex-specific differences were observed between radiotherapy and chemotherapy. The effect of radiotherapy and chemotherapy on sepsis-specific mortality was most pronounced in leukemia, skin excluding basal cell carcinoma (BS), and the kidney and renal pelvis. For females, most cancers showed a significant reduction in sepsis mortality after receiving radiotherapy and chemotherapy. However, in males, the pattern differed—particularly in liver and pancreatic cancers, where sepsis-related mortality increased following radiotherapy and chemotherapy (Tables S12 and S15). This indicates that while these treatments reduce sepsis risk in females across several cancer types, in males, they may contribute to a heightened risk of sepsis mortality in liver and pancreatic cancers, underscoring the need for sex-specific management strategies to mitigate sepsis risk after treatment.

### Variations in sepsis mortality risk by demographics and cancer characteristics

**Figure 6.**
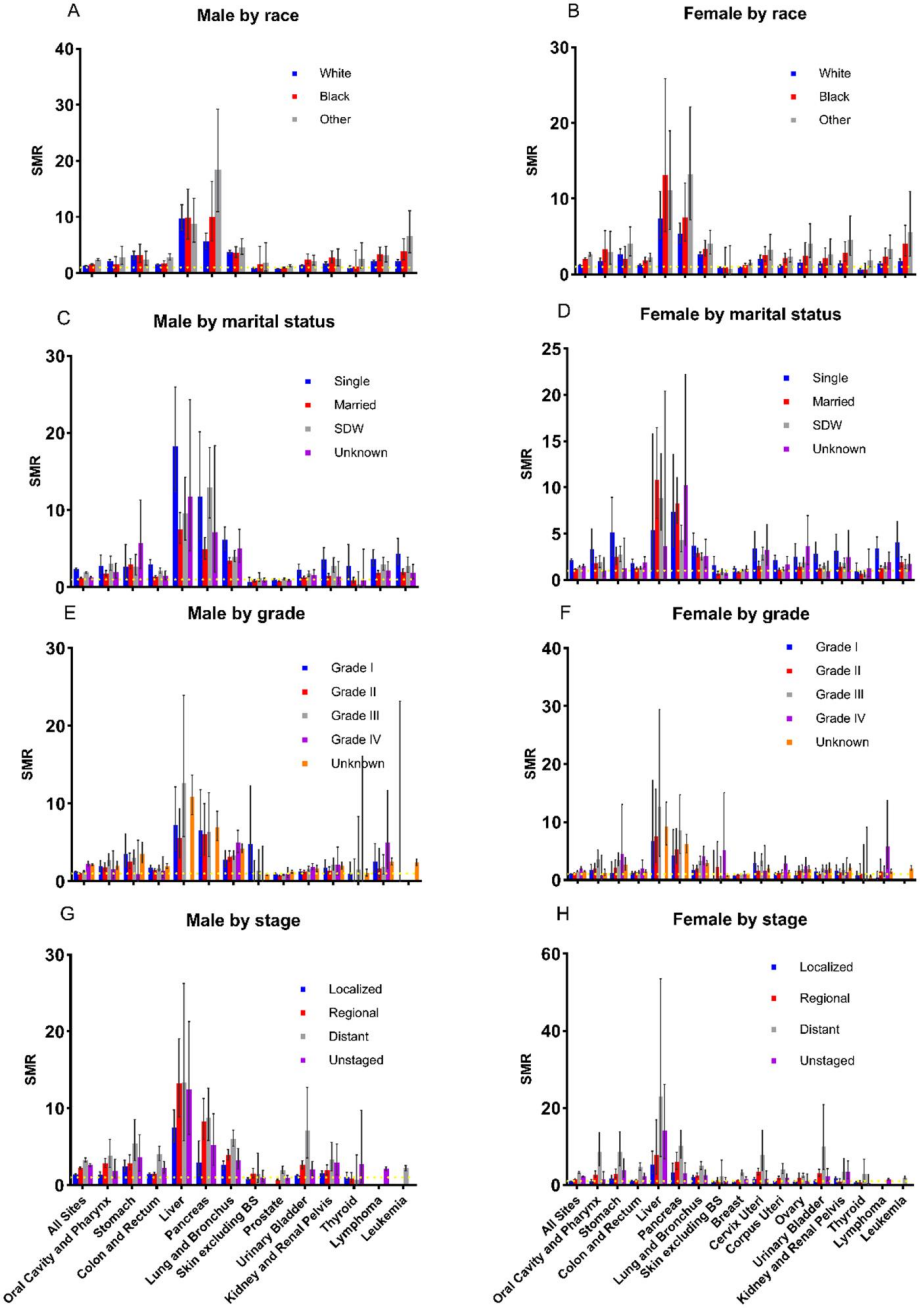
Gender-specific sepsis SMRs among cancer patients stratified by demographic and clinical factors. **(A and B)** SMRs for male and female cancer patients by race. **(C and D)** SMRs for male and female cancer patients by marital status. **(E and F)** SMRs for male and female cancer patients by tumor grade. **(G and H)** SMRs for male and female cancer patients by stage at diagnosis.

In analyzing all cancer types, disparities in demographics and cancer characteristics were evident in sepsis mortality (Figure 6). “Other” racial groups showed the highest SMR (males: SMR = 2.42 [95% CI = 2.22 to 2.63], AER = 4.56; females: SMR = 2.62 [95% CI = 2.38 to 2.87], AER 2.96), followed by Black patients, while White patients had the lowest rates (Tables S16 and S17). Marital status significantly influenced sepsis mortality. Single patients demonstrated the highest mortality rates (males: SMR = 2.35 [95% CI = 2.18 to 2.52], AER = 3.07; females: SMR = 2.14 [95% CI = 1.98 to 2.31], AER = 2.31), followed by widowed/divorced patients (Tables S18 and S19). These associations varied by cancer site, with notable exceptions in pancreatic cancer, where widowed males and married females showed the highest mortality. Regarding tumor characteristics, Grade IV tumors had the highest mortality rates (males: SMR = 2.29 [95% CI = 1.98 to 2.62], AER = 5.21; females: SMR = 1.52 [95% CI = 1.42 to 1.62], AER = 1.85), while Grade I tumors showed the lowest rates (Tables S20 and S21). Distant metastasis generally showed the highest mortality, except in male liver and pancreatic cancers, where localized metastasis had higher rates (Tables S22 and S23).

### Influential factors in sepsis mortality: logistic and Cox model analysis

According to the logistic regression and Cox proportional hazards models, multiple factors significantly influenced the risk of sepsis-related mortality among cancer patients (Table 2, and Tables S24 and S25). Age, race, gender, year of diagnosis, tumor grade, cancer stage, and treatment type all had an impact, with gender showing a particularly notable effect; females had a lower risk compared to males (OR = 0.86, HR = 0.68, *P* < .001). Additionally, older age, Black race (OR = 1.94, HR = 2.25, *P* < .001) and other minority groups compared to White, earlier year of diagnosis, higher tumor grade, and advanced cancer stage were all associated with an increased risk of mortality. Radiation treatment, compared to no radiation treatment, appeared to have a protective effect (OR = 0.85, HR = 0.90, *P* < .001), while surgery was associated with a slightly elevated risk. Socioeconomic factors, including lower income and unmarried status, were also associated with higher mortality rates. However, the median household income and rural-urban continuum code generally lacked statistical significance across most categories (*P* > .05), suggesting that these variables may have limited influence on sepsis mortality risk in this population. This analysis underscores the complex interplay of demographic, clinical, and socioeconomic factors in sepsis-related mortality among cancer patients.

**Table 2.** Odds ratios (ORs) and hazard ratios (HRs) for sepsis-related mortality among all cancer patients.

	Logistic regression model		Cox proportional hazards model	
Variables	OR (95% CI)	*P*	HR (95% CI)	*P*
Age at diagnosis				
0-19	1		1	
20-29	1.08 (0.75 to 1.56)	.681	1.93 (1.32 to 2.83)	.001
30-39	1.22 (0.88 to 1.69)	.232	3.08 (2.19 to 4.35)	< .001
40-49	1.93 (1.43 to 2.62)	< .001	6.45 (4.67 to 8.91)	< .001
50-59	2.90 (2.15 to 3.91)	< .001	12.84 (9.35 to 17.64)	< .001
60-69	4.07 (3.03 to 5.48)	< .001	23.86 (17.38 to 32.76)	< .001
70-79	5.04 (3.74 to 6.78)	< .001	42.37 (30.84 to 58.21)	< .001
80+	5.13 (3.81 to 6.92)	< .001	72.02 (52.29 to 99.20)	< .001
Race				
White	1		1	
Black	1.94 (1.83 to 2.06)	< .001	2.25 (2.11 to 2.39)	< .001
Other (American Indian/AK Native, Asian/Pacific Islander)	1.08 (1.00 to 1.16)	.038	1.08 (1.00 to 1.16)	.053
Unknown	0.33 (0.21 to 0.51)	< .001	0.31 (0.19 to 0.51)	< .001
Sex				
Male	1		1	
Female	0.86 (0.83 to 0.90)	< .001	0.69 (0.66 to 0.72)	< .001
Year of diagnosis				
1975-1979	1		1	
1980-1989	1.38 (1.27 to 1.50)	< .001	1.33 (1.23 to 1.45)	< .001
1990-1999	2.09 (1.04 to 4.22)	.039	2.07 (1.03 to 4.16)	.042
2000-2009	1.83 (0.91 to 3.69)	.091	2.26 (1.12 to 4.54)	.022
2010-2019	0.96 (0.48 to 1.95)	.918	2.25 (1.11 to 4.53)	.024
Grade				
Grade I	1		1	
Grade II	0.91 (0.85 to 0.97)	.004	0.99 (0.93 to 1.06)	.869
Grade III	0.76 (0.71 to 0.81)	< .001	1.09 (1.02 to 1.17)	.016
Grade IV	0.67 (0.60 to 0.75)	< .001	1.26 (1.12 to 1.43)	< .001
Unknown	0.82 (0.77 to 0.88)	< .001	1.01 (0.95 to 1.08)	.742
Stage				
Localized	1		1	
Regional	0.88 (0.83 to 0.93)	< .001	1.33 (1.26 to 1.41)	< .001
Distant	0.76 (0.70 to 0.82)	< .001	0.48 (0.44 to 0.52)	< .001
Unstaged	0.51 (0.48 to 0.55)	< .001	1.35 (1.25 to 1.45)	< .001
Localized/regional (Prostate cases)	0.68 (0.65 to 0.72)	< .001	1.02 (0.96 to 1.08)	0.577
Surgery				
No	1		1	
Yes	1.06 (1.01 to 1.11)	.011	0.65 (0.61 to 0.68)	< .001
Radiation recode				
No	1		1	
Yes	0.85 (0.82 to 0.89)	< .001	0.90 (0.86 to 0.95)	< .001
Chemotherapy recode				
No	1		1	
Yes	0.96 (0.91 to 1.01)	.133	1.14 (1.08 to 1.20)	< .001
Marital status at diagnosis				
Single	1		1	
Married	0.91 (0.87 to 0.96)	< .001	0.73 (0.70 to 0.77)	< .001
Unmarried or Domestic Partner	1.11 (1.04 to 1.18)	.002	1.09 (1.01 to 1.16)	.018
Divorced/Separated/Widowed	1.04 (0.96 to 1.13)	.327	0.79 (0.73 to 0.86)	< .001
Unknown	0.71 (0.32 to 1.58)	.397	0.96 (0.43 to 2.14)	.916
Median household income				
< $35 000	1		1	
$35 000 - $39 999	0.74 (0.47 to 1.17)	.202	0.84 (0.52 to 1.36)	.48
$40 000 - $44 999	0.90 (0.62 to 1.32)	.604	0.86 (0.57 to 1.30)	.475
$45 000 - $49 999	0.91 (0.63 to 1.31)	.608	0.84 (0.56 to 1.24)	.369
$50 000 - $54 999	0.85 (0.60 to 1.21)	.375	0.79 (0.53 to 1.15)	.217
$55 000 - $59 999	0.82 (0.57 to 1.17)	.275	0.73 (0.49 to 1.07)	.104
$60 000 - $64 999	0.99 (0.69 to 1.41)	.954	0.90 (0.61 to 1.32)	.588
$65 000 - $69 999	0.97 (0.68 to 1.38)	.864	0.91 (0.62 to 1.33)	.623
$70 000 - $74 999	1.23 (0.86 to 1.75)	.256	1.14 (0.78 to 1.68)	.498
$75 000+	0.93 (0.65 to 1.32)	.681	0.84 (0.57 to 1.23)	.368
Rural-Urban Continuum Code				
Counties in metropolitan areas greater than 1 million population	1		1	
Counties in metropolitan areas of 250 000 to 1 million population	1.37 (1.30 to 1.45)	< .001	1.40 (1.32 to 1.47)	< .001
Counties in metropolitan areas of less than 250 thousand population	0.87 (0.78 to 0.96)	.007	0.88 (0.79 to 0.98)	.023
Non-metropolitan counties adjacent to a metropolitan area	1.23 (1.12 to 1.36)	< .001	1.23 (1.11 to 1.36)	< .001
Non-metropolitan counties not adjacent to a metropolitan area	1.08 (0.96 to 1.21)	.21	1.10 (0.98 to 1.25)	.11

Abbreviations: OR = odds ratio; HR = hazard ratio; CI = confidence interval.

Reference categories: Age 0-19, White race, Male sex, Year 1975-1979, Grade I, Localized stage, No treatment (for surgery/radiation/chemotherapy), Single marital status, median household income <$35 000, and metropolitan counties ≥1 million population.

An OR or HR >1.0 indicates increased risk; <1.0 indicates decreased risk. A 95% CI not including 1.0 and *P* < .05 denote statistical significance.

## Discussion

Previous research has revealed a bidirectional relationship between cancer and sepsis. Cancer and its treatments can weaken the immune system and disrupt mucosal barriers, thereby increasing susceptibility to sepsis.^13-15^ Conversely, sepsis may promote cancer development through mechanisms such as persistent immune dysfunction, chronic inflammation, and impaired tumor surveillance.^16^ Sepsis survivors often experience long-term organ dysfunction and immunosuppression, further elevating cancer risk.^17^^,^^18^ With global cancer incidence projected to reach 35 million annually by 2050,^19^ clarifying the association between sepsis and cancer is critical for improving clinical management and informing future research.

In this large-scale cohort study of over 3.5 million cancer patients, we systematically analyzed sepsis-specific mortality and observed marked variations based on cancer type, time since diagnosis, age, and race. A key finding was the consistently higher sepsis-related mortality in male cancer patients across both solid and hematologic malignancies, which may be attributed to biological differences or differential treatment responses. Sepsis risk also increased with age, with patients aged 70-79 exhibiting the highest mortality—particularly from gastrointestinal cancers such as liver and pancreatic malignancies. In contrast, younger patients (<40 years) were more likely to die from sepsis secondary to hematologic cancers, consistent with the age distribution of these diseases.

In 2018, 13% of new cancer cases globally were attributed to infectious pathogens.^20^ Chronic inflammation is considered a hallmark of malignancy.^21^^,^^22^ Our study demonstrates that sepsis poses a higher mortality risk in both digestive and respiratory system cancers, particularly in liver, lung, pancreatic, gastric, and esophageal cancers. This aligns with prior evidence indicating that bacterial infections can increase the risk of sepsis in these cancers.^23^^,^^24^ Epidemiological studies have shown that *Chlamydia pneumoniae* infection is associated with an increased risk of lung cancer, potentially leading to severe sepsis.^24^ Respiratory infections might synergize with carcinogens in tobacco smoke, exacerbating lung damage.^25^ Sepsis might also indicate the presence of precancerous conditions, such as *Helicobacter pylori* infection being a significant factor in gastric cancer, and Enterobacteriaceae infections being common in cirrhosis patients (a key liver cancer precursor).^26-28^ Moreover, patients with *Streptococcus gallolyticus* bacteremia, endocarditis, or sepsis often have precancerous colonic polyps.^22^^,^^29^^,^^30^ Inflammatory bowel disease patients also face an increased risk of hematological cancers, with a marked rise in myeloid leukemia incidence in ulcerative colitis (UC) and a slight increase in lymphoma risk in Crohn’s disease (CD).^31-33^

Hematologic malignancies were also associated with substantial sepsis mortality. Notably, patients with acute lymphoblastic leukemia (ALL) had SMRs of 11.26 and 13.59 in males and females, respectively. Individuals with myeloid leukemias (AML, CML, and MDS) are particularly vulnerable to infection and sepsis due to treatment-induced neutropenia.^34^ Sepsis is a leading cause of ICU mortality in patients with hematologic malignancies,^35^ with hospital-acquired infections (HAIs) frequently involving resistant pathogens such as *Klebsiella pneumoniae*, *Pseudomonas aeruginosa*, and *Escherichia coli*.^36^ Bone marrow transplant recipients are especially high-risk due to post-transplant immunosuppression and delayed immune reconstitution.^37^ Elevated inflammatory scores have been linked to shorter survival in AML, suggesting a potential role for anti-inflammatory therapies in leukemia management.^38^ Additionally, sepsis-induced immune dysregulation and cytokine overproduction may contribute to leukemia pathogenesis.^16^

Our results show that the highest sepsis-related mortality occurred within the first year after cancer diagnosis, followed by a gradual decline over the next 2 decades. This trend may reflect the increased hospitalization and treatment intensity during the initial phase, particularly chemotherapy-induced neutropenia, which heightens infection risk. Use of prophylactic antibiotics and G-CSF is especially critical during this period.^39^ Over time, improved infection control and supportive care have likely reduced long-term risk. Temporal trends also revealed that patients diagnosed between 1975 and 1989 had higher sepsis-related SMRs than those diagnosed in later decades, reflecting advances in cancer therapy and sepsis management. Nonetheless, certain malignancies—particularly liver, lung, and pancreatic cancers—remain associated with persistently high risk, underscoring the need for continued improvements in supportive care.

Multivariable logistic and Cox regression models further supported the substantial influence of demographic and clinical variables on sepsis mortality. Demographic disparities were pronounced: Black and minority patients, as well as those who were single, widowed, or divorced, had significantly higher mortality. Clinically, advanced-stage (or unstaged) tumors were associated with greater sepsis risk. These findings emphasize the importance of addressing socioeconomic and access-to-care disparities in cancer populations. Comprehensive treatment—encompassing surgery, radiation, and chemotherapy—was shown to reduce sepsis mortality, particularly in patients with high-risk cancers such as those of the liver, lung, and pancreas. Observed differences across cancer types and between sexes further highlight the necessity of individualized, risk-based management strategies. While both logistic and Cox regression models were applied, we observed some discordance in effect estimates, likely due to differences in time-to-event censoring and the dynamic risk period post-diagnosis. As such, HRs may better capture the time-dependent nature of sepsis risk in oncology settings.

Our study provides valuable insights into sepsis-specific mortality among cancer patients, emphasizing the importance of gender-stratified analysis to reveal differences often overlooked in prior research. However, there are several limitations: (1) potential biases and misclassification of sepsis-related deaths in the SEER database, (2) lack of detailed data on comorbidities and variations in clinical management, and (3) exclusion of non-fatal sepsis episodes and patients identified only through death certificates or autopsy, which may limit comprehensiveness. Additionally, the SEER database treatment variables include a proportion of “no/unknown.” These categories may represent under-ascertainment of treatment rather than true absence of therapy, potentially leading to misclassification bias. Future prospective, multi-center studies are warranted to validate these findings and guide the development of evidence-based, gender-sensitive interventions to mitigate sepsis mortality in oncology populations.

Our findings carry several clinical implications. First, males and older adults with digestive system cancers (particularly liver and pancreatic) should be prioritized for early sepsis surveillance and prophylactic strategies following diagnosis. Second, the observed sex-specific effects of chemotherapy and radiation underscore the need for personalized supportive care protocols, potentially including earlier use of growth factors and antibiotic prophylaxis in high-risk male patients. Third, the racial and socioeconomic disparities revealed in our analysis highlight the importance of equitable access to infection management resources in oncology. Development of clinical guidelines that incorporate sepsis risk stratification by sex and cancer type may improve outcomes. Future interventional studies are needed to test preventive strategies such as prophylactic antibiotics and tailored supportive care.

## Conclusion

Among cancer patients, males have a significantly higher risk of sepsis-related mortality compared to females. Additionally, notable variations exist based on cancer type, age, and demographic factors. These findings underscore the importance of gender-stratified management strategies in addressing sepsis risks among cancer patients. Tailored interventions that consider gender-specific susceptibilities and treatment responses are crucial for reducing sepsis mortality and improving overall survival outcomes.

## Supplementary Material

pkaf109_Supplementary_Data

## Data Availability

The data used in this study are publicly available through the SEER database (https://seer.cancer.gov/). Researchers may access the data via SEER*Stat software after signing a data use agreement with the National Cancer Institute.
