# Supplementary material for: Gender-stratified analysis of sepsis mortality in cancer: a 45-year population-based cohort study
Source: JNCI Cancer Spectr. 2025 Nov 10;10(2):pkaf109. doi: 10.1093/jncics/pkaf109 (PMC12962810; doi:10.1093/jncics/pkaf109)
Supplement: pkaf109_Supplementary_Data [file pkaf109_supplementary_data.zip › Supplementary Material.pdf]

Supplementary Material

Table S1

Septicemia SMRs and AERs by patients key charateristics for male cancer patients

Charateristics	Patients with cacer	Death of Septicemia,n(%)	Proportion of Septicemia in all cause death(%)	Mortality of Septicemia(per 100000)	person-years	SMR(95%CI)	AER(95%CI)
Total							
Age at Diagnosis							
00-19	23139(1.47)	21(0.33)	0.33	0.68	307398.54	8.49#(5.25, 12.98)	0.6(0.29, 0.91)
20-29	29062(1.85)	37(0.58)	0.41	0.99	373108.34	3.72#(2.62, 5.13)	0.73(0.37, 1.09)
30-39	54916(3.49)	84(1.31)	0.37	1.47	571392.55	2.72#(2.17, 3.37)	0.93(0.56, 1.3)
40-49	108801(6.92)	249(3.89)	0.45	2.81	886795.67	2.51#(2.21, 2.84)	1.69(1.28, 2.1)
50-59	280444(17.83)	821(12.81)	0.54	4.21	1952000.	1.82#(1.7, 1.95)	1.9(1.54, 2.26)
60-69	455642(28.97)	1817(28.36)	0.63	6.54	962779502.	1.39#(1.33, 1.45)	1.83(1.44, 2.22)
70-79	408029(25.94)	2167(33.82)	0.67	11.	391936552.	1.25#(1.2, 1.3)	2.24(1.61, 2.87)
80+	212973(13.54)	1212(18.91)	0.63	1921.	32	1.34#(1.27, 1.42)	5.51(3.91, 7.11)
Race				51	563469.84		
White	1319602(83.89)	5143(80.26)	0.58	5.54	9276889.34	1.34#(1.3, 1.37)	1.61(1.38, 1.84)
Black	122869(7.81)	735(11.47)	0.94	11.6	633534.75	1.56#(1.45, 1.68)	4.18(3.11, 5.25)
Other (American Indian/AK Native, Asian/Pacific Islander)	130535(8.3)	530(8.27)	0.65	7.77	681890.5	2.42#(2.22, 2.63)	4.56(3.77, 5.35)
Marital status at diagnosis							
Single	220975(14.05)	751(11.72)	0.58	4.83	1555418.	2.35#(2.18, 2.52)	3.07(2.61, 3.53)
Married	1001790(63.69)	4042(63.08)	0.6	6.56	916161689.	1.24#(1.2, 1.28)	1.25(0.98, 1.52)
Unmarried or Domestic Partner	2391(0.15)	4(0.06)	0.55	6.14	58	2.66(0.73, 6.82)	4.14(-3.47, 11.75)
Divorced Separated Widowed	238329(15.15)	1156(18.04)	0.61	4.51	[illegible]	1.93#(1.82, 2.04)	5.93(5.06, 6.8)
Unknown	109521(6.96)	455(7.1)	0.87	7.99	569137.7	1.26#(1.14, 1.38)	1.3(0.51, 2.09)
Year of diagnosis							
1975-1979	109299(6.95)	353(5.51)	0.34	5.83	605101.16	1.58#(1.42, 1.75)	2.14(1.36, 2.92)
1980-1989	265812(16.9)	1288(20.1)	0.52	7.82	1647689.	1.62#(1.53, 1.71)	2.98(2.44, 3.52)
1990-1999	339075(21.56)	1818(28.37)	0.64	6.81	192668546.	1.19#(1.14, 1.25)	1.1(0.68, 1.52)
2000-2009	395848(25.17)	1845(28.79)	0.76	6.22	832967812.	1.29#(1.23, 1.35)	1.4(1.02, 1.78)
2010-2019	462972(29.43)	1104(17.23)	0.67	7.45	751481070.	1.96#(1.85, 2.08)	3.65(3.11, 4.19)
Grade					67		
Grade I	114196(8.09)	707(11.71)	0.93	7.31	966960.87	1.25#(1.16, 1.34)	1.45(0.73, 2.17)
Grade II	381645(27.04)	1966(32.58)	0.83	6.11	3220170.	1.04(0.99, 1.09)	0.23(-0.15, 0.61)
Grade III	301408(21.35)	1156(19.15)	0.54	7.02	211645995.	1.29#(1.22, 1.37)	1.57(1.03, 2.11)
Grade IV	69654(4.93)	205(3.4)	0.35	9.25	22	2.29#(1.98, 2.62)	5.21(3.69, 6.73)
Unknown	544750(38.59)	2001(33.16)	0.48	6.99	221559.43	2.14#(2.04, 2.23)	3.72(3.35, 4.09)
Stage					2863569.29		
Localized	259155(22.17)	1512(30.13)	1.01	5.89	2565969	1.41#(1.34, 1.48)	1.72(1.33, 2.11)
Regional	197255(16.87)	919(18.31)	0.58	8.64	1063299.95	2.22#(2.08, 2.37)	4.75(4.08, 5.42)
Distant	277432(23.73)	729(14.52)	0.29	11.05	659562.51	3.28#(3.04, 3.53)	7.68(6.76, 8.6)
Localized/regional (Prostate cases)	227922(19.5)	1004(20)	1.21	4.17	2405141.	0.70#(0.66, 0.75)	-1.76(-2.16, -1.36)
Unstaged	207213(17.73)	855(17.04)	0.53	8.18	421045073.	2.64#(2.46, 2.82)	5.08(4.44, 5.72)
Surgery					58		
No	1573006(67.96)	6408(65.47)	0.61	6.84	9370220.61	1.41#(1.38, 1.45)	2(1.78, 2.22)
Yes	741753(32.04)	3380(34.53)	0.79	5.58	6053612.39	1.25#(1.21, 1.3)	1.13(0.88, 1.38)
Radiation recode							
No	1128608(71.75)	4883(76.2)	0.66	7.16	6818087.25	1.46#(1.42, 1.5)	2.25(1.99, 2.51)
Yes	444398(28.25)	1525(23.8)	0.49	5.98	2552133.36	1.28#(1.22, 1.35)	1.32(0.92, 1.72)
Chemotherapy recode							
No	1195157(75.98)	5297(82.66)	0.69	6.75	7851449.43	1.28#(1.24, 1.31)	1.47(1.23, 1.71)
Yes	377849(24.02)	1111(17.34)	0.4	7.32	1518771.19	2.82#(2.65, 2.99)	4.72(4.22, 5.22)

Table S2

Septicemia SMRs and AERs by patients key charateristics for female cancer patients

Charateristics	Patients with cacer	Death of Septicemia,n(%)	Proportion of Septicemia in all cause death(%)	Mortality of Septicemia(per 100000)	person-years	SMR(95%CI)	AER(95%CI)
Total							
Age at Diagnosis							
00-19	19703(1.32)	25(0.45)	0.56	0.91	275518.04	11.79#(7.63, 17.4)	0.83(0.46, 1.2)
20-29	32247(2.16)	40(0.72)	0.63	0.85	468740.46	3.47#(2.48, 4.73)	0.61(0.31, 0.91)
30-39	82792(5.54)	105(1.9)	0.47	0.99	1055509.61	1.97#(1.61, 2.38)	0.49(0.26, 0.72)
40-49	174051(11.64)	347(6.29)	0.58	1.79	1933682.25	1.74#(1.56, 1.93)	0.76(0.52, 1)
50-59	275067(18.4)	822(14.9)	0.64	3.36	2447501.86	1.59#(1.48, 1.7)	1.25(0.96, 1.54)
60-69	339330(22.7)	1391(25.21)	0.66	5.94	2343064.98	1.46#(1.39, 1.54)	1.88(1.48, 2.28)
70-79	322652(21.59)	1590(28.81)	0.64	9.77	1627763.89	1.27#(1.2, 1.33)	2.05(1.41, 2.69)
80+	248849(16.65)	1198(21.71)	0.54	16.95	706705.03	1.21#(1.14, 1.28)	2.9(1.6, 4.2)
Race							
White	1250120(83.64)	4421(80.12)	0.57	4.77	9276889.34	1.26#(1.23, 1.3)	1(0.81, 1.19)
Black	106850(7.15)	638(11.56)	1	10.26	621972.44	2.04#(1.89, 2.21)	5.23(4.26, 6.2)
Other (American Indian/AK Native, Asian/Pacific Islander)	137721(9.21)	459(8.32)	0.69	4.78	959624.34	2.62#(2.38, 2.87)	2.96(2.45, 3.47)
Marital status at diagnosis							
Single	198748(13.3)	673(12.2)	0.72	4.33	1555418.91	2.14#(1.98, 2.31)	2.31(1.91, 2.71)
Married	712264(47.65)	2258(40.92)	0.59	3.66	6161689.58	1.19#(1.14, 1.24)	0.58(0.37, 0.79)
Unmarried or Domestic Partner	2332(0.16)	2(0.04)	0.42	3.07	6517.78	2.18(0.26, 7.88)	1.66(-3.48, 6.8)
Divorced Separated Widowed	501038(33.52)	2281(41.34)	0.58	8.89	2565722.14	1.45#(1.39, 1.51)	2.76(2.29, 3.23)
Unknown	80309(5.37)	304(5.51)	0.8	5.34	569137.7	1.53#(1.36, 1.71)	1.84(1.07, 2.61)
Year of diagnosis							
1975-1979	106618(7.13)	396(7.18)	0.41	3.76	1053085.01	1.08(0.97, 1.19)	0.26(-0.25, 0.77)
1980-1989	253323(16.95)	1254(22.73)	0.57	5.2	2412581.1	1.29#(1.22, 1.36)	1.16(0.78, 1.54)
1990-1999	307460(20.57)	1469(26.62)	0.63	5.02	2925709.3	1.24#(1.18, 1.31)	0.98(0.64, 1.32)
2000-2009	370962(24.82)	1497(27.13)	0.71	5.1	2937169.87	1.46#(1.39, 1.54)	1.62(1.29, 1.95)
2010-2019	456328(30.53)	902(16.35)	0.64	5.9	1529940.84	2.06#(1.93, 2.2)	3.04(2.57, 3.51)
Grade							
Grade I	150489(11.16)	671(12.79)	1.06	4.19	1601358.83	1.05(0.97, 1.13)	0.2(-0.24, 0.64)
Grade II	307134(22.77)	1409(26.86)	0.82	5.26	2679956.57	1.21#(1.15, 1.28)	0.93(0.56, 1.3)
Grade III	258789(19.19)	922(17.58)	0.52	5.41	1703061.58	1.52#(1.42, 1.62)	1.85(1.4, 2.3)
Grade IV	59202(4.39)	179(3.41)	0.37	6.94	257796.85	2.03#(1.74, 2.35)	3.52(2.28, 4.76)
Unknown	572977(42.49)	2065(39.36)	0.51	4.88	4228520.59	1.52#(1.46, 1.59)	1.68(1.41, 1.95)
Stage							
Localized	511503(40.41)	2397(46.03)	1	3.8	6308978.82	0.98(0.94, 1.02)	-0.09(-0.31, 0.13)
Regional	298131(23.55)	1327(25.48)	0.65	5.47	2426543.24	1.54#(1.46, 1.62)	1.91(1.53, 2.29)
Distant	272152(21.5)	755(14.5)	0.31	10.16	743091.13	3.27#(3.04, 3.51)	7.05(6.22, 7.88)
Unstaged	184122(14.54)	728(13.98)	0.51	7.41	982856.82	2.21#(2.06, 2.38)	4.06(3.41, 4.71)
Surgery							
No	1494691(60.15)	5518(58.64)	0.61	5.08	10858486.12	1.38#(1.35, 1.42)	1.41(1.23, 1.59)
Yes	990381(39.85)	3892(41.36)	0.79	4.13	9417435.06	1.13#(1.09, 1.16)	0.46(0.28, 0.64)
Radiation recode							
No	1042802(69.77)	4133(74.9)	0.62	5.5	7508959.86	1.39#(1.35, 1.44)	1.55(1.33, 1.77)
Yes	451889(30.23)	1385(25.1)	0.58	4.13	3349526.27	1.36#(1.29, 1.43)	1.09(0.8, 1.38)
Chemotherapy recodeye							
No	1035070(69.25)	4299(77.91)	0.68	5.31	8089548.4	1.28#(1.24, 1.32)	1.16(0.95, 1.37)
Yes	459621(30.75)	1219(22.09)	0.45	4.4	2768937.72	1.95#(1.85, 2.07)	2.15(1.85, 2.45)

Table S3

SMRs and AERs for Male

Site recode ICD-O-3/WHO 2008	Observed	Expected	SMR(95%CI)	AER(95%CI)
All Sites	6408	4536.03	1.41#(1.38, 1.45)	2(1.78, 2.22)
Oral Cavity and Pharynx	208	98.91	2.10#(1.83, 2.41)	3.64(2.49, 4.79)
Lip	48	31.69	1.51#(1.12, 2.01)	2.6(-0.19, 5.39)
Tongue	41	19.13	2.14#(1.54, 2.91)	3.26(0.99, 5.53)
Floor of Mouth	17	4.97	3.42#(1.99, 5.48)	7.36(1.74, 12.98)
Gum and Other Mouth	24	10.69	2.24#(1.44, 3.34)	4.75(0.63, 8.87)
Nasopharynx	16	3.82	4.18#(2.39, 6.79)	4.75(1.35, 8.15)
Tonsil	24	10.56	2.27#(1.46, 3.38)	2.86(0.41, 5.31)
Hypopharynx	19	3.83	4.96#(2.99, 7.75)	13.67(5.24, 22.1)
Digestive System	1396	635.52	2.20#(2.08, 2.31)	6.02(5.32, 6.72)
Esophagus	89	16.06	5.54#(4.45, 6.82)	17.52(12.7, 22.34)
Stomach	120	39.65	3.03#(2.51, 3.62)	9.41(6.51, 12.31)
Small Intestine	28	11.58	2.42#(1.61, 3.5)	5.23(1.31, 9.15)
Colon and Rectum	855	522.41	1.64#(1.53, 1.75)	3.46(2.7, 4.22)
Colon excluding Rectum	592	367.34	1.61#(1.48, 1.75)	3.61(2.63, 4.59)
Cecum	112	76.97	1.46#(1.2, 1.75)	3.06(0.7, 5.42)
Ascending Colon	96	56.39	1.70#(1.38, 2.08)	4.7(1.83, 7.57)
Hepatic Flexure	36	17.53	2.05#(1.44, 2.84)	6.75(1.51, 11.99)
Transverse Colon	50	29.3	1.71#(1.27, 2.25)	4.33(0.68, 7.98)
Splenic Flexure	30	12.85	2.33#(1.57, 3.33)	7.7(1.94, 13.46)
Descending Colon	43	24.45	1.76#(1.27, 2.37)	4.16(0.55, 7.77)
Sigmoid Colon	196	137.94	1.42#(1.23, 1.63)	2.32(0.89, 3.75)
Large Intestine, NOS	22	8.22	2.68#(1.68, 4.05)	8.75(1.91, 15.59)
Rectum and Rectosigmoid Junctio	263	155.07	1.70#(1.5, 1.91)	3.17(1.99, 4.35)
Rectosigmoid Junction	84	47.05	1.79#(1.42, 2.21)	3.86(1.52, 6.2)
Rectum	179	108.02	1.66#(1.42, 1.92)	2.9(1.54, 4.26)
Anus, Anal Canal and Anorectum	18	7.03	2.56#(1.52, 4.05)	4.94(0.53, 9.35)
Liver and Intrahepatic Bile Duct	134	13.71	9.77#(8.19, 11.58)	22.98(18.43, 27.53)
Liver	120	12.54	9.57#(7.94, 11.45)	21.9(17.31, 26.49)
Intrahepatic Bile Duct	14	1.17	11.92#(6.52, 20)	39.24(15.93, 62.55)
Other Biliary	32	5.63	5.69#(3.89, 8.03)	21.3(11.6, 31)
Pancreas	102	14.69	6.94#(5.66, 8.43)	21.59(16.36, 26.82)
Respiratory System	675	209.22	3.23#(2.99, 3.48)	9.14(8, 10.28)
Larynx	90	53.52	1.68#(1.35, 2.07)	3.05(1.09, 5.01)
Lung and Bronchus	571	149.94	3.81#(3.5, 4.13)	11.51(10.07, 12.95)
Bones and Joints	12	3.5	3.43#(1.77, 5.98)	2.29(0.21, 4.37)
Soft Tissue including Heart	19	20.99	0.91(0.54, 1.41)	-0.23(-1.63, 1.17)
Skin excluding Basal and Squamou	178	198.21	0.9(0.77, 1.04)	-0.33(-0.94, 0.28)
Melanoma of the Skin	150	179.2	0.84#(0.71, 0.98)	-0.51(-1.14, 0.12)
Other Non-Epithelial Skin	28	19.01	1.47(0.98, 2.13)	1.74(-0.86, 4.34)
Breast	16	11.08	1.44(0.83, 2.35)	2.39(-2.56, 7.34)
Male Genital System	2141	2506.94	0.85#(0.82, 0.89)	-0.88(-1.2, -0.56)
Prostate	2079	2474.29	0.84#(0.8, 0.88)	-1.04(-1.39, -0.69)
Testis	38	22.4	1.70#(1.2, 2.33)	0.45(0.01, 0.89)
Penis	20	7.56	2.65#(1.62, 4.09)	8.5(1.47, 15.53)
Urinary System	726	474.03	1.53#(1.42, 1.65)	2.77(2.02, 3.52)
Urinary Bladder	531	366.55	1.45#(1.33, 1.58)	2.68(1.72, 3.64)
Kidney and Renal Pelvis	185	101.42	1.82#(1.57, 2.11)	2.92(1.76, 4.08)
Brain and Other Nervous System	48	9.73	4.94#(3.64, 6.54)	2.81(1.72, 3.9)
Brain	40	7.88	5.08#(3.63, 6.92)	2.69(1.56, 3.82)
Endocrine System	49	33.53	1.46#(1.08, 1.93)	0.79(-0.12, 1.7)
Thyroid	31	31.1	1(0.68, 1.41)	-0.01(-0.89, 0.87)
Other Endocrine including Thymu:	18	2.43	7.41#(4.39, 11.71)	7.23(3.12, 11.34)
Lymphoma	318	143.89	2.21#(1.97, 2.47)	3.14(2.38, 3.9)
Hodgkin Lymphoma	45	12.37	3.64#(2.65, 4.87)	2.16(1.18, 3.14)
Hodgkin - Nodal	44	12.06	3.65#(2.65, 4.9)	2.14(1.16, 3.12)
Non-Hodgkin Lymphoma	273	131.52	2.08#(1.84, 2.34)	3.51(2.53, 4.49)
NHL - Nodal	209	84.48	2.47#(2.15, 2.83)	4.59(3.35, 5.83)
NHL - Extranodal	64	47.05	1.36#(1.05, 1.74)	1.29(-0.28, 2.86)
Myeloma	101	34.4	2.94#(2.39, 3.57)	8.14(5.35, 10.93)
Leukemia	197	85.92	2.29#(1.98, 2.64)	3.86(2.72, 5)
Lymphocytic Leukemia	111	69.64	1.59#(1.31, 1.92)	1.96(0.71, 3.21)
Acute Lymphocytic Leukemia	15	1.33	11.26#(6.3, 18.56)	1.9(0.8, 3)
Chronic Lymphocytic Leukemia	86	61.83	1.39#(1.11, 1.72)	2.04(0.02, 4.06)
Other Lymphocytic Leukemia	10	6.47	1.55(0.74, 2.84)	1.75(-2.18, 5.68)
Myeloid and Monocytic Leukemia	71	14.34	4.95#(3.87, 6.24)	8.01(5.45, 10.57)
Acute Myeloid Leukemia	36	5.01	7.19#(5.04, 9.95)	9.38(5.59, 13.17)
Chronic Myeloid Leukemia	29	8.57	3.38#(2.27, 4.86)	6(2.47, 9.53)
Other Leukemia	15	1.94	7.72#(4.32, 12.74)	19.17(7.34, 31)
Kaposi Sarcoma	43	6.66	6.46#(4.68, 8.7)	7.79(4.83, 10.75)
Miscellaneous	265	52.19	5.08#(4.48, 5.73)	18.95(15.84, 22.06)

SMRs and AERs for Female

Site recode ICD-O-3/WHO 2008	Observed	Expected	SMR(95%CI)	AER(95%CI)
All Sites	5518	3984.86	1.38#(1.35, 1.42)	1.41(1.23, 1.59)
Oral Cavity and Pharynx	100	52.74	1.90#(1.54, 2.31)	3.03(1.48, 4.58)
Tongue	19	11.31	1.68#(1.01, 2.62)	2.32(-0.93, 5.57)
Floor of Mouth	10	3.39	2.95#(1.42, 5.43)	6.88(-0.58, 14.34)
Gum and Other Mouth	27	13.21	2.04#(1.35, 2.97)	4.25(0.42, 8.08)
Tonsil	14	3.2	4.37#(2.39, 7.33)	8.76(2.17, 15.35)
Digestive System	1235	731.73	1.69#(1.59, 1.78)	3.88(3.21, 4.55)
Esophagus	30	5.88	5.10#(3.44, 7.28)	18.56(9.53, 27.59)
Stomach	88	32.23	2.73#(2.19, 3.36)	8.55(5.26, 11.84)
Small Intestine	20	11.51	1.74#(1.06, 2.68)	2.87(-0.85, 6.59)
Colon and Rectum	854	628.13	1.36#(1.27, 1.45)	2.16(1.44, 2.88)
Colon excluding Rectum	650	484.82	1.34#(1.24, 1.45)	2.2(1.32, 3.08)
Cecum	169	127.18	1.33#(1.14, 1.54)	2.55(0.5, 4.6)
Ascending Colon	113	87.77	1.29#(1.06, 1.55)	2.15(-0.21, 4.51)
Hepatic Flexure	35	23.94	1.46#(1.02, 2.03)	3.38(-1.21, 7.97)
Transverse Colon	63	44.08	1.43#(1.1, 1.83)	2.93(-0.21, 6.07)
Splenic Flexure	19	13.74	1.38(0.83, 2.16)	2.31(-2.62, 7.24)
Descending Colon	41	28.3	1.45#(1.04, 1.97)	2.52(-0.72, 5.76)
Sigmoid Colon	186	145.25	1.28#(1.1, 1.48)	1.55(0.19, 2.91)
Large Intestine, NOS	19	10.65	1.78#(1.07, 2.79)	5(-1.38, 11.38)
Rectum and Rectosigmoid Junction	204	143.32	1.42#(1.23, 1.63)	2.06(0.82, 3.3)
Rectosigmoid Junction	57	48.97	1.16(0.88, 1.51)	0.86(-1.29, 3.01)
Rectum	147	94.34	1.56#(1.32, 1.83)	2.63(1.11, 4.15)
Anus, Anal Canal and Anorectum	23	14.28	1.61#(1.02, 2.42)	2.36(-0.88, 5.6)
Liver and Intrahepatic Bile Duct	56	6.21	9.02#(6.81, 11.71)	21.68(14.95, 28.41)
Liver	45	5.04	8.92#(6.51, 11.94)	20.26(13.24, 27.28)
Intrahepatic Bile Duct	11	1.17	9.42#(4.7, 16.86)	30.38(9.29, 51.47)
Gallbladder	20	6.1	3.28#(2, 5.07)	11.67(3.27, 20.07)
Other Biliary	24	4.94	4.86#(3.11, 7.23)	18.4(8.23, 28.57)
Pancreas	101	16.4	6.16#(5.02, 7.48)	19.87(14.89, 24.85)
Respiratory System	406	150.17	2.70#(2.45, 2.98)	6.57(5.38, 7.76)
Larynx	21	9.55	2.20#(1.36, 3.36)	4.13(0.23, 8.03)
Lung and Bronchus	377	136.14	2.77#(2.5, 3.06)	6.97(5.69, 8.25)
Soft Tissue including Heart	27	18.34	1.47(0.97, 2.14)	1.12(-0.59, 2.83)
Skin excluding Basal and Squamous	134	162.99	0.82#(0.69, 0.97)	-0.43(-0.93, 0.07)
Melanoma of the Skin	112	147	0.76#(0.63, 0.92)	-0.56(-1.06, -0.06)
Other Non-Epithelial Skin	22	16	1.38(0.86, 2.08)	1.21(-1.22, 3.64)
Breast	1569	1625.2	0.97(0.92, 1.01)	-0.13(-0.39, 0.13)
Female Genital System	907	618.88	1.47#(1.37, 1.56)	1.58(1.16, 2)
Cervix Uteri	143	62.82	2.28#(1.92, 2.68)	2.11(1.37, 2.85)
Corpus and Uterus, NOS	546	438.51	1.25#(1.14, 1.35)	1.03(0.44, 1.62)
Corpus Uteri	541	436.36	1.24#(1.14, 1.35)	1.01(0.42, 1.6)
Ovary	140	79.47	1.76#(1.48, 2.08)	1.96(1.02, 2.9)
Vagina	13	4.74	2.74#(1.46, 4.69)	6.99(0.01, 13.97)
Vulva	52	26.96	1.93#(1.44, 2.53)	4.5(1.37, 7.63)
Other Female Genital Organs	13	6.37	2.04#(1.09, 3.49)	2.5(-0.76, 5.76)
Urinary System	325	207.85	1.56#(1.4, 1.74)	2.73(1.68, 3.78)
Urinary Bladder	198	133.35	1.48#(1.29, 1.71)	2.86(1.28, 4.44)
Kidney and Renal Pelvis	123	70.22	1.75#(1.46, 2.09)	2.68(1.3, 4.06)
Eye and Orbit	15	8.35	1.80#(1.01, 2.96)	2.12(-0.9, 5.14)
Brain and Other Nervous System	43	8.55	5.03#(3.64, 6.78)	3(1.77, 4.23)
Brain	36	6.44	5.59#(3.92, 7.74)	2.98(1.69, 4.27)
Endocrine System	67	86.75	0.77#(0.6, 0.98)	-0.3(-0.67, 0.07)
Thyroid	59	84	0.70#(0.53, 0.91)	-0.39(-0.76, -0.02)
Lymphoma	239	152.69	1.57#(1.37, 1.78)	1.75(0.97, 2.53)
Hodgkin Lymphoma	27	9.53	2.83#(1.87, 4.12)	1.41(0.45, 2.37)
Hodgkin - Nodal	27	9.23	2.92#(1.93, 4.26)	1.47(0.5, 2.44)
Non-Hodgkin Lymphoma	212	143.16	1.48#(1.29, 1.69)	1.86(0.86, 2.86)
NHL - Nodal	143	91.84	1.56#(1.31, 1.83)	2.07(0.85, 3.29)
NHL - Extranodal	69	51.31	1.34#(1.05, 1.7)	1.42(-0.31, 3.15)
Myeloma	76	28.98	2.62#(2.07, 3.28)	6.67(3.82, 9.52)
Leukemia	136	70.37	1.93#(1.62, 2.29)	2.93(1.67, 4.19)
Lymphocytic Leukemia	79	56.95	1.39#(1.1, 1.73)	1.4(-0.05, 2.85)
Acute Lymphocytic Leukemia	15	1.1	13.59#(7.61, 22.41)	2.39(1.04, 3.74)
Chronic Lymphocytic Leukemia	63	53.32	1.18(0.91, 1.51)	1.05(-1.23, 3.33)
Myeloid and Monocytic Leukemia	48	11.51	4.17#(3.07, 5.53)	6.06(3.55, 8.57)
Acute Myeloid Leukemia	26	4.23	6.15#(4.02, 9.01)	6.91(3.49, 10.33)
Chronic Myeloid Leukemia	19	6.64	2.86#(1.72, 4.47)	4.89(0.96, 8.82)
Miscellaneous	223	54.34	4.10#(3.58, 4.68)	14.94(12.05, 17.83)

Table S4

Number of cases died of septicemia in male cancer patients

Age	Colon and Rectum	y and Renal	Leukemia	Liver	g and Bronc	Lymphoma	avity and Pl	Other	Pancreas	Prostate	n excluding	Stomach	Thyroid	rinary Bladder
0-19	0	2	7	0	0	1	0	11	0	0	0	0	0	0
20-29	0	0	1	0	0	15	1	19	0	0	0	0	1	0
30-39	2	2	14	2	3	11	2	38	1	0	3	0	3	3
40-49	22	14	13	5	11	31	10	82	5	13	11	9	7	16
50-59	100	40	33	36	105	68	40	146	21	142	23	19	7	41
60-69	243	53	49	47	174	78	66	212	30	635	44	29	7	150
70-79	286	55	49	21	201	70	69	239	32	877	57	37	5	169
80+	202	19	31	9	77	44	20	166	13	412	40	26	1	152

Percentage of cases died of septicemia in male cancer patients

0-19	0	9.52381	33.33333	0	0	4.761905	0	52.38095	0	0	0	0	0	0
20-29	0	0	2.702703	0	0	40.54054	2.702703	51.35135	0	0	0	0	2.702703	0
30-39	2.380952381	2.380952	16.66667	2.380952	3.571429	13.09524	2.380952	45.2381	1.190476	0	3.571429	0	3.571429	3.571429
40-49	8.835341365	5.62249	5.220884	2.008032	4.417671	12.4498	4.016064	32.93173	2.008032	5.220884	4.417671	3.614458	2.811245	6.425703
50-59	12.18026797	4.872107	4.019488	4.384896	12.78928	8.282582	4.872107	17.78319	2.557856	17.29598	2.801462	2.314251	0.852619	4.99391
60-69	13.3736929	2.916896	2.696753	2.586681	9.576225	4.29279	3.632361	11.66758	1.651073	34.94772	2.421574	1.596037	0.38525	8.255366
70-79	13.19796954	2.538071	2.261191	0.969082	9.275496	3.230272	3.184126	11.02907	1.476696	40.4707	2.630365	1.70743	0.230734	7.7988
80+	16.66666667	1.567657	2.557756	0.742574	6.353135	3.630363	1.650165	13.69637	1.072607	33.9934	3.30033	2.145215	0.082508	12.54125

Table S5

Number of cases died of septicemia in female cancer patients

Age	Breast	Cervix Uteri	on and Rec	Corpus Uter/	and Renal	Leukemia	Liver	g and Bronc	Lymphoma	avity and Pl	Other	Ovary	Pancreas	n excluding	Stomach	Thyroid	rinary Bladder
0-19	0	0	0	0	1	10	0	0	3	0	11	0	0	0	0	0	0
20-29	3	5	1	0	0	7	0	0	8	1	10	3	1	1	0	0	0
30-39	23	14	3	9	2	4	0	0	13	1	16	8	0	7	0	5	0
40-49	110	27	24	34	7	7	2	20	10	12	40	11	0	14	6	17	6
50-59	240	42	80	140	14	17	9	53	24	16	80	25	19	21	9	14	19
60-69	433	26	178	177	38	25	12	114	47	30	153	34	24	27	14	9	50
70-79	436	24	292	127	37	44	11	130	76	17	181	39	28	35	38	9	66
80+	324	5	276	54	24	22	11	60	58	23	180	20	29	29	21	5	57

Percentage of cases died of septicemia in female cancer patients

0-19	0	0	0	0	4	40	0	0	12	0	44	0	0	0	0	0	0
20-29	7.5	12.5	2.5	0	0	17.5	0	0	20	2.5	25	7.5	2.5	2.5	0	0	0
30-39	21.90476	13.33333	2.857143	8.571429	1.904762	3.809524	0	0	12.38095	0.952381	15.2381	7.619048	0	6.666667	0	4.761905	0
40-49	31.70029	7.78098	6.916427	9.798271	2.017291	2.017291	0.576369	5.763689	2.881844	3.458213	11.52738	3.170029	0	4.034582	1.729107	4.899135	1.729107
50-59	29.19708	5.109489	9.73236	17.03163	1.703163	2.068127	1.094891	6.447689	2.919708	1.946472	9.73236	3.041363	2.311436	2.554745	1.094891	1.703163	2.311436
60-69	31.12868	1.869159	12.79655	12.72466	2.731848	1.797268	0.862689	8.195543	3.378864	2.156722	10.99928	2.444285	1.725377	1.94105	1.00647	0.647017	3.594536
70-79	27.42138	1.509434	18.36478	7.987421	2.327044	2.767296	0.691824	8.176101	4.779874	1.069182	11.38365	2.45283	1.761006	2.201258	2.389937	0.566038	4.150943
80+	27.04508	0.417362	23.0384	4.507513	2.003339	1.836394	0.918197	5.008347	4.841402	1.919866	15.02504	1.669449	2.420701	2.420701	1.752922	0.417362	4.75793

Table S6

Septicemia SMRs and AERs by cancer type, time since diagnosis, and duration for male patients

Sex	Latency	Selected Events	Site recode ICD-O-3/WHO 2008	Observed	Expected	Persons	Person Years at Risk	SMR(95%CI)	AER(95%CI)
Male	0-11 months	Septicemia	All Sites	1756	440.54	1573006	1250792.12	3.99#(3.8, 4.18)	10.52(9.79, 11.25)
Male	0-11 months	Septicemia	Oral Cavity and Pharynx	56	10.82	49561	43146.04	5.18#(3.91, 6.72)	10.47(6.76, 14.18)
Male	0-11 months	Septicemia	Stomach	60	8.44	36651	22982.79	7.11#(5.42, 9.15)	22.43(15.38, 29.48)
Male	0-11 months	Septicemia	Colon and Rectum	250	48.88	157916	131737.37	5.11#(4.5, 5.79)	15.27(12.7, 17.84)
Male	0-11 months	Septicemia	Liver	77	4.2	29878	15914.85	18.35#(14.48, 22.94)	45.75(34.68, 56.82)
Male	0-11 months	Septicemia	Pancreas	74	7.14	43939	20258.58	10.37#(8.14, 13.02)	33.01(24.31, 41.71)
Male	0-11 months	Septicemia	Lung and Bronchus	341	46.39	232169	131966.12	7.35#(6.59, 8.17)	22.32(19.4, 25.24)
Male	0-11 months	Septicemia	Skin excluding Basal and Squamous	18	17.36	65096	60388.7	1.04(0.61, 1.64)	0.11(-1.82, 2.04)
Male	0-11 months	Septicemia	Prostate	178	173.68	438459	415472.08	1.02(0.88, 1.19)	0.1(-0.78, 0.98)
Male	0-11 months	Septicemia	Urinary Bladder	111	35.08	81276	72728.17	3.16#(2.6, 3.81)	10.44(7.18, 13.7)
Male	0-11 months	Septicemia	Kidney and Renal Pelvis	37	10.76	47509	39312.54	3.44#(2.42, 4.74)	6.68(3.24, 10.12)
Male	0-11 months	Septicemia	Thyroid	3	1.88	16195	15065.89	1.6(0.33, 4.66)	0.74(-2.13, 3.61)
Male	0-11 months	Septicemia	Lymphoma	94	16.77	79456	64949.9	5.61#(4.53, 6.86)	11.89(8.72, 15.06)
Male	0-11 months	Septicemia	Leukemia	65	12.19	53041	40198.69	5.33#(4.12, 6.8)	13.14(8.86, 17.42)
Male	12-59 months	Septicemia	All Sites	1655	1247.26	1076602	3149211.76	1.33#(1.26, 1.39)	1.29(0.95, 1.63)
Male	12-59 months	Septicemia	Oral Cavity and Pharynx	59	27.62	37477	102699.6	2.14#(1.63, 2.76)	3.06(1.28, 4.84)
Male	12-59 months	Septicemia	Stomach	25	12.55	15731	30868.56	1.99#(1.29, 2.94)	4.03(0.14, 7.92)
Male	12-59 months	Septicemia	Colon and Rectum	197	140.8	117337	333548.73	1.40#(1.21, 1.61)	1.68(0.6, 2.76)
Male	12-59 months	Septicemia	Liver	35	5.4	10682	20866.1	6.48#(4.52, 9.02)	14.19(8.22, 20.16)
Male	12-59 months	Septicemia	Pancreas	18	4.58	10376	13543.1	3.93#(2.33, 6.21)	9.91(3.04, 16.78)
Male	12-59 months	Septicemia	Lung and Bronchus	136	51.31	80690	133450.41	2.65#(2.22, 3.14)	6.35(4.34, 8.36)
Male	12-59 months	Septicemia	Skin excluding Basal and Squamous	52	53.07	57001	183965.31	0.98(0.73, 1.28)	-0.06(-1.15, 1.03)
Male	12-59 months	Septicemia	Prostate	507	641.67	397396	1322024.06	0.79#(0.72, 0.86)	-1.02(-1.52, -0.52)
Male	12-59 months	Septicemia	Urinary Bladder	148	106.02	66443	205472.3	1.40#(1.18, 1.64)	2.04(0.52, 3.56)
Male	12-59 months	Septicemia	Kidney and Renal Pelvis	58	30.16	34448	101710.24	1.92#(1.46, 2.49)	2.74(0.93, 4.55)
Male	12-59 months	Septicemia	Thyroid	7	6.66	14326	49280.4	1.05(0.42, 2.17)	0.07(-1.4, 1.54)
Male	12-59 months	Septicemia	Lymphoma	81	44.6	56913	173847	1.82#(1.44, 2.26)	2.09(0.83, 3.35)
Male	12-59 months	Septicemia	Leukemia	77	30.2	34607	98802.07	2.55#(2.01, 3.19)	4.74(2.69, 6.79)
Male	60-119 months	Septicemia	All Sites	1272	1178.73	610095	2365377.62	1.08#(1.02, 1.14)	0.39(-0.02, 0.8)
Male	60-119 months	Septicemia	Oral Cavity and Pharynx	39	23.3	18969	71621.83	1.67#(1.19, 2.29)	2.19(0.03, 4.35)
Male	60-119 months	Septicemia	Stomach	16	8.39	4562	16202.7	1.91#(1.09, 3.1)	4.7(-1.27, 10.67)
Male	60-119 months	Septicemia	Colon and Rectum	157	125.79	61383	232572.12	1.25#(1.06, 1.46)	1.34(-0.08, 2.76)
Male	60-119 months	Septicemia	Liver	7	1.93	2599	7767.52	3.62#(1.45, 7.45)	6.52(-1.02, 14.06)
Male	60-119 months	Septicemia	Pancreas	7	1.71	1436	4189.4	4.09#(1.64, 8.42)	12.62(-1.18, 26.42)
Male	60-119 months	Septicemia	Lung and Bronchus	61	25.59	16896	55199.66	2.38#(1.82, 3.06)	6.41(3.11, 9.71)
Male	60-119 months	Septicemia	Skin excluding Basal and Squamous	40	44.9	37310	149280.76	0.89(0.64, 1.21)	-0.33(-1.54, 0.88)
Male	60-119 months	Septicemia	Prostate	548	703.37	274370	1088439.54	0.78#(0.72, 0.85)	-1.43(-2.07, -0.79)
Male	60-119 months	Septicemia	Urinary Bladder	123	91.88	40420	155018.78	1.34#(1.11, 1.6)	2.01(0.16, 3.86)
Male	60-119 months	Septicemia	Kidney and Renal Pelvis	42	25.74	19200	71365.13	1.63#(1.18, 2.21)	2.28(0.02, 4.54)
Male	60-119 months	Septicemia	Thyroid	6	6.82	10508	42656.71	0.88(0.32, 1.92)	-0.19(-1.84, 1.46)
Male	60-119 months	Septicemia	Lymphoma	55	35.71	34362	134085.89	1.54#(1.16, 2)	1.44(0.05, 2.83)
Male	60-119 months	Septicemia	Leukemia	34	21.27	18365	67782.74	1.60#(1.11, 2.23)	1.88(-0.27, 4.03)
Male	120-239 months	Septicemia	All Sites	1338	1292.55	356979	2029578.04	1.04(0.98, 1.09)	0.22(-0.28, 0.72)
Male	120-239 months	Septicemia	Oral Cavity and Pharynx	40	25.01	10556	61290.25	1.60#(1.14, 2.18)	2.45(-0.13, 5.03)
Male	120-239 months	Septicemia	Stomach	15	7.76	2289	12212.21	1.93#(1.08, 3.19)	5.93(-1.72, 13.58)
Male	120-239 months	Septicemia	Colon and Rectum	184	147.92	35080	205953.3	1.24#(1.07, 1.44)	1.75(0.02, 3.48)
Male	120-239 months	Septicemia	Liver	1	0.91	896	3803.37	1.1(0.03, 6.12)	0.24(-6.88, 7.36)
Male	120-239 months	Septicemia	Pancreas	2	1.04	465	2034.68	1.93(0.23, 6.96)	4.72(-12.07, 21.51)
Male	120-239 months	Septicemia	Lung and Bronchus	23	20.16	7101	36088.74	1.14(0.72, 1.71)	0.79(-2.78, 4.36)
Male	120-239 months	Septicemia	Skin excluding Basal and Squamous	49	51.93	23739	154827.71	0.94(0.7, 1.25)	-0.19(-1.46, 1.08)
Male	120-239 months	Septicemia	Prostate	699	798.47	164233	856586.05	0.88#(0.81, 0.94)	-1.16(-2.05, -0.27)
Male	120-239 months	Septicemia	Urinary Bladder	115	95.13	23289	136686.2	1.21(1, 1.45)	1.45(-0.63, 3.53)
Male	120-239 months	Septicemia	Kidney and Renal Pelvis	36	25.18	10354	56409.82	1.43#(1, 1.98)	1.92(-0.8, 4.64)
Male	120-239 months	Septicemia	Thyroid	7	8.54	6797	44229.73	0.82(0.33, 1.69)	-0.35(-2.1, 1.4)
Male	120-239 months	Septicemia	Lymphoma	62	33.12	20599	127729.82	1.87#(1.44, 2.4)	2.26(0.76, 3.76)
Male	120-239 months	Septicemia	Leukemia	15	17.25	9807	58233.57	0.87(0.49, 1.43)	-0.39(-2.3, 1.52)
Male	240-359 months	Septicemia	All Sites	318	325.81	94792	478506.64	0.98(0.87, 1.09)	-0.16(-1.2, 0.88)
Male	240-359 months	Septicemia	Oral Cavity and Pharynx	8	9.44	3072	17000.34	0.85(0.37, 1.67)	-0.85(-5.66, 3.96)
Male	240-359 months	Septicemia	Stomach	3	2.13	555	2702.2	1.41(0.29, 4.11)	3.22(-13.2, 19.64)
Male	240-359 months	Septicemia	Colon and Rectum	52	50.2	10098	50772.05	1.04(0.77, 1.36)	0.36(-3.54, 4.26)
Male	240-359 months	Septicemia	Liver	0	0.08	115	570.66	0(0, 45.24)	-1.43(-11.14, 8.28)
Male	240-359 months	Septicemia	Pancreas	1	0.2	70	328.8	5.09(0.13, 28.37)	24.44(-40.77, 89.65)
Male	240-359 months	Septicemia	Lung and Bronchus	8	5.34	1508	7592.12	1.5(0.65, 2.95)	3.5(-5.92, 12.92)
Male	240-359 months	Septicemia	Skin excluding Basal and Squamous	16	22.92	9228	55612.28	0.7(0.4, 1.13)	-1.24(-3.44, 0.96)
Male	240-359 months	Septicemia	Prostate	136	151.05	30933	108502.45	0.9(0.76, 1.07)	-1.39(-4.45, 1.67)
Male	240-359 months	Septicemia	Urinary Bladder	26	31.74	6933	37428.21	0.82(0.54, 1.2)	-1.53(-5.51, 2.45)
Male	240-359 months	Septicemia	Kidney and Renal Pelvis	11	7.88	2604	14123.39	1.4(0.7, 2.5)	2.21(-3.82, 8.24)
Male	240-359 months	Septicemia	Thyroid	6	4.85	2717	17527.26	1.24(0.45, 2.69)	0.66(-3.02, 4.34)
Male	240-359 months	Septicemia	Lymphoma	20	10.65	7229	42530.41	1.88#(1.15, 2.9)	2.2(-0.35, 4.75)
Male	240-359 months	Septicemia	Leukemia	4	4.28	3112	18197.93	0.93(0.25, 2.39)	-0.15(-3.25, 2.95)
Male	360-479 months	Septicemia	All Sites	68	48.22	19190	91083.49	1.41#(1.1, 1.79)	2.17(-0.15, 4.49)
Male	360-479 months	Septicemia	Oral Cavity and Pharynx	6	2.54	782	3733.29	2.36(0.87, 5.15)	9.27(-6.06, 24.6)
Male	360-479 months	Septicemia	Stomach	1	0.36	101	395.15	2.76(0.07, 15.4)	16.15(-41.63, 73.93)
Male	360-479 months	Septicemia	Colon and Rectum	15	8.47	1880	7547.25	1.77(0.99, 2.92)	8.65(-3.92, 21.22)
Male	360-479 months	Septicemia	Liver	0	0.01	26	139.11	0(0, 251.62)	-1.05(-15.14, 13.04)
Male	360-479 months	Septicemia	Pancreas	0	0.03	14	70.08	0(0, 137.51)	-3.83(-52.26, 44.6)
Male	360-479 months	Septicemia	Lung and Bronchus	2	1.05	298	1306.8	1.9(0.23, 6.88)	7.27(-18.91, 33.45)
Male	360-479 months	Septicemia	Skin excluding Basal and Squamous	3	7.45	2814	14108.91	0.4(0.08, 1.18)	-3.15(-7.64, 1.34)
Male	360-479 months	Septicemia	Prostate	11	5.98	1277	3423	1.84(0.92, 3.29)	14.67(-8.89, 38.23)
Male	360-479 months	Septicemia	Urinary Bladder	7	6.38	1555	6904.94	1.1(0.44, 2.26)	0.9(-9.48, 11.28)
Male	360-479 months	Septicemia	Kidney and Renal Pelvis	1	1.62	617	2904.31	0.62(0.02, 3.44)	-2.13(-13.05, 8.79)
Male	360-479 months	Septicemia	Thyroid	2	2.08	989	5568.37	0.96(0.12, 3.47)	-0.15(-7.26, 6.96)
Male	360-479 months	Septicemia	Lymphoma	6	2.84	2142	10557.59	2.11(0.78, 4.6)	2.99(-2.53, 8.51)
Male	360-479 months	Septicemia	Leukemia	2	0.7	923	4552.81	2.87(0.35, 10.36)	2.86(-4.21, 9.93)
Male	480+ months	Septicemia	All Sites	1	2.92	2639	5670.94	0.34(0.01, 1.91)	-3.38(-10.22, 3.46)
Male	480+ months	Septicemia	Oral Cavity and Pharynx	0	0.18	115	248.41	0(0, 20.39)	-7.28(-40.74, 26.18)
Male	480+ months	Septicemia	Stomach	0	0.02	8	17.8	0(0, 238.59)	-8.69(-164.32, 146.94)
Male	480+ months	Septicemia	Colon and Rectum	0	0.36	167	316.05	0(0, 10.36)	-11.27(-48.46, 25.92)
Male	480+ months	Septicemia	Liver	0	0	4	8.42	0(0, 18351.59)	-0.24(-0.24, -0.24)
Male	480+ months	Septicemia	Pancreas	0	0	3	5.34	0(0, 1037.85)	-6.66(-6.66, -6.66)
Male	480+ months	Septicemia	Lung and Bronchus	0	0.09	41	91.09	0(0, 41.09)	-9.86(-74.38, 54.66)
Male	480+ months	Septicemia	Skin excluding Basal and Squamous	0	0.58	429	931.39	0(0, 6.37)	-6.22(-22.24, 9.8)
Male	480+ months	Septicemia	Prostate	0	0.07	21	39.61	0(0, 52.63)	-17.7(-148.5, 113.1)
Male	480+ months	Septicemia	Urinary Bladder	1	0.32	163	340.93	3.17(0.08, 17.66)	20.08(-45.89, 86.05)
Male	480+ months	Septicemia	Kidney and Renal Pelvis	0	0.08	82	163.65	0(0, 44.89)	-5.02(-38.89, 28.85)
Male	480+ months	Septicemia	Thyroid	0	0.27	228	545.04	0(0, 13.74)	-4.93(-23.61, 13.75)
Male	480+ months	Septicemia	Lymphoma	0	0.2	296	649.95	0(0, 18.09)	-3.14(-16.62, 10.34)
Male	480+ months	Septicemia	Leukemia	0	0.03	139	298.91	0(0, 111.57)	-1.11(-12.47, 10.25)
Male	Total	Septicemia	All Sites	6408	4536.03	1573006	9370220.61	1.41#(1.38, 1.45)	2(1.78, 2.22)
Male	Total	Septicemia	Oral Cavity and Pharynx	208	98.91	49561	299739.76	2.10#(1.83, 2.41)	3.64(2.49, 4.79)
Male	Total	Septicemia	Stomach	120	39.65	36651	85381.42	3.03#(2.51, 3.62)	9.41(6.51, 12.31)
Male	Total	Septicemia	Colon and Rectum	855	522.41	157916	962446.88	1.64#(1.53, 1.75)	3.46(2.7, 4.22)
Male	Total	Septicemia	Liver	120	12.54	29878	49070.03	9.57#(7.94, 11.45)	21.9(17.31, 26.49)
Male	Total	Septicemia	Pancreas	102	14.69	43939	40429.97	6.94#(5.66, 8.43)	21.59(16.36, 26.82)
Male	Total	Septicemia	Lung and Bronchus	571	149.94	232169	365694.93	3.81#(3.5, 4.13)	11.51(10.07, 12.95)
Male	Total	Septicemia	Skin excluding Basal and Squamous	178	198.21	65096	619115.07	0.9(0.77, 1.04)	-0.33(-0.94, 0.28)
Male	Total	Septicemia	Prostate	2079	2474.29	438459	3794486.79	0.84#(0.8, 0.88)	-1.04(-1.39, -0.69)
Male	Total	Septicemia	Urinary Bladder	531	366.55	81276	614579.53	1.45#(1.33, 1.58)	2.68(1.72, 3.64)
Male	Total	Septicemia	Kidney and Renal Pelvis	185	101.42	47509	285989.08	1.82#(1.57, 2.11)	2.92(1.76, 4.08)
Male	Total	Septicemia	Thyroid	31	31.1	16195	174873.39	1(0.68, 1.41)	-0.01(-0.89, 0.87)
Male	Total	Septicemia	Lymphoma	318	143.89	79456	554350.57	2.21#(1.97, 2.47)	3.14(2.38, 3.9)
Male	Total	Septicemia	Leukemia	197	85.92	53041	288066.73	2.29#(1.98, 2.64)	3.86(2.72, 5)

Table S7

Septicemia SMRs and AERs by cancer type, time since diagnosis, and duration for female patients

Sex	Latency	Selected Events	Site recode ICD-O-3/WHO 2008	Observed	Expected	Persons	Person Years at Risk	SMR(95%CI)	AER(95%CI)
Female	0-11 months	Septicemia	All Sites	1363	344.96	1494691	1229603.78	3.95#(3.74, 4.17)	8.28(7.62, 8.94)
Female	0-11 months	Septicemia	Oral Cavity and Pharynx	26	5.47	22358	19457.7	4.75#(3.1, 6.96)	10.55(4.9, 16.2)
Female	0-11 months	Septicemia	Stomach	40	5.61	22753	14301.82	7.13#(5.09, 9.7)	24.04(14.8, 33.28)
Female	0-11 months	Septicemia	Colon and Rectum	256	55.84	158955	131352	4.58#(4.04, 5.18)	15.24(12.61, 17.87)
Female	0-11 months	Septicemia	Liver	29	1.73	10715	5718.69	16.72#(11.2, 24.01)	47.68(28.73, 66.63)
Female	0-11 months	Septicemia	Pancreas	75	7.92	43310	19997.39	9.47#(7.45, 11.88)	33.55(24.64, 42.46)
Female	0-11 months	Septicemia	Lung and Bronchus	172	33.63	165079	102150.19	5.11#(4.38, 5.94)	13.55(10.8, 16.3)
Female	0-11 months	Septicemia	Skin excluding Basal and Squamous	10	11.31	58202	54752.97	0.88(0.42, 1.63)	-0.24(-1.89, 1.41)
Female	0-11 months	Septicemia	Breast	164	102.33	435180	413476.27	1.60#(1.37, 1.87)	1.49(0.72, 2.26)
Female	0-11 months	Septicemia	Cervix Uteri	27	3.18	33146	29992.93	8.48#(5.59, 12.34)	7.94(4.35, 11.53)
Female	0-11 months	Septicemia	Corpus Uteri	58	20.97	99144	91805.92	2.77#(2.1, 3.57)	4.03(2.13, 5.93)
Female	0-11 months	Septicemia	Ovary	49	9.09	53219	42271.61	5.39#(3.99, 7.13)	9.44(5.91, 12.97)
Female	0-11 months	Septicemia	Urinary Bladder	34	12.02	29550	25470.35	2.83#(1.96, 3.95)	8.63(3.41, 13.85)
Female	0-11 months	Septicemia	Kidney and Renal Pelvis	23	7.01	29131	24093.93	3.28#(2.08, 4.93)	6.64(2.19, 11.09)
Female	0-11 months	Septicemia	Thyroid	5	3.98	52653	49866.05	1.26(0.41, 2.93)	0.21(-0.97, 1.39)
Female	0-11 months	Septicemia	Lymphoma	73	15.86	65880	55089.78	4.60#(3.61, 5.79)	10.37(7.02, 13.72)
Female	0-11 months	Septicemia	Leukemia	39	9.37	39767	29567.82	4.16#(2.96, 5.69)	10.02(5.41, 14.63)
Female	12-59 months	Septicemia	All Sites	1286	965.59	1088025	3300879.65	1.33#(1.26, 1.41)	0.97(0.69, 1.25)
Female	12-59 months	Septicemia	Oral Cavity and Pharynx	25	14.04	16964	48803.72	1.78#(1.15, 2.63)	2.25(-0.26, 4.76)
Female	12-59 months	Septicemia	Stomach	14	9.47	10124	22350.69	1.48(0.81, 2.48)	2.03(-2.22, 6.28)
Female	12-59 months	Septicemia	Colon and Rectum	183	163.94	116704	340245.71	1.12(0.96, 1.29)	0.56(-0.51, 1.63)
Female	12-59 months	Septicemia	Liver	12	1.98	3874	7828.07	6.06#(3.13, 10.58)	12.8(3.44, 22.16)
Female	12-59 months	Septicemia	Pancreas	21	4.71	10053	13725.1	4.46#(2.76, 6.82)	11.87(4.63, 19.11)
Female	12-59 months	Septicemia	Lung and Bronchus	112	46.37	69038	129972.82	2.42#(1.99, 2.91)	5.05(3.15, 6.95)
Female	12-59 months	Septicemia	Skin excluding Basal and Squamous	33	36.5	52482	178855.08	0.9(0.62, 1.27)	-0.2(-1.11, 0.71)
Female	12-59 months	Septicemia	Breast	371	371.14	395947	1309396.25	1(0.9, 1.11)	0(-0.41, 0.41)
Female	12-59 months	Septicemia	Cervix Uteri	28	8.77	27424	86401.6	3.19#(2.12, 4.61)	2.23(0.85, 3.61)
Female	12-59 months	Septicemia	Corpus Uteri	91	71.7	86092	283555.87	1.27#(1.02, 1.56)	0.68(-0.2, 1.56)
Female	12-59 months	Septicemia	Ovary	38	19.35	36707	95805.85	1.96#(1.39, 2.7)	1.95(0.4, 3.5)
Female	12-59 months	Septicemia	Urinary Bladder	46	35.43	22737	70864.79	1.3(0.95, 1.73)	1.49(-1.01, 3.99)
Female	12-59 months	Septicemia	Kidney and Renal Pelvis	29	19.72	21253	64998.49	1.47(0.98, 2.11)	1.43(-0.67, 3.53)
Female	12-59 months	Septicemia	Thyroid	8	15.19	48043	170196.89	0.53(0.23, 1.04)	-0.42(-0.97, 0.13)
Female	12-59 months	Septicemia	Lymphoma	53	44.98	49167	154363.05	1.18(0.88, 1.54)	0.52(-0.74, 1.78)
Female	12-59 months	Septicemia	Leukemia	51	24	25549	74180.34	2.12#(1.58, 2.79)	3.64(1.35, 5.93)
Female	60-119 months	Septicemia	All Sites	1024	910.99	654908	2604242.63	1.12#(1.06, 1.2)	0.43(0.1, 0.76)
Female	60-119 months	Septicemia	Oral Cavity and Pharynx	23	12.15	9460	36854.58	1.89#(1.2, 2.84)	2.94(-0.21, 6.09)
Female	60-119 months	Septicemia	Stomach	11	7.44	3734	13699.34	1.48(0.74, 2.64)	2.6(-3.54, 8.74)
Female	60-119 months	Septicemia	Colon and Rectum	154	154.95	65126	254308.75	0.99(0.84, 1.16)	-0.04(-1.39, 1.31)
Female	60-119 months	Septicemia	Liver	2	0.72	1048	3281.2	2.77(0.34, 10)	3.89(-5.96, 13.74)
Female	60-119 months	Septicemia	Pancreas	4	1.92	1528	4828.4	2.08(0.57, 5.32)	4.3(-5.57, 14.17)
Female	60-119 months	Septicemia	Lung and Bronchus	41	26.17	18168	60781.39	1.57#(1.12, 2.13)	2.44(-0.2, 5.08)
Female	60-119 months	Septicemia	Skin excluding Basal and Squamous	25	34.29	38107	159901.02	0.73(0.47, 1.08)	-0.58(-1.52, 0.36)
Female	60-119 months	Septicemia	Breast	382	386.76	269408	1077708.52	0.99(0.89, 1.09)	-0.04(-0.54, 0.46)
Female	60-119 months	Septicemia	Cervix Uteri	15	9.94	18264	79719.4	1.51(0.84, 2.49)	0.63(-0.6, 1.86)
Female	60-119 months	Septicemia	Corpus Uteri	90	84.78	59887	249866.26	1.06(0.85, 1.3)	0.21(-0.83, 1.25)
Female	60-119 months	Septicemia	Ovary	17	14.2	16442	62780.36	1.2(0.7, 1.92)	0.45(-1.29, 2.19)
Female	60-119 months	Septicemia	Urinary Bladder	43	33.44	14395	56948.47	1.29(0.93, 1.73)	1.68(-1.33, 4.69)
Female	60-119 months	Septicemia	Kidney and Renal Pelvis	35	17.97	12820	49722.63	1.95#(1.36, 2.71)	3.42(0.55, 6.29)
Female	60-119 months	Septicemia	Thyroid	12	17.31	37286	155216.12	0.69(0.36, 1.21)	-0.34(-1.02, 0.34)
Female	60-119 months	Septicemia	Lymphoma	46	39.25	31226	122664.97	1.17(0.86, 1.56)	0.55(-0.93, 2.03)
Female	60-119 months	Septicemia	Leukemia	21	17.8	14070	53204.45	1.18(0.73, 1.8)	0.6(-1.69, 2.89)
Female	120-239 months	Septicemia	All Sites	1176	1137.36	410419	2623773.45	1.03(0.98, 1.09)	0.15(-0.21, 0.51)
Female	120-239 months	Septicemia	Oral Cavity and Pharynx	18	13.68	5695	35759.77	1.32(0.78, 2.08)	1.21(-1.87, 4.29)
Female	120-239 months	Septicemia	Stomach	17	7.24	1977	11375.28	2.35#(1.37, 3.76)	8.58(0.1, 17.06)
Female	120-239 months	Septicemia	Colon and Rectum	179	178.07	39320	238265.72	1.01(0.86, 1.16)	0.04(-1.51, 1.59)
Female	120-239 months	Septicemia	Liver	2	0.49	412	2212.34	4.12(0.5, 14.88)	6.85(-7.12, 20.82)
Female	120-239 months	Septicemia	Pancreas	1	1.42	615	3116.04	0.7(0.02, 3.92)	-1.35(-11.13, 8.43)
Female	120-239 months	Septicemia	Lung and Bronchus	43	22.82	8042	42214.81	1.88#(1.36, 2.54)	4.78(1.01, 8.55)
Female	120-239 months	Septicemia	Skin excluding Basal and Squamous	30	45.55	26546	184151.75	0.66#(0.44, 0.94)	-0.84(-1.77, 0.09)
Female	120-239 months	Septicemia	Breast	424	505.23	170274	1075955.96	0.84#(0.76, 0.92)	-0.75(-1.31, -0.19)
Female	120-239 months	Septicemia	Cervix Uteri	35	18.37	14014	108929.84	1.91#(1.33, 2.65)	1.53(0.22, 2.84)
Female	120-239 months	Septicemia	Corpus Uteri	157	144.99	41294	281217.91	1.08(0.92, 1.27)	0.43(-0.78, 1.64)
Female	120-239 months	Septicemia	Ovary	24	20.14	9855	68375.99	1.19(0.76, 1.77)	0.56(-1.34, 2.46)
Female	120-239 months	Septicemia	Urinary Bladder	52	36.79	8813	53557.93	1.41#(1.06, 1.85)	2.84(-0.61, 6.29)
Female	120-239 months	Septicemia	Kidney and Renal Pelvis	30	18.28	7473	42927.28	1.64#(1.11, 2.34)	2.73(-0.44, 5.9)
Female	120-239 months	Septicemia	Thyroid	14	24.58	25224	166693.2	0.57#(0.31, 0.96)	-0.63(-1.36, 0.1)
Female	120-239 months	Septicemia	Lymphoma	53	38.38	19065	116901.58	1.38#(1.03, 1.81)	1.25(-0.35, 2.85)
Female	120-239 months	Septicemia	Leukemia	21	14.83	7836	47075.68	1.42(0.88, 2.16)	1.31(-1.18, 3.8)
Female	240-359 months	Septicemia	All Sites	519	481.29	149952	875265.72	1.08(0.99, 1.18)	0.43(-0.28, 1.14)
Female	240-359 months	Septicemia	Oral Cavity and Pharynx	5	5.73	2035	12020.18	0.87(0.28, 2.04)	-0.61(-5.95, 4.73)
Female	240-359 months	Septicemia	Stomach	5	2.12	564	2993.47	2.36(0.77, 5.51)	9.63(-7.83, 27.09)
Female	240-359 months	Septicemia	Colon and Rectum	69	61.64	12342	66653.36	1.12(0.87, 1.42)	1.1(-2.26, 4.46)
Female	240-359 months	Septicemia	Liver	0	0.11	98	560.99	0(0, 32.7)	-2.01(-13.6, 9.58)
Female	240-359 months	Septicemia	Pancreas	0	0.36	137	754.78	0(0, 10.18)	-4.8(-20.38, 10.78)
Female	240-359 months	Septicemia	Lung and Bronchus	9	6.06	1845	9068.99	1.48(0.68, 2.82)	3.24(-5.14, 11.62)
Female	240-359 months	Septicemia	Skin excluding Basal and Squamous	25	24.84	11836	76103.14	1.01(0.65, 1.49)	0.02(-1.8, 1.84)
Female	240-359 months	Septicemia	Breast	173	203.74	59024	325296.51	0.85#(0.73, 0.99)	-0.95(-2.12, 0.22)
Female	240-359 months	Septicemia	Cervix Uteri	25	14.5	8060	55669.43	1.72#(1.12, 2.55)	1.89(-0.32, 4.1)
Female	240-359 months	Septicemia	Corpus Uteri	114	85.93	17286	101443.24	1.33#(1.09, 1.59)	2.77(0.04, 5.5)
Female	240-359 months	Septicemia	Ovary	9	11.98	4538	29856.91	0.75(0.34, 1.43)	-1(-4.01, 2.01)
Female	240-359 months	Septicemia	Urinary Bladder	22	12.66	2836	15425.87	1.74#(1.09, 2.63)	6.06(-1.42, 13.54)
Female	240-359 months	Septicemia	Kidney and Renal Pelvis	6	5.89	2170	12087.76	1.02(0.37, 2.22)	0.09(-5.5, 5.68)
Female	240-359 months	Septicemia	Thyroid	15	14.79	10283	67239.03	1.01(0.57, 1.67)	0.03(-1.56, 1.62)
Female	240-359 months	Septicemia	Lymphoma	9	11.4	6323	36480.76	0.79(0.36, 1.5)	-0.66(-3.09, 1.77)
Female	240-359 months	Septicemia	Leukemia	3	3.66	2573	15301.71	0.82(0.17, 2.4)	-0.43(-3.74, 2.88)
Female	360-479 months	Septicemia	All Sites	138	134.9	42904	210645.69	1.02(0.86, 1.21)	0.15(-1.39, 1.69)
Female	360-479 months	Septicemia	Oral Cavity and Pharynx	2	1.55	604	2978.74	1.29(0.16, 4.66)	1.51(-10.88, 13.9)
Female	360-479 months	Septicemia	Stomach	1	0.33	128	471.26	3.01(0.08, 16.8)	14.18(-33.74, 62.1)
Female	360-479 months	Septicemia	Colon and Rectum	13	13.05	2884	12783.64	1(0.53, 1.7)	-0.04(-7.86, 7.78)
Female	360-479 months	Septicemia	Liver	0	0.01	29	119.14	0(0, 586.24)	-0.53(-16.98, 15.92)
Female	360-479 months	Septicemia	Pancreas	0	0.07	34	156.74	0(0, 55.94)	-4.21(-37.29, 28.87)
Female	360-479 months	Septicemia	Lung and Bronchus	0	1.04	355	1508.71	0(0, 3.55)	-6.88(-20.12, 6.36)
Female	360-479 months	Septicemia	Skin excluding Basal and Squamous	10	9.69	4318	22376.92	1.03(0.49, 1.9)	0.14(-3.75, 4.03)
Female	360-479 months	Septicemia	Breast	51	52.25	14304	64954.76	0.98(0.73, 1.28)	-0.19(-3.25, 2.87)
Female	360-479 months	Septicemia	Cervix Uteri	12	7.25	3341	18576.45	1.65(0.86, 2.89)	2.56(-2.07, 7.19)
Female	360-479 months	Septicemia	Corpus Uteri	28	26.32	4920	23575.09	1.06(0.71, 1.54)	0.71(-5.41, 6.83)
Female	360-479 months	Septicemia	Ovary	3	4.38	1676	9080.51	0.69(0.14, 2)	-1.52(-7.38, 4.34)
Female	360-479 months	Septicemia	Urinary Bladder	1	2.85	690	3274.17	0.35(0.01, 1.95)	-5.66(-17.4, 6.08)
Female	360-479 months	Septicemia	Kidney and Renal Pelvis	0	1.28	575	2803.13	0(0, 2.87)	-4.58(-12.49, 3.33)
Female	360-479 months	Septicemia	Thyroid	5	7.31	3942	22009.26	0.68(0.22, 1.6)	-1.05(-4.17, 2.07)
Female	360-479 months	Septicemia	Lymphoma	5	2.65	1762	8206.79	1.88(0.61, 4.4)	2.86(-3.74, 9.46)
Female	360-479 months	Septicemia	Leukemia	1	0.66	800	4280.68	1.51(0.04, 8.4)	0.79(-5.11, 6.69)
Female	480+ months	Septicemia	All Sites	12	9.77	6481	14075.19	1.23(0.63, 2.15)	1.59(-4.9, 8.08)
Female	480+ months	Septicemia	Oral Cavity and Pharynx	1	0.12	95	232.21	8.51(0.22, 47.44)	38.01(-51.14, 127.16)
Female	480+ months	Septicemia	Stomach	0	0.02	9	22.13	0(0, 209.06)	-7.97(-133.17, 117.23)
Female	480+ months	Septicemia	Colon and Rectum	0	0.65	330	654.25	0(0, 5.67)	-9.94(-34.08, 14.2)
Female	480+ months	Septicemia	Liver	0	0	3	4.38	0(0, 18550.23)	-0.45(-0.45, -0.45)
Female	480+ months	Septicemia	Pancreas	0	0	3	3.62	0(0, 2500.98)	-4.08(-4.08, -4.08)
Female	480+ months	Septicemia	Lung and Bronchus	0	0.04	43	96.2	0(0, 92.7)	-4.14(-44.88, 36.6)
Female	480+ months	Septicemia	Skin excluding Basal and Squamous	1	0.81	706	1530.1	1.23(0.03, 6.85)	1.22(-16.01, 18.45)
Female	480+ months	Septicemia	Breast	4	3.76	1790	3691.28	1.06(0.29, 2.72)	0.64(-14.14, 15.42)
Female	480+ months	Septicemia	Cervix Uteri	1	0.81	692	1597.18	1.24(0.03, 6.9)	1.2(-15.31, 17.71)
Female	480+ months	Septicemia	Corpus Uteri	3	1.66	663	1399.77	1.81(0.37, 5.28)	9.58(-20.62, 39.78)
Female	480+ months	Septicemia	Ovary	0	0.34	302	675.19	0(0, 10.8)	-5.06(-21.98, 11.86)
Female	480+ months	Septicemia	Urinary Bladder	0	0.15	89	182.16	0(0, 23.95)	-8.46(-50.12, 33.2)
Female	480+ months	Septicemia	Kidney and Renal Pelvis	0	0.07	86	192.3	0(0, 54.59)	-3.51(-30.47, 23.45)
Female	480+ months	Septicemia	Thyroid	0	0.83	838	1947.22	0(0, 4.42)	-4.28(-13.45, 4.89)
Female	480+ months	Septicemia	Lymphoma	0	0.16	227	509.18	0(0, 22.85)	-3.17(-18.56, 12.22)
Female	480+ months	Septicemia	Leukemia	0	0.04	155	339.23	0(0, 87.02)	-1.25(-12.8, 10.3)
Female	Total	Septicemia	All Sites	5518	3984.86	1494691	10858486.12	1.38#(1.35, 1.42)	1.41(1.23, 1.59)
Female	Total	Septicemia	Oral Cavity and Pharynx	100	52.74	22358	156106.9	1.90#(1.54, 2.31)	3.03(1.48, 4.58)
Female	Total	Septicemia	Stomach	88	32.23	22753	65213.99	2.73#(2.19, 3.36)	8.55(5.26, 11.84)
Female	Total	Septicemia	Colon and Rectum	854	628.13	158955	1044263.43	1.36#(1.27, 1.45)	2.16(1.44, 2.88)
Female	Total	Septicemia	Liver	45	5.04	10715	19724.82	8.92#(6.51, 11.94)	20.26(13.24, 27.28)
Female	Total	Septicemia	Pancreas	101	16.4	43310	42582.07	6.16#(5.02, 7.48)	19.87(14.89, 24.85)
Female	Total	Septicemia	Lung and Bronchus	377	136.14	165079	345793.11	2.77#(2.5, 3.06)	6.97(5.69, 8.25)
Female	Total	Septicemia	Skin excluding Basal and Squamous	134	162.99	58202	677670.98	0.82#(0.69, 0.97)	-0.43(-0.93, 0.07)
Female	Total	Septicemia	Breast	1569	1625.2	435180	4270479.55	0.97(0.92, 1.01)	-0.13(-0.39, 0.13)
Female	Total	Septicemia	Cervix Uteri	143	62.82	33146	380886.83	2.28#(1.92, 2.68)	2.11(1.37, 2.85)
Female	Total	Septicemia	Corpus Uteri	541	436.36	99144	1032864.05	1.24#(1.14, 1.35)	1.01(0.42, 1.6)
Female	Total	Septicemia	Ovary	140	79.47	53219	308846.41	1.76#(1.48, 2.08)	1.96(1.02, 2.9)
Female	Total	Septicemia	Urinary Bladder	198	133.35	29550	225723.74	1.48#(1.29, 1.71)	2.86(1.28, 4.44)
Female	Total	Septicemia	Kidney and Renal Pelvis	123	70.22	29131	196825.52	1.75#(1.46, 2.09)	2.68(1.3, 4.06)
Female	Total	Septicemia	Thyroid	59	84	52653	633167.76	0.70#(0.53, 0.91)	-0.39(-0.76, -0.02)
Female	Total	Septicemia	Lymphoma	239	152.69	65880	494216.11	1.57#(1.37, 1.78)	1.75(0.97, 2.53)
Female	Total	Septicemia	Leukemia	136	70.37	39767	223949.91	1.93#(1.62, 2.29)	2.93(1.67, 4.19)

Table S8

Septicemia SMRs and AERs by cancer site and year of diagnosis for male patients

Sex	Year of diagnosis_by10	Selected Events	Site recode ICD-O-3/WHO 2008	Observed	Expected	Persons	Person Years at Risk	SMR(95%CI)	AER(95%CI)
Male	1975-79	Septicemia	All Sites	353	223.8	109299	605101.16	1.58#(1.42, 1.75)	2.14(1.36, 2.92)
Male	1975-79	Septicemia	Oral Cavity and Pharynx	25	11.12	4569	32646.72	2.25#(1.45, 3.32)	4.25(0.64, 7.86)
Male	1975-79	Septicemia	Stomach	4	2.76	3986	7973.29	1.45(0.39, 3.71)	1.55(-4.84, 7.94)
Male	1975-79	Septicemia	Colon and Rectum	49	42.14	14597	87340.23	1.16(0.86, 1.54)	0.79(-1.35, 2.93)
Male	1975-79	Septicemia	Liver	1	0.06	948	595.88	17.64(0.45, 98.26)	15.83(-18.01, 49.67)
Male	1975-79	Septicemia	Pancreas	2	0.48	3652	2423.59	4.14(0.5, 14.94)	6.26(-6.47, 18.99)
Male	1975-79	Septicemia	Lung and Bronchus	29	9.73	23984	39623.31	2.98#(2, 4.28)	4.86(1.78, 7.94)
Male	1975-79	Septicemia	Skin excluding Basal and Squamous	6	10.33	2463	39758.02	0.58(0.21, 1.26)	-1.09(-3.08, 0.9)
Male	1975-79	Septicemia	Prostate	100	80.57	20132	138366.39	1.24#(1.01, 1.51)	1.4(-0.5, 3.3)
Male	1975-79	Septicemia	Urinary Bladder	50	26.9	6426	59061.44	1.86#(1.38, 2.45)	3.91(1, 6.82)
Male	1975-79	Septicemia	Kidney and Renal Pelvis	10	5.19	2384	16964.91	1.93(0.92, 3.54)	2.84(-1.66, 7.34)
Male	1975-79	Septicemia	Thyroid	2	2.92	625	16038.87	0.68(0.08, 2.47)	-0.58(-3.29, 2.13)
Male	1975-79	Septicemia	Lymphoma	15	6.17	4341	38566.35	2.43#(1.36, 4.01)	2.29(-0.05, 4.63)
Male	1975-79	Septicemia	Leukemia	15	3.84	4132	20223.83	3.90#(2.18, 6.44)	5.52(1.31, 9.73)
Male	1980-89	Septicemia	All Sites	1288	796.85	265812	1647689.19	1.62#(1.53, 1.71)	2.98(2.44, 3.52)
Male	1980-89	Septicemia	Oral Cavity and Pharynx	61	23.92	9047	63907.8	2.55#(1.95, 3.28)	5.8(2.97, 8.63)
Male	1980-89	Septicemia	Stomach	23	8.97	8322	18347.55	2.57#(1.63, 3.85)	7.65(1.61, 13.69)
Male	1980-89	Septicemia	Colon and Rectum	206	139.02	33061	225400.08	1.48#(1.29, 1.7)	2.97(1.36, 4.58)
Male	1980-89	Septicemia	Liver	6	0.51	2515	2174.75	11.85#(4.35, 25.79)	25.26(2.29, 48.23)
Male	1980-89	Septicemia	Pancreas	10	1.86	7648	5180.36	5.38#(2.58, 9.89)	15.71(2.69, 28.73)
Male	1980-89	Septicemia	Lung and Bronchus	114	29.95	54204	86634.02	3.81#(3.14, 4.57)	9.7(6.99, 12.41)
Male	1980-89	Septicemia	Skin excluding Basal and Squamous	39	34.8	7526	121939.81	1.12(0.8, 1.53)	0.34(-1.04, 1.72)
Male	1980-89	Septicemia	Prostate	409	354.88	56091	450825.8	1.15#(1.04, 1.27)	1.2(0, 2.4)
Male	1980-89	Septicemia	Urinary Bladder	122	85.62	14854	150407.74	1.42#(1.18, 1.7)	2.42(0.54, 4.3)
Male	1980-89	Septicemia	Kidney and Renal Pelvis	26	16.62	6136	46039	1.56#(1.02, 2.29)	2.04(-0.74, 4.82)
Male	1980-89	Septicemia	Thyroid	7	5.82	1408	31338.53	1.2(0.48, 2.48)	0.38(-1.86, 2.62)
Male	1980-89	Septicemia	Lymphoma	60	22.98	12392	112825.57	2.61#(1.99, 3.36)	3.28(1.7, 4.86)
Male	1980-89	Septicemia	Leukemia	36	14.27	9372	57004.82	2.52#(1.77, 3.49)	3.81(1.37, 6.25)
Male	1990-99	Septicemia	All Sites	1818	1523.39	339075	2668546.83	1.19#(1.14, 1.25)	1.1(0.68, 1.52)
Male	1990-99	Septicemia	Oral Cavity and Pharynx	49	24.76	9057	69699.76	1.98#(1.46, 2.62)	3.48(1.07, 5.89)
Male	1990-99	Septicemia	Stomach	36	10.62	7999	20625.01	3.39#(2.37, 4.69)	12.3(5.82, 18.78)
Male	1990-99	Septicemia	Colon and Rectum	241	160.17	34128	257966.16	1.50#(1.32, 1.71)	3.13(1.61, 4.65)
Male	1990-99	Septicemia	Liver	13	1.32	4474	5827.7	9.88#(5.26, 16.9)	20.05(7.34, 32.76)
Male	1990-99	Septicemia	Pancreas	24	2.6	8090	6812.07	9.22#(5.91, 13.72)	31.41(16.59, 46.23)
Male	1990-99	Septicemia	Lung and Bronchus	111	37.35	54133	87957.04	2.97#(2.45, 3.58)	8.37(5.66, 11.08)
Male	1990-99	Septicemia	Skin excluding Basal and Squamous	48	53.3	11753	171692.79	0.9(0.66, 1.19)	-0.31(-1.46, 0.84)
Male	1990-99	Septicemia	Prostate	815	968.35	103463	1226415.74	0.84#(0.78, 0.9)	-1.25(-1.92, -0.58)
Male	1990-99	Septicemia	Urinary Bladder	142	105.01	16477	164569.16	1.35#(1.14, 1.59)	2.25(0.38, 4.12)
Male	1990-99	Septicemia	Kidney and Renal Pelvis	51	25.87	7921	64066.07	1.97#(1.47, 2.59)	3.92(1.24, 6.6)
Male	1990-99	Septicemia	Thyroid	8	7.63	2263	42008.11	1.05(0.45, 2.07)	0.09(-1.75, 1.93)
Male	1990-99	Septicemia	Lymphoma	71	37.84	17718	150386.96	1.88#(1.47, 2.37)	2.21(0.85, 3.57)
Male	1990-99	Septicemia	Leukemia	40	20.64	10836	73811.34	1.94#(1.38, 2.64)	2.62(0.55, 4.69)
Male	2000-2009	Septicemia	All Sites	1845	1428.97	395848	2967812.75	1.29#(1.23, 1.35)	1.4(1.02, 1.78)
Male	2000-2009	Septicemia	Oral Cavity and Pharynx	45	24.23	10796	80247.17	1.86#(1.35, 2.49)	2.59(0.56, 4.62)
Male	2000-2009	Septicemia	Stomach	36	11.15	7923	23012.53	3.23#(2.26, 4.47)	10.8(4.96, 16.64)
Male	2000-2009	Septicemia	Colon and Rectum	242	134.19	36613	263324.44	1.80#(1.58, 2.05)	4.09(2.65, 5.53)
Male	2000-2009	Septicemia	Liver	45	4.69	8494	18475.97	9.59#(7, 12.83)	21.82(14.35, 29.29)
Male	2000-2009	Septicemia	Pancreas	31	4.52	10373	11371.32	6.86#(4.66, 9.74)	23.29(13.03, 33.55)
Male	2000-2009	Septicemia	Lung and Bronchus	169	42.93	50947	86307.89	3.94#(3.37, 4.58)	14.61(11.31, 17.91)
Male	2000-2009	Septicemia	Skin excluding Basal and Squamous	54	62.79	17579	184659.7	0.86(0.65, 1.12)	-0.48(-1.63, 0.67)
Male	2000-2009	Septicemia	Prostate	596	830.02	125668	1405701.72	0.72#(0.66, 0.78)	-1.66(-2.19, -1.13)
Male	2000-2009	Septicemia	Urinary Bladder	124	99.27	18926	154613.62	1.25#(1.04, 1.49)	1.6(-0.29, 3.49)
Male	2000-2009	Septicemia	Kidney and Renal Pelvis	56	34.24	12263	95642.35	1.64#(1.24, 2.12)	2.28(0.33, 4.23)
Male	2000-2009	Septicemia	Thyroid	9	9.44	4348	53140.73	0.95(0.44, 1.81)	-0.08(-1.66, 1.5)
Male	2000-2009	Septicemia	Lymphoma	108	50.14	20405	166699.45	2.15#(1.77, 2.6)	3.47(1.99, 4.95)
Male	2000-2009	Septicemia	Leukemia	57	30.06	12965	86697.38	1.90#(1.44, 2.46)	3.11(1, 5.22)
Male	2010-19	Septicemia	All Sites	1104	563.03	462972	1481070.67	1.96#(1.85, 2.08)	3.65(3.11, 4.19)
Male	2010-19	Septicemia	Oral Cavity and Pharynx	28	14.88	16092	53238.31	1.88#(1.25, 2.72)	2.47(0.06, 4.88)
Male	2010-19	Septicemia	Stomach	21	6.15	8421	15423.04	3.42#(2.12, 5.22)	9.63(3.01, 16.25)
Male	2010-19	Septicemia	Colon and Rectum	117	46.89	39517	128415.97	2.50#(2.06, 2.99)	5.46(3.51, 7.41)
Male	2010-19	Septicemia	Liver	55	5.97	13447	21995.73	9.22#(6.95, 12)	22.29(15.34, 29.24)
Male	2010-19	Septicemia	Pancreas	35	5.23	14176	14642.62	6.69#(4.66, 9.31)	20.33(11.85, 28.81)
Male	2010-19	Septicemia	Lung and Bronchus	148	29.99	48901	65172.68	4.93#(4.17, 5.8)	18.11(14.1, 22.12)
Male	2010-19	Septicemia	Skin excluding Basal and Squamous	31	36.99	25775	101064.74	0.84(0.57, 1.19)	-0.59(-2.19, 1.01)
Male	2010-19	Septicemia	Prostate	159	240.47	133105	573177.15	0.66#(0.56, 0.77)	-1.42(-2.1, -0.74)
Male	2010-19	Septicemia	Urinary Bladder	93	49.76	24593	85927.57	1.87#(1.51, 2.29)	5.03(2.31, 7.75)
Male	2010-19	Septicemia	Kidney and Renal Pelvis	42	19.5	18805	63276.75	2.15#(1.55, 2.91)	3.56(1.13, 5.99)
Male	2010-19	Septicemia	Thyroid	5	5.29	7551	32347.14	0.95(0.31, 2.21)	-0.09(-2.03, 1.85)
Male	2010-19	Septicemia	Lymphoma	64	26.76	24600	85872.24	2.39#(1.84, 3.05)	4.34(2.17, 6.51)
Male	2010-19	Septicemia	Leukemia	49	17.1	15736	50329.37	2.86#(2.12, 3.79)	6.34(3.18, 9.5)

Table S9

Septicemia SMRs and AERs by cancer site and year of diagnosis for female patients

Sex	Year of diagnosis_by10	Selected Events	Site recode ICD-O-3/WHO 2008	Observed	Expected	Persons	Person Years at Risk	SMR(95%CI)	AER(95%CI)
Female	1975-79	Septicemia	All Sites	396	368.28	106618	1053085.01	1.08(0.97, 1.19)	0.26(-0.25, 0.77)
Female	1975-79	Septicemia	Oral Cavity and Pharynx	6	5.15	1987	17381.74	1.17(0.43, 2.54)	0.49(-3.27, 4.25)
Female	1975-79	Septicemia	Stomach	6	2.69	2584	6904.41	2.23(0.82, 4.86)	4.8(-3.57, 13.17)
Female	1975-79	Septicemia	Colon and Rectum	52	60.57	15671	117095.38	0.86(0.64, 1.13)	-0.73(-2.51, 1.05)
Female	1975-79	Septicemia	Liver	2	0.12	460	564.31	16.38#(1.98, 59.17)	33.28(-17.21, 83.77)
Female	1975-79	Septicemia	Pancreas	1	0.52	3164	2282.16	1.93(0.05, 10.74)	2.11(-8.48, 12.7)
Female	1975-79	Septicemia	Lung and Bronchus	10	5.36	9001	23008.7	1.87(0.9, 3.43)	2.02(-1.32, 5.36)
Female	1975-79	Septicemia	Skin excluding Basal and Squamous	17	13.64	2582	56167.67	1.25(0.73, 2)	0.6(-1.33, 2.53)
Female	1975-79	Septicemia	Breast	121	127.63	27209	347601.58	0.95(0.79, 1.13)	-0.19(-1.08, 0.7)
Female	1975-79	Septicemia	Cervix Uteri	26	13.73	3827	64761.46	1.89#(1.24, 2.78)	1.9(-0.01, 3.81)
Female	1975-79	Septicemia	Corpus Uteri	69	77.78	9225	170262.53	0.89(0.69, 1.12)	-0.52(-1.91, 0.87)
Female	1975-79	Septicemia	Ovary	13	8.45	4760	35425.18	1.54(0.82, 2.63)	1.28(-1.28, 3.84)
Female	1975-79	Septicemia	Urinary Bladder	9	12.23	2527	25291.99	0.74(0.34, 1.4)	-1.28(-4.85, 2.29)
Female	1975-79	Septicemia	Kidney and Renal Pelvis	5	4	1430	12896.29	1.25(0.41, 2.92)	0.78(-3.78, 5.34)
Female	1975-79	Septicemia	Thyroid	8	9.32	1839	53511.37	0.86(0.37, 1.69)	-0.25(-1.77, 1.27)
Female	1975-79	Septicemia	Lymphoma	10	7.37	3954	35547.89	1.36(0.65, 2.49)	0.74(-1.56, 3.04)
Female	1975-79	Septicemia	Leukemia	6	4.27	3250	19170.02	1.41(0.52, 3.06)	0.9(-2.38, 4.18)
Female	1980-89	Septicemia	All Sites	1254	975.02	253323	2412581.1	1.29#(1.22, 1.36)	1.16(0.78, 1.54)
Female	1980-89	Septicemia	Oral Cavity and Pharynx	28	14.14	4435	39559.82	1.98#(1.32, 2.86)	3.5(0.28, 6.72)
Female	1980-89	Septicemia	Stomach	17	8.13	4969	15168.22	2.09#(1.22, 3.35)	5.85(-0.62, 12.32)
Female	1980-89	Septicemia	Colon and Rectum	231	178.81	35008	268268.92	1.29#(1.13, 1.47)	1.95(0.47, 3.43)
Female	1980-89	Septicemia	Liver	4	0.36	1193	1990.2	11.05#(3.01, 28.3)	18.28(-2.26, 38.82)
Female	1980-89	Septicemia	Pancreas	9	2.39	7738	6542.52	3.76#(1.72, 7.13)	10.1(0, 20.2)
Female	1980-89	Septicemia	Lung and Bronchus	42	20.12	27966	63095.05	2.09#(1.5, 2.82)	3.47(1.02, 5.92)
Female	1980-89	Septicemia	Skin excluding Basal and Squamous	35	38.56	7601	160091.1	0.91(0.63, 1.26)	-0.22(-1.27, 0.83)
Female	1980-89	Septicemia	Breast	409	395.77	68757	908776.55	1.03(0.94, 1.14)	0.15(-0.46, 0.76)
Female	1980-89	Septicemia	Cervix Uteri	48	21.12	7092	113911.52	2.27#(1.68, 3.01)	2.36(0.93, 3.79)
Female	1980-89	Septicemia	Corpus Uteri	138	125.82	16110	248466.59	1.1(0.92, 1.3)	0.49(-0.79, 1.77)
Female	1980-89	Septicemia	Ovary	34	21.31	10763	80505.75	1.60#(1.1, 2.23)	1.58(-0.23, 3.39)
Female	1980-89	Septicemia	Urinary Bladder	55	33.78	5565	57093.09	1.63#(1.23, 2.12)	3.72(0.49, 6.95)
Female	1980-89	Septicemia	Kidney and Renal Pelvis	22	12.27	3793	34253.27	1.79#(1.12, 2.72)	2.84(-0.51, 6.19)
Female	1980-89	Septicemia	Thyroid	11	18.93	4481	119874.87	0.58(0.29, 1.04)	-0.66(-1.55, 0.23)
Female	1980-89	Septicemia	Lymphoma	47	27.26	10489	99745.34	1.72#(1.27, 2.29)	1.98(0.29, 3.67)
Female	1980-89	Septicemia	Leukemia	24	12.79	7139	45787.58	1.88#(1.2, 2.79)	2.45(-0.15, 5.05)
Female	1990-99	Septicemia	All Sites	1469	1181.95	307460	2925709.3	1.24#(1.18, 1.31)	0.98(0.64, 1.32)
Female	1990-99	Septicemia	Oral Cavity and Pharynx	23	14.54	4528	40503.6	1.58#(1, 2.37)	2.09(-0.87, 5.05)
Female	1990-99	Septicemia	Stomach	34	9.02	4899	15529.56	3.77#(2.61, 5.27)	16.09(7.82, 24.36)
Female	1990-99	Septicemia	Colon and Rectum	225	192.06	35400	280871.33	1.17#(1.02, 1.33)	1.17(-0.25, 2.59)
Female	1990-99	Septicemia	Liver	7	0.77	1877	3166.93	9.09#(3.65, 18.73)	19.67(2.44, 36.9)
Female	1990-99	Septicemia	Pancreas	18	3.45	8665	8207.01	5.22#(3.09, 8.25)	17.73(6.68, 28.78)
Female	1990-99	Septicemia	Lung and Bronchus	104	34.91	38624	86069.4	2.98#(2.43, 3.61)	8.03(5.35, 10.71)
Female	1990-99	Septicemia	Skin excluding Basal and Squamous	31	44.18	10562	184837.19	0.70#(0.48, 1)	-0.71(-1.63, 0.21)
Female	1990-99	Septicemia	Breast	497	529.65	89552	1233454.19	0.94(0.86, 1.02)	-0.26(-0.77, 0.25)
Female	1990-99	Septicemia	Cervix Uteri	30	16.79	7679	110662.28	1.79#(1.21, 2.55)	1.19(-0.02, 2.4)
Female	1990-99	Septicemia	Corpus Uteri	159	118.4	17893	250338.81	1.34#(1.14, 1.57)	1.62(0.32, 2.92)
Female	1990-99	Septicemia	Ovary	23	22.32	12002	83012.06	1.03(0.65, 1.55)	0.08(-1.51, 1.67)
Female	1990-99	Septicemia	Urinary Bladder	50	39.21	6284	60809.99	1.28(0.95, 1.68)	1.77(-1.27, 4.81)
Female	1990-99	Septicemia	Kidney and Renal Pelvis	24	18.27	5217	46614.77	1.31(0.84, 1.95)	1.23(-1.5, 3.96)
Female	1990-99	Septicemia	Thyroid	19	19.9	7260	150842.1	0.95(0.57, 1.49)	-0.06(-0.87, 0.75)
Female	1990-99	Septicemia	Lymphoma	55	44.07	14226	136710.75	1.25(0.94, 1.62)	0.8(-0.63, 2.23)
Female	1990-99	Septicemia	Leukemia	30	18.13	8163	57291.57	1.65#(1.12, 2.36)	2.07(-0.3, 4.44)
Female	2000-2009	Septicemia	All Sites	1497	1022.25	370962	2937169.87	1.46#(1.39, 1.54)	1.62(1.29, 1.95)
Female	2000-2009	Septicemia	Oral Cavity and Pharynx	25	12.4	4927	37086.4	2.02#(1.31, 2.98)	3.4(0.17, 6.63)
Female	2000-2009	Septicemia	Stomach	20	8.33	4943	16892.69	2.40#(1.47, 3.71)	6.91(0.74, 13.08)
Female	2000-2009	Septicemia	Colon and Rectum	231	150.11	36803	261285.95	1.54#(1.35, 1.75)	3.1(1.64, 4.56)
Female	2000-2009	Septicemia	Liver	14	1.8	2960	6813.88	7.79#(4.26, 13.07)	17.91(6.49, 29.33)
Female	2000-2009	Septicemia	Pancreas	30	5.21	10423	11997.28	5.76#(3.88, 8.22)	20.66(10.98, 30.34)
Female	2000-2009	Septicemia	Lung and Bronchus	129	44.77	43718	98513.76	2.88#(2.41, 3.42)	8.55(5.93, 11.17)
Female	2000-2009	Septicemia	Skin excluding Basal and Squamous	37	44.48	15671	186304.33	0.83(0.59, 1.15)	-0.4(-1.35, 0.55)
Female	2000-2009	Septicemia	Breast	371	412.96	108403	1194375.59	0.90#(0.81, 0.99)	-0.35(-0.81, 0.11)
Female	2000-2009	Septicemia	Cervix Uteri	26	8.3	6954	64995.63	3.13#(2.05, 4.59)	2.72(0.95, 4.49)
Female	2000-2009	Septicemia	Corpus Uteri	105	80.89	22388	234148.07	1.30#(1.06, 1.57)	1.03(-0.11, 2.17)
Female	2000-2009	Septicemia	Ovary	34	18.59	12766	72010.08	1.83#(1.27, 2.56)	2.14(0.17, 4.11)
Female	2000-2009	Septicemia	Urinary Bladder	61	34.06	6928	55124.63	1.79#(1.37, 2.3)	4.89(1.42, 8.36)
Female	2000-2009	Septicemia	Kidney and Renal Pelvis	44	24.13	7886	65307.46	1.82#(1.32, 2.45)	3.04(0.56, 5.52)
Female	2000-2009	Septicemia	Thyroid	14	24.45	15350	200485.47	0.57#(0.31, 0.96)	-0.52(-1.13, 0.09)
Female	2000-2009	Septicemia	Lymphoma	80	52.5	17486	151681.35	1.52#(1.21, 1.9)	1.81(0.32, 3.3)
Female	2000-2009	Septicemia	Leukemia	46	23.09	9729	64870.13	1.99#(1.46, 2.66)	3.53(1.02, 6.04)
Female	2010-19	Septicemia	All Sites	902	437.36	456328	1529940.84	2.06#(1.93, 2.2)	3.04(2.57, 3.51)
Female	2010-19	Septicemia	Oral Cavity and Pharynx	18	6.51	6481	21575.34	2.76#(1.64, 4.37)	5.32(0.82, 9.82)
Female	2010-19	Septicemia	Stomach	11	4.07	5358	10719.1	2.70#(1.35, 4.84)	6.47(-0.63, 13.57)
Female	2010-19	Septicemia	Colon and Rectum	115	46.57	36073	116741.85	2.47#(2.04, 2.96)	5.86(3.73, 7.99)
Female	2010-19	Septicemia	Liver	18	1.99	4225	7189.49	9.04#(5.36, 14.28)	22.27(10.1, 34.44)
Female	2010-19	Septicemia	Pancreas	43	4.82	13320	13553.11	8.91#(6.45, 12.01)	28.17(18.18, 38.16)
Female	2010-19	Septicemia	Lung and Bronchus	92	30.98	45770	75106.2	2.97#(2.39, 3.64)	8.12(5.23, 11.01)
Female	2010-19	Septicemia	Skin excluding Basal and Squamous	14	22.13	21786	90270.69	0.63(0.35, 1.06)	-0.9(-2.2, 0.4)
Female	2010-19	Septicemia	Breast	171	159.19	141259	586271.64	1.07(0.92, 1.25)	0.2(-0.41, 0.81)
Female	2010-19	Septicemia	Cervix Uteri	13	2.88	7594	26555.94	4.51#(2.4, 7.71)	3.81(0.87, 6.75)
Female	2010-19	Septicemia	Corpus Uteri	70	33.47	33528	129648.05	2.09#(1.63, 2.64)	2.82(1.28, 4.36)
Female	2010-19	Septicemia	Ovary	36	8.8	12928	37893.33	4.09#(2.87, 5.66)	7.18(3.72, 10.64)
Female	2010-19	Septicemia	Urinary Bladder	23	14.07	8246	27404.04	1.64#(1.04, 2.45)	3.26(-1.09, 7.61)
Female	2010-19	Septicemia	Kidney and Renal Pelvis	28	11.55	10805	37753.73	2.42#(1.61, 3.5)	4.36(1.1, 7.62)
Female	2010-19	Septicemia	Thyroid	7	11.39	23723	108453.94	0.61(0.25, 1.27)	-0.41(-1.18, 0.36)
Female	2010-19	Septicemia	Lymphoma	47	21.49	19725	70530.78	2.19#(1.61, 2.91)	3.62(1.32, 5.92)
Female	2010-19	Septicemia	Leukemia	30	12.09	11486	36830.61	2.48#(1.67, 3.54)	4.86(1.41, 8.31)

Table S10

Septicemia SMRs and AERs by cancer type and surgical status in male patients

Sex	surgery	Selected Events	Site recode ICD-O-3/WHO 2008	Observed	Expected	Persons	Person Years at Risk	SMR(95%CI)	AER(95%CI)
Male	yes	Septicemia	All Sites	3380	2696.51	741753	6053612.39	1.25#(1.21, 1.3)	1.13(0.88, 1.38)
Male	yes	Septicemia	Oral Cavity and Pharynx	130	74.77	27801	210489.29	1.74#(1.45, 2.06)	2.62(1.29, 3.95)
Male	yes	Septicemia	Stomach	84	32.2	18252	69790.77	2.61#(2.08, 3.23)	7.42(4.39, 10.45)
Male	yes	Septicemia	Colon and Rectum	759	502.88	133114	922444.37	1.51#(1.4, 1.62)	2.78(2.03, 3.53)
Male	yes	Septicemia	Liver	28	5.49	5885	25647.19	5.10#(3.39, 7.37)	8.78(4.36, 13.2)
Male	yes	Septicemia	Pancreas	33	5.2	6587	16809.34	6.34#(4.37, 8.91)	16.54(9.34, 23.74)
Male	yes	Septicemia	Lung and Bronchus	226	78.86	45659	196822.14	2.87#(2.5, 3.26)	7.48(5.74, 9.22)
Male	yes	Septicemia	Skin excluding Basal and Squamous	167	189.88	60221	595791.55	0.88(0.75, 1.02)	-0.38(-1, 0.24)
Male	yes	Septicemia	Prostate	873	1134.4	195340	1966066.62	0.77#(0.72, 0.82)	-1.33(-1.78, -0.88)
Male	yes	Septicemia	Urinary Bladder	499	351.07	75617	588455.89	1.42#(1.3, 1.55)	2.51(1.54, 3.48)
Male	yes	Septicemia	Kidney and Renal Pelvis	147	92.65	36934	270508.9	1.59#(1.34, 1.86)	2.01(0.89, 3.13)
Male	yes	Septicemia	Thyroid	26	30.07	15230	170958.22	0.86(0.56, 1.27)	-0.24(-1.1, 0.62)
Male	yes	Septicemia	Lymphoma	80	42.22	18408	162483.95	1.90#(1.5, 2.36)	2.33(1, 3.66)
Male	yes	Septicemia	Leukemia	3	1.12	436	4642.66	2.68(0.55, 7.82)	4.05(-4.52, 12.62)
Male	Unknown/no	Septicemia	All Sites	6408	4536.03	1573006	9370220.61	1.41#(1.38, 1.45)	2(1.78, 2.22)
Male	Unknown/no	Septicemia	Oral Cavity and Pharynx	208	98.91	49561	299739.76	2.10#(1.83, 2.41)	3.64(2.49, 4.79)
Male	Unknown/no	Septicemia	Stomach	120	39.65	36651	85381.42	3.03#(2.51, 3.62)	9.41(6.51, 12.31)
Male	Unknown/no	Septicemia	Colon and Rectum	855	522.41	157916	962446.88	1.64#(1.53, 1.75)	3.46(2.7, 4.22)
Male	Unknown/no	Septicemia	Liver	120	12.54	29878	49070.03	9.57#(7.94, 11.45)	21.9(17.31, 26.49)
Male	Unknown/no	Septicemia	Pancreas	102	14.69	43939	40429.97	6.94#(5.66, 8.43)	21.59(16.36, 26.82)
Male	Unknown/no	Septicemia	Lung and Bronchus	571	149.94	232169	365694.93	3.81#(3.5, 4.13)	11.51(10.07, 12.95)
Male	Unknown/no	Septicemia	Skin excluding Basal and Squamous	178	198.21	65096	619115.07	0.9(0.77, 1.04)	-0.33(-0.94, 0.28)
Male	Unknown/no	Septicemia	Prostate	2079	2474.29	438459	3794486.79	0.84#(0.8, 0.88)	-1.04(-1.39, -0.69)
Male	Unknown/no	Septicemia	Urinary Bladder	531	366.55	81276	614579.53	1.45#(1.33, 1.58)	2.68(1.72, 3.64)
Male	Unknown/no	Septicemia	Kidney and Renal Pelvis	185	101.42	47509	285989.08	1.82#(1.57, 2.11)	2.92(1.76, 4.08)
Male	Unknown/no	Septicemia	Thyroid	31	31.1	16195	174873.39	1(0.68, 1.41)	-0.01(-0.89, 0.87)
Male	Unknown/no	Septicemia	Lymphoma	318	143.89	79456	554350.57	2.21#(1.97, 2.47)	3.14(2.38, 3.9)
Male	Unknown/no	Septicemia	Leukemia	197	85.92	53041	288066.73	2.29#(1.98, 2.64)	3.86(2.72, 5)

Table S11

Septicemia SMRs and AERs by cancer type and surgical status in female patients

Sex	surgery	Selected Events	Site recode ICD-O-3/WHO 2008	Observed	Expected	Persons	Person Years at Risk	SMR(95%CI)	AER(95%CI)
Female	yes	Septicemia	All Sites	3892	3454.48	990381	9417435.06	1.13#(1.09, 1.16)	0.46(0.28, 0.64)
Female	yes	Septicemia	Oral Cavity and Pharynx	65	43.15	14470	124059.23	1.51#(1.16, 1.92)	1.76(0.12, 3.4)
Female	yes	Septicemia	Stomach	65	26.28	11321	54283.35	2.47#(1.91, 3.15)	7.13(3.68, 10.58)
Female	yes	Septicemia	Colon and Rectum	760	606.17	134051	1006913.09	1.25#(1.17, 1.35)	1.53(0.81, 2.25)
Female	yes	Septicemia	Liver	14	2.21	2277	11026	6.34#(3.46, 10.63)	10.69(3.54, 17.84)
Female	yes	Septicemia	Pancreas	25	5.36	6236	19131.51	4.67#(3.02, 6.89)	10.27(4.63, 15.91)
Female	yes	Septicemia	Lung and Bronchus	136	75.41	35196	199202.2	1.80#(1.51, 2.13)	3.04(1.61, 4.47)
Female	yes	Septicemia	Skin excluding Basal and Squamous	121	156.4	54681	654108.5	0.77#(0.64, 0.92)	-0.54(-1.04, -0.04)
Female	yes	Septicemia	Breast	1400	1570.67	400378	4152209.71	0.89#(0.85, 0.94)	-0.41(-0.67, -0.15)
Female	yes	Septicemia	Cervix Uteri	63	40.17	19252	292153.64	1.57#(1.21, 2.01)	0.78(0.1, 1.46)
Female	yes	Septicemia	Corpus Uteri	483	418.58	90369	993312.15	1.15#(1.05, 1.26)	0.65(0.06, 1.24)
Female	yes	Septicemia	Ovary	112	72.3	40235	292481.66	1.55#(1.28, 1.86)	1.36(0.45, 2.27)
Female	yes	Septicemia	Urinary Bladder	182	127.22	26783	215364.34	1.43#(1.23, 1.65)	2.54(0.94, 4.14)
Female	yes	Septicemia	Kidney and Renal Pelvis	100	62.69	22307	185035.16	1.60#(1.3, 1.94)	2.02(0.67, 3.37)
Female	yes	Septicemia	Thyroid	58	81.46	50684	620846.56	0.71#(0.54, 0.92)	-0.38(-0.75, -0.01)
Female	yes	Septicemia	Lymphoma	68	47.51	16049	149746.24	1.43#(1.11, 1.81)	1.37(-0.04, 2.78)
Female	yes	Septicemia	Leukemia	0	0.53	190	2070.58	0(0, 7.01)	-2.54(-9.43, 4.35)
Female	Unknown/	Septicemia	All Sites	5518	3984.86	1494691	10858486.12	1.38#(1.35, 1.42)	1.41(1.23, 1.59)
Female	Unknown/	Septicemia	Oral Cavity and Pharynx	100	52.74	22358	156106.9	1.90#(1.54, 2.31)	3.03(1.48, 4.58)
Female	Unknown/	Septicemia	Stomach	88	32.23	22753	65213.99	2.73#(2.19, 3.36)	8.55(5.26, 11.84)
Female	Unknown/	Septicemia	Colon and Rectum	854	628.13	158955	1044263.43	1.36#(1.27, 1.45)	2.16(1.44, 2.88)
Female	Unknown/	Septicemia	Liver	45	5.04	10715	19724.82	8.92#(6.51, 11.94)	20.26(13.24, 27.28)
Female	Unknown/	Septicemia	Pancreas	101	16.4	43310	42582.07	6.16#(5.02, 7.48)	19.87(14.89, 24.85)
Female	Unknown/	Septicemia	Lung and Bronchus	377	136.14	165079	345793.11	2.77#(2.5, 3.06)	6.97(5.69, 8.25)
Female	Unknown/	Septicemia	Skin excluding Basal and Squamous	134	162.99	58202	677670.98	0.82#(0.69, 0.97)	-0.43(-0.93, 0.07)
Female	Unknown/	Septicemia	Breast	1569	1625.2	435180	4270479.55	0.97(0.92, 1.01)	-0.13(-0.39, 0.13)
Female	Unknown/	Septicemia	Cervix Uteri	143	62.82	33146	380886.83	2.28#(1.92, 2.68)	2.11(1.37, 2.85)
Female	Unknown/	Septicemia	Corpus Uteri	541	436.36	99144	1032864.05	1.24#(1.14, 1.35)	1.01(0.42, 1.6)
Female	Unknown/	Septicemia	Ovary	140	79.47	53219	308846.41	1.76#(1.48, 2.08)	1.96(1.02, 2.9)
Female	Unknown/	Septicemia	Urinary Bladder	198	133.35	29550	225723.74	1.48#(1.29, 1.71)	2.86(1.28, 4.44)
Female	Unknown/	Septicemia	Kidney and Renal Pelvis	123	70.22	29131	196825.52	1.75#(1.46, 2.09)	2.68(1.3, 4.06)
Female	Unknown/	Septicemia	Thyroid	59	84	52653	633167.76	0.70#(0.53, 0.91)	-0.39(-0.76, -0.02)
Female	Unknown/	Septicemia	Lymphoma	239	152.69	65880	494216.11	1.57#(1.37, 1.78)	1.75(0.97, 2.53)
Female	Unknown/	Septicemia	Leukemia	136	70.37	39767	223949.91	1.93#(1.62, 2.29)	2.93(1.67, 4.19)

Table S12

Septicemia SMRs and AERs by cancer type and radiotherapy status in male patients

Sex	Radiotherapy	Selected Events	Site recode ICD-O-3/WHO 2008	Observed	Expected	Persons	Person Years at Risk	SMR(95%CI)	AER(95%CI)
Male	Yes	Septicemia	All Sites	1525	1188.12	444398	2552133.36	1.28#(1.22, 1.35)	1.32(0.92, 1.72)
Male	Yes	Septicemia	Oral Cavity and Pharynx	100	41.59	30474	160219.95	2.40#(1.96, 2.92)	3.65(2.19, 5.11)
Male	Yes	Septicemia	Stomach	24	6.71	7827	18832.79	3.58#(2.29, 5.32)	9.18(3.42, 14.94)
Male	Yes	Septicemia	Colon and Rectum	95	51.69	22773	134825.17	1.84#(1.49, 2.25)	3.21(1.45, 4.97)
Male	Yes	Septicemia	Liver	8	0.99	2506	3467.94	8.10#(3.5, 15.96)	20.22(3.29, 37.15)
Male	Yes	Septicemia	Pancreas	19	2.99	6856	9718.91	6.35#(3.82, 9.91)	16.47(7.02, 25.92)
Male	Yes	Septicemia	Lung and Bronchus	178	48.25	107387	139300.36	3.69#(3.17, 4.27)	9.31(7.19, 11.43)
Male	Yes	Septicemia	Skin excluding Basal and Squamous	5	4.55	2966	10240.23	1.1(0.36, 2.57)	0.44(-5.47, 6.35)
Male	Yes	Septicemia	Prostate	717	859.94	136010	1271193.59	0.83#(0.77, 0.9)	-1.12(-1.73, -0.51)
Male	Yes	Septicemia	Urinary Bladder	33	12.4	6125	21260.49	2.66#(1.83, 3.74)	9.69(3.48, 15.9)
Male	Yes	Septicemia	Kidney and Renal Pelvis	8	2.04	4559	13985.4	3.92#(1.69, 7.73)	4.26(-0.18, 8.7)
Male	Yes	Septicemia	Thyroid	10	13.52	8066	83749.91	0.74(0.35, 1.36)	-0.42(-1.55, 0.71)
Male	Yes	Septicemia	Lymphoma	56	35.33	18823	178880.67	1.59#(1.2, 2.06)	1.16(0.11, 2.21)
Male	Yes	Septicemia	Leukemia	7	0.99	2661	24293.17	7.09#(2.85, 14.61)	2.48(0.2, 4.76)
Male	No	Septicemia	All Sites	4883	3347.91	1128608	6818087.25	1.46#(1.42, 1.5)	2.25(1.99, 2.51)
Male	No	Septicemia	Oral Cavity and Pharynx	108	57.31	19087	139519.81	1.88#(1.55, 2.28)	3.63(1.82, 5.44)
Male	No	Septicemia	Stomach	96	32.94	28824	66548.62	2.91#(2.36, 3.56)	9.48(6.14, 12.82)
Male	No	Septicemia	Colon and Rectum	760	470.71	135143	827621.71	1.61#(1.5, 1.73)	3.5(2.67, 4.33)
Male	No	Septicemia	Liver	112	11.55	27372	45602.09	9.70#(7.99, 11.67)	22.03(17.26, 26.8)
Male	No	Septicemia	Pancreas	83	11.7	37083	30711.06	7.09#(5.65, 8.79)	23.22(17.02, 29.42)
Male	No	Septicemia	Lung and Bronchus	393	101.69	124782	226394.57	3.86#(3.49, 4.27)	12.87(10.95, 14.79)
Male	No	Septicemia	Skin excluding Basal and Squamous	173	[illegible]	[illegible]	608874.84	0.89(0.77, 1.04)	-0.34(-0.96, 0.28)
Male	No	Septicemia	Prostate	1362	1614.35	302449	2523293.2	0.84#(0.8, 0.89)	-1(-1.42, -0.58)
Male	No	Septicemia	Urinary Bladder	498	354.15	75151	593319.04	1.41#(1.29, 1.54)	2.42(1.46, 3.38)
Male	No	Septicemia	Kidney and Renal Pelvis	177	99.38	42950	272003.68	1.78#(1.53, 2.06)	2.85(1.65, 4.05)
Male	No	Septicemia	Thyroid	21	17.58	8129	91123.48	1.19(0.74, 1.83)	0.38(-0.96, 1.72)
Male	No	Septicemia	Lymphoma	262	108.56	60633	375469.9	2.41#(2.13, 2.72)	4.09(3.09, 5.09)
Male	No	Septicemia	Leukemia	190	84.94	50380	263773.56	2.24#(1.93, 2.58)	3.98(2.75, 5.21)

Supplementary

Table S13

Septicemia SMRs and AERs by cancer type and radiotherapy status in female patients

Sex	Radiotherapy	Selected Events	Site recode ICD-O-3/WHO 2008	Observed	Expected	Persons	Person Years at Risk	SMR(95%CI)	AER(95%CI)
Female	Yes	Septicemia	All Sites	1385	1018.81	451889	3349526.27	1.36#(1.29, 1.43)	1.09(0.8, 1.38)
Female	Yes	Septicemia	Oral Cavity and Pharynx	46	19.35	11690	69470.83	2.38#(1.74, 3.17)	3.84(1.56, 6.12)
Female	Yes	Septicemia	Stomach	9	2.41	2903	7619.82	3.73#(1.71, 7.08)	8.64(-0.04, 17.32)
Female	Yes	Septicemia	Colon and Rectum	65	35.64	15347	95200.21	1.82#(1.41, 2.32)	3.08(1.02, 5.14)
Female	Yes	Septicemia	Liver	3	0.3	755	1166.04	9.99#(2.06, 29.21)	23.15(-7.35, 53.65)
Female	Yes	Septicemia	Pancreas	17	2.43	5950	8924.01	7.01#(4.08, 11.22)	16.33(6.66, 26)
Female	Yes	Septicemia	Lung and Bronchus	132	35.6	68417	109834.13	3.71#(3.1, 4.4)	8.78(6.47, 11.09)
Female	Yes	Septicemia	Skin excluding Basal and Squamous	7	3.43	1524	7673.44	2.04(0.82, 4.21)	4.66(-3.59, 12.91)
Female	Yes	Septicemia	Breast	525	610.09	199290	1890062.8	0.86#(0.79, 0.94)	-0.45(-0.8, -0.1)
Female	Yes	Septicemia	Cervix Uteri	98	30.85	16993	132893.59	3.18#(2.58, 3.87)	5.05(3.38, 6.72)
Female	Yes	Septicemia	Corpus Uteri	208	142.16	31488	315092.44	1.46#(1.27, 1.68)	2.09(0.93, 3.25)
Female	Yes	Septicemia	Ovary	10	3.82	2034	17140.91	2.62#(1.26, 4.81)	3.61(-0.64, 7.86)
Female	Yes	Septicemia	Urinary Bladder	20	5.33	2663	8501.24	3.75#(2.29, 5.8)	17.26(5.67, 28.85)
Female	Yes	Septicemia	Kidney and Renal Pelvis	3	0.84	2351	9932.75	3.58(0.74, 10.45)	2.18(-1.69, 6.05)
Female	Yes	Septicemia	Thyroid	16	28.79	22581	264313.64	0.56#(0.32, 0.9)	-0.48(-0.98, 0.02)
Female	Yes	Septicemia	Lymphoma	56	37.77	15959	161950.8	1.48#(1.12, 1.93)	1.13(-0.04, 2.3)
Female	Yes	Septicemia	Leukemia	6	0.86	1892	19640.81	6.95#(2.55, 15.12)	2.62(0.01, 5.23)
Female	No	Septicemia	All Sites	4133	2966.05	1042802	7508959.86	1.39#(1.35, 1.44)	1.55(1.33, 1.77)
Female	No	Septicemia	Oral Cavity and Pharynx	54	33.39	10668	86636.07	1.62#(1.22, 2.11)	2.38(0.27, 4.49)
Female	No	Septicemia	Stomach	79	29.82	19850	57594.17	2.65#(2.1, 3.3)	8.54(4.99, 12.09)
Female	No	Septicemia	Colon and Rectum	789	592.49	143608	949063.23	1.33#(1.24, 1.43)	2.07(1.3, 2.84)
Female	No	Septicemia	Liver	42	4.74	9960	18558.77	8.86#(6.38, 11.97)	20.08(12.87, 27.29)
Female	No	Septicemia	Pancreas	84	13.97	37360	33658.07	6.01#(4.79, 7.44)	20.81(15.05, 26.57)
Female	No	Septicemia	Lung and Bronchus	245	100.53	96662	235958.98	2.44#(2.14, 2.76)	6.12(4.58, 7.66)
Female	No	Septicemia	Skin excluding Basal and Squamous	127	159.57	56678	669997.54	0.80#(0.66, 0.95)	-0.49(-0.99, 0.01)
Female	No	Septicemia	Breast	1044	1015.11	235890	2380416.75	1.03(0.97, 1.09)	0.12(-0.25, 0.49)
Female	No	Septicemia	Cervix Uteri	45	31.97	16153	247993.24	1.41#(1.03, 1.88)	0.53(-0.16, 1.22)
Female	No	Septicemia	Corpus Uteri	333	294.2	67656	717771.61	1.13#(1.01, 1.26)	0.54(-0.14, 1.22)
Female	No	Septicemia	Ovary	130	75.65	51185	291705.5	1.72#(1.44, 2.04)	1.86(0.9, 2.82)
Female	No	Septicemia	Urinary Bladder	178	128.02	26887	217222.5	1.39#(1.19, 1.61)	2.3(0.72, 3.88)
Female	No	Septicemia	Kidney and Renal Pelvis	120	69.38	26780	186892.77	1.73#(1.43, 2.07)	2.71(1.27, 4.15)
Female	No	Septicemia	Thyroid	43	55.21	30072	368854.12	0.78(0.56, 1.05)	-0.33(-0.86, 0.2)
Female	No	Septicemia	Lymphoma	183	114.92	49921	332265.31	1.59#(1.37, 1.84)	2.05(1.03, 3.07)
Female	No	Septicemia	Leukemia	130	69.5	37875	204309.1	1.87#(1.56, 2.22)	2.96(1.61, 4.31)

Table S14

Septicemia SMRs and AERs by cancer type and chemotherapy status in male patients

Sex	Chemotherapy recode (yes, no/unk)	Selected Events	Site recode ICD-O-3/WHO 2008	Observed	Expected	Persons	Person Years at Risk	SMR(95%CI)	AER(95%CI)
Male	No/Unknown	Septicemia	All Sites	5297	4141.74	1195157	7851449.43	1.28#(1.24, 1.31)	1.47(1.23, 1.71)
Male	No/Unknown	Septicemia	Oral Cavity and Pharynx	167	83	33861	224505.87	2.01#(1.72, 2.34)	3.74(2.36, 5.12)
Male	No/Unknown	Septicemia	Stomach	86	30.11	22706	55630.43	2.86#(2.28, 3.53)	10.05(6.26, 13.84)
Male	No/Unknown	Septicemia	Colon and Rectum	699	425.26	108724	704842.09	1.64#(1.52, 1.77)	3.88(2.95, 4.81)
Male	No/Unknown	Septicemia	Liver	87	7.86	20331	27992.45	11.07#(8.87, 13.66)	28.27(21.46, 35.08)
Male	No/Unknown	Septicemia	Pancreas	61	8.65	26032	20046.29	7.06#(5.4, 9.06)	26.12(17.97, 34.27)
Male	No/Unknown	Septicemia	Lung and Bronchus	425	114.25	150845	252617.81	3.72#(3.37, 4.09)	12.3(10.5, 14.1)
Male	No/Unknown	Septicemia	Skin excluding Basal and Squamous	173	196.6	63206	612025.91	0.88(0.75, 1.02)	-0.39(-1.01, 0.23)
Male	No/Unknown	Septicemia	Prostate	2060	2465.45	434354	3776501.93	0.84#(0.8, 0.87)	-1.07(-1.42, -0.72)
Male	No/Unknown	Septicemia	Urinary Bladder	452	325.49	66445	539436.08	1.39#(1.26, 1.52)	2.35(1.34, 3.36)
Male	No/Unknown	Septicemia	Kidney and Renal Pelvis	175	99.02	42481	264298.21	1.77#(1.52, 2.05)	2.87(1.64, 4.1)
Male	No/Unknown	Septicemia	Thyroid	31	30.88	15847	173758.03	1(0.68, 1.42)	0.01(-0.88, 0.9)
Male	No/Unknown	Septicemia	Lymphoma	131	62.43	28788	197295.71	2.10#(1.75, 2.49)	3.48(2.1, 4.86)
Male	No/Unknown	Septicemia	Leukemia	101	61.29	23814	118592.33	1.65#(1.34, 2)	3.35(1.25, 5.45)
Male	Yes	Septicemia	All Sites	1111	394.29	377849	1518771.19	2.82#(2.65, 2.99)	4.72(4.22, 5.22)
Male	Yes	Septicemia	Oral Cavity and Pharynx	41	15.91	15700	75233.89	2.58#(1.85, 3.5)	3.34(1.38, 5.3)
Male	Yes	Septicemia	Stomach	34	9.54	13945	29750.99	3.56#(2.47, 4.98)	8.22(3.88, 12.56)
Male	Yes	Septicemia	Colon and Rectum	156	97.15	49192	257604.79	1.61#(1.36, 1.88)	2.28(1.07, 3.49)
Male	Yes	Septicemia	Liver	33	4.68	9547	21077.58	7.06#(4.86, 9.91)	13.44(7.74, 19.14)
Male	Yes	Septicemia	Pancreas	41	6.05	17907	20383.68	6.78#(4.87, 9.2)	17.15(10.56, 23.74)
Male	Yes	Septicemia	Lung and Bronchus	146	35.69	81324	113077.12	4.09#(3.45, 4.81)	9.76(7.42, 12.1)
Male	Yes	Septicemia	Skin excluding Basal and Squamous	5	1.61	1890	7089.16	3.10#(1.01, 7.24)	4.78(-2.33, 11.89)
Male	Yes	Septicemia	Prostate	19	8.84	4105	17984.86	2.15#(1.29, 3.36)	5.65(-0.1, 11.4)
Male	Yes	Septicemia	Urinary Bladder	79	41.06	14831	75143.45	1.92#(1.52, 2.4)	5.05(2.19, 7.91)
Male	Yes	Septicemia	Kidney and Renal Pelvis	10	2.4	5028	21690.87	4.17#(2, 7.67)	3.5(0.32, 6.68)
Male	Yes	Septicemia	Thyroid	0	0.22	348	1115.35	0(0, 16.81)	-1.97(-10.21, 6.27)
Male	Yes	Septicemia	Lymphoma	187	81.46	50668	357054.86	2.30#(1.98, 2.65)	2.96(2.06, 3.86)
Male	Yes	Septicemia	Leukemia	96	24.64	29227	169474.4	3.90#(3.16, 4.76)	4.21(2.94, 5.48)

Table S15

Septicemia SMRs and AERs by cancer type and chemotherapy status in female patients

Sex	Chemotherapy recode (yes, no/unk)	Selected Events	Site recode ICD-O-3/WHO 2008	Observed	Expected	Persons	Person Years at Risk	SMR(95%CI)	AER(95%CI)
Female	No/Unknown	Septicemia	All Sites	4299	3361.31	1035070	8089548.4	1.28#(1.24, 1.32)	1.16(0.95, 1.37)
Female	No/Unknown	Septicemia	Oral Cavity and Pharynx	84	48.92	18243	136442.85	1.72#(1.37, 2.13)	2.57(0.91, 4.23)
Female	No/Unknown	Septicemia	Stomach	67	27.92	16045	50045.83	2.40#(1.86, 3.05)	7.81(4, 11.62)
Female	No/Unknown	Septicemia	Colon and Rectum	720	539.9	118679	812841.4	1.33#(1.24, 1.43)	2.22(1.36, 3.08)
Female	No/Unknown	Septicemia	Liver	29	3.64	7706	11691.97	7.96#(5.33, 11.43)	21.69(12.12, 31.26)
Female	No/Unknown	Septicemia	Pancreas	56	11.2	28498	24168.96	5.00#(3.78, 6.49)	18.54(11.9, 25.18)
Female	No/Unknown	Septicemia	Lung and Bronchus	268	107.09	105104	240471.76	2.50#(2.21, 2.82)	6.69(5.11, 8.27)
Female	No/Unknown	Septicemia	Skin excluding Basal and Squamous	126	161.69	57236	672169.78	0.78#(0.65, 0.93)	-0.53(-1.02, -0.04)
Female	No/Unknown	Septicemia	Breast	1249	1370.49	286697	2883993.3	0.91#(0.86, 0.96)	-0.42(-0.77, -0.07)
Female	No/Unknown	Septicemia	Cervix Uteri	110	56.76	25043	337255.09	1.94#(1.59, 2.34)	1.58(0.83, 2.33)
Female	No/Unknown	Septicemia	Corpus Uteri	499	419.68	86309	975050.65	1.19#(1.09, 1.3)	0.81(0.2, 1.42)
Female	No/Unknown	Septicemia	Ovary	55	36.92	19307	133131.32	1.49#(1.12, 1.94)	1.36(-0.05, 2.77)
Female	No/Unknown	Septicemia	Urinary Bladder	172	120.17	24469	200175.06	1.43#(1.23, 1.66)	2.59(0.92, 4.26)
Female	No/Unknown	Septicemia	Kidney and Renal Pelvis	119	69.06	26405	178565.6	1.72#(1.43, 2.06)	2.8(1.3, 4.3)
Female	No/Unknown	Septicemia	Thyroid	59	83.62	52238	630974.66	0.71#(0.54, 0.91)	-0.39(-0.76, -0.02)
Female	No/Unknown	Septicemia	Lymphoma	121	73.7	27053	197788	1.64#(1.36, 1.96)	2.39(1.01, 3.77)
Female	No/Unknown	Septicemia	Leukemia	76	52.75	18883	93330.33	1.44#(1.14, 1.8)	2.49(0.11, 4.87)
Female	Yes	Septicemia	All Sites	1219	623.56	459621	2768937.72	1.95#(1.85, 2.07)	2.15(1.85, 2.45)
Female	Yes	Septicemia	Oral Cavity and Pharynx	16	3.82	4115	19664.04	4.19#(2.39, 6.8)	6.19(1.75, 10.63)
Female	Yes	Septicemia	Stomach	21	4.31	6708	15168.16	4.87#(3.02, 7.45)	11(4.5, 17.5)
Female	Yes	Septicemia	Colon and Rectum	134	88.23	40276	231422.03	1.52#(1.27, 1.8)	1.98(0.72, 3.24)
Female	Yes	Septicemia	Liver	16	1.4	3009	8032.84	11.42#(6.53, 18.55)	18.17(8, 28.34)
Female	Yes	Septicemia	Pancreas	45	5.2	14812	18413.11	8.66#(6.32, 11.59)	21.62(14.09, 29.15)
Female	Yes	Septicemia	Lung and Bronchus	109	29.05	59975	105321.35	3.75#(3.08, 4.53)	7.59(5.4, 9.78)
Female	Yes	Septicemia	Skin excluding Basal and Squamous	8	1.3	966	5501.2	6.14#(2.65, 12.1)	12.17(1.31, 23.03)
Female	Yes	Septicemia	Breast	320	254.71	148483	1386486.26	1.26#(1.12, 1.4)	0.47(0.13, 0.81)
Female	Yes	Septicemia	Cervix Uteri	33	6.06	8103	43631.74	5.45#(3.75, 7.65)	6.18(3.37, 8.99)
Female	Yes	Septicemia	Corpus Uteri	42	16.67	12835	57813.4	2.52#(1.82, 3.41)	4.38(1.78, 6.98)
Female	Yes	Septicemia	Ovary	85	42.55	33912	175715.1	2.00#(1.6, 2.47)	2.42(1.16, 3.68)
Female	Yes	Septicemia	Urinary Bladder	26	13.18	5081	25548.67	1.97#(1.29, 2.89)	5.02(0.22, 9.82)
Female	Yes	Septicemia	Kidney and Renal Pelvis	4	1.17	2726	18259.91	3.43(0.93, 8.79)	1.55(-0.89, 3.99)
Female	Yes	Septicemia	Thyroid	0	0.38	415	2193.1	0(0, 9.8)	-1.72(-7.23, 3.79)
Female	Yes	Septicemia	Lymphoma	118	78.99	38827	296428.11	1.49#(1.24, 1.79)	1.32(0.39, 2.25)
Female	Yes	Septicemia	Leukemia	60	17.62	20884	130619.58	3.41#(2.6, 4.38)	3.24(1.92, 4.56)

Table S16

Septicemia SMRs and AERs by cancer type and race among male patients

Sex	Race recode (White, Black, Other)	Selected Events	Site recode ICD-O-3/WHO 2008	Observed	Expected	Persons	Person Years at Risk	SMR(95%CI)	AER(95%CI)
Male	All races	Septicemia	All Sites	6408	4536.03	1573006	9370220.61	1.41#(1.38, 1.45)	2(1.78, 2.22)
Male	All races	Septicemia	Oral Cavity and Pharynx	208	98.91	49561	299739.76	2.10#(1.83, 2.41)	3.64(2.49, 4.79)
Male	All races	Septicemia	Stomach	120	39.65	36651	85381.42	3.03#(2.51, 3.62)	9.41(6.51, 12.31)
Male	All races	Septicemia	Colon and Rectum	855	522.41	157916	962446.88	1.64#(1.53, 1.75)	3.46(2.7, 4.22)
Male	All races	Septicemia	Liver	120	12.54	29878	49070.03	9.57#(7.94, 11.45)	21.9(17.31, 26.49)
Male	All races	Septicemia	Pancreas	102	14.69	43939	40429.97	6.94#(5.66, 8.43)	21.59(16.36, 26.82)
Male	All races	Septicemia	Lung and Bronchus	571	149.94	232169	365694.93	3.81#(3.5, 4.13)	11.51(10.07, 12.95)
Male	All races	Septicemia	Skin excluding Basal and Squamous	178	198.21	65096	619115.07	0.9(0.77, 1.04)	-0.33(-0.94, 0.28)
Male	All races	Septicemia	Prostate	2079	2474.29	438459	3794486.79	0.84#(0.8, 0.88)	-1.04(-1.39, -0.69)
Male	All races	Septicemia	Urinary Bladder	531	366.55	81276	614579.53	1.45#(1.33, 1.58)	2.68(1.72, 3.64)
Male	All races	Septicemia	Kidney and Renal Pelvis	185	101.42	47509	285989.08	1.82#(1.57, 2.11)	2.92(1.76, 4.08)
Male	All races	Septicemia	Thyroid	31	31.1	16195	174873.39	1(0.68, 1.41)	-0.01(-0.89, 0.87)
Male	All races	Septicemia	Lymphoma	318	143.89	79456	554350.57	2.21#(1.97, 2.47)	3.14(2.38, 3.9)
Male	All races	Septicemia	Leukemia	197	85.92	53041	288066.73	2.29#(1.98, 2.64)	3.86(2.72, 5)
Male	White	Septicemia	All Sites	5143	3846.91	1319602	8054795.36	1.34#(1.3, 1.37)	1.61(1.38, 1.84)
Male	White	Septicemia	Oral Cavity and Pharynx	185	88.13	41263	256538.34	2.10#(1.81, 2.42)	3.78(2.52, 5.04)
Male	White	Septicemia	Stomach	86	27.42	26968	58633.24	3.14#(2.51, 3.87)	9.99(6.43, 13.55)
Male	White	Septicemia	Colon and Rectum	679	444.07	130572	800585.7	1.53#(1.42, 1.65)	2.93(2.11, 3.75)
Male	White	Septicemia	Liver	77	7.9	19390	30693.74	9.75#(7.69, 12.18)	22.51(16.63, 28.39)
Male	White	Septicemia	Pancreas	68	12.13	36203	33196.64	5.61#(4.35, 7.11)	16.83(11.55, 22.11)
Male	White	Septicemia	Lung and Bronchus	462	122.65	193441	304137.55	3.77#(3.43, 4.13)	11.16(9.6, 12.72)
Male	White	Septicemia	Skin excluding Basal and Squamous	172	194.74	63518	604610.21	0.88(0.76, 1.03)	-0.38(-1, 0.24)
Male	White	Septicemia	Prostate	1631	2035.76	363329	3195126.32	0.80#(0.76, 0.84)	-1.27(-1.64, -0.9)
Male	White	Septicemia	Urinary Bladder	473	341.31	74384	568840.59	1.39#(1.26, 1.52)	2.32(1.34, 3.3)
Male	White	Septicemia	Kidney and Renal Pelvis	145	86.39	39780	242844.78	1.68#(1.42, 1.98)	2.41(1.18, 3.64)
Male	White	Septicemia	Thyroid	23	26.92	13618	149694.65	0.85(0.54, 1.28)	-0.26(-1.19, 0.67)
Male	White	Septicemia	Lymphoma	264	127.17	67577	481588.06	2.08#(1.83, 2.34)	2.84(2.04, 3.64)
Male	White	Septicemia	Leukemia	163	78.75	45998	253039.39	2.07#(1.76, 2.41)	3.33(2.13, 4.53)
Male	Black	Septicemia	All Sites	735	469.88	122869	633534.75	1.56#(1.45, 1.68)	4.18(3.11, 5.25)
Male	Black	Septicemia	Oral Cavity and Pharynx	9	5.85	3607	13765.06	1.54(0.7, 2.92)	2.29(-3.2, 7.78)
Male	Black	Septicemia	Stomach	17	5.28	3280	7362.69	3.22#(1.88, 5.16)	15.92(3.37, 28.47)
Male	Black	Septicemia	Colon and Rectum	75	42.66	10607	54845.83	1.76#(1.38, 2.2)	5.9(2.03, 9.77)
Male	Black	Septicemia	Liver	21	2.14	3275	4572.22	9.80#(6.07, 14.98)	41.24(20.66, 61.82)
Male	Black	Septicemia	Pancreas	16	1.59	3482	3201.79	10.06#(5.75, 16.33)	45(19.38, 70.62)
Male	Black	Septicemia	Lung and Bronchus	64	17.48	18991	27959.46	3.66#(2.82, 4.67)	16.64(10.32, 22.96)
Male	Black	Septicemia	Skin excluding Basal and Squamous	3	1.85	502	5273.31	1.62(0.33, 4.74)	2.18(-6, 10.36)
Male	Black	Septicemia	Prostate	300	323.76	45005	351320.37	0.93(0.82, 1.04)	-0.68(-2.07, 0.71)
Male	Black	Septicemia	Urinary Bladder	32	13.24	2616	15527.09	2.42#(1.65, 3.41)	12.08(3.6, 20.56)
Male	Black	Septicemia	Kidney and Renal Pelvis	28	10.19	3603	20277.91	2.75#(1.83, 3.97)	8.78(2.81, 14.75)
Male	Black	Septicemia	Thyroid	2	1.76	590	5277.75	1.13(0.14, 4.1)	0.45(-6.75, 7.65)
Male	Black	Septicemia	Lymphoma	32	9.71	5389	34698.16	3.30#(2.25, 4.65)	6.42(2.77, 10.07)
Male	Black	Septicemia	Leukemia	20	5.06	2939	14104.19	3.95#(2.41, 6.1)	10.59(3.64, 17.54)
Male	Other (American Indian/AK Native, A	Septicemia	All Sites	530	219.24	130535	681890.5	2.42#(2.22, 2.63)	4.56(3.77, 5.35)
Male	Other (American Indian/AK Native, A	Septicemia	Oral Cavity and Pharynx	14	4.92	4691	29436.36	2.84#(1.56, 4.77)	3.08(0.18, 5.98)
Male	Other (American Indian/AK Native, A	Septicemia	Stomach	17	6.95	6403	19385.48	2.45#(1.43, 3.92)	5.19(0.24, 10.14)
Male	Other (American Indian/AK Native, A	Septicemia	Colon and Rectum	101	35.68	16737	107015.36	2.83#(2.31, 3.44)	6.1(3.96, 8.24)
Male	Other (American Indian/AK Native, A	Septicemia	Liver	22	2.49	7213	13804.06	8.82#(5.53, 13.35)	14.13(7.11, 21.15)
Male	Other (American Indian/AK Native, A	Septicemia	Pancreas	18	0.97	4254	4031.55	18.48#(10.95, 29.2)	42.23(21.1, 63.36)
Male	Other (American Indian/AK Native, A	Septicemia	Lung and Bronchus	45	9.8	19737	33597.92	4.59#(3.35, 6.14)	10.48(6.16, 14.8)
Male	Other (American Indian/AK Native, A	Septicemia	Skin excluding Basal and Squamous	3	1.62	1076	9231.55	1.85(0.38, 5.4)	1.49(-3.07, 6.05)
Male	Other (American Indian/AK Native, A	Septicemia	Prostate	148	114.77	30125	248040.1	1.29#(1.09, 1.51)	1.34(0.06, 2.62)
Male	Other (American Indian/AK Native, A	Septicemia	Urinary Bladder	26	12.01	4276	30211.85	2.17#(1.41, 3.17)	4.63(0.63, 8.63)
Male	Other (American Indian/AK Native, A	Septicemia	Kidney and Renal Pelvis	12	4.84	4126	22866.39	2.48#(1.28, 4.33)	3.13(-0.39, 6.65)
Male	Other (American Indian/AK Native, A	Septicemia	Thyroid	6	2.42	1987	19900.98	2.48(0.91, 5.4)	1.8(-1.06, 4.66)
Male	Other (American Indian/AK Native, A	Septicemia	Lymphoma	22	7.01	6490	38064.35	3.14#(1.97, 4.75)	3.94(1.17, 6.71)
Male	Other (American Indian/AK Native, A	Septicemia	Leukemia	14	2.11	4104	20923.16	6.62#(3.62, 11.11)	5.68(1.92, 9.44)

Table S17

Septicemia SMRs and AERs by cancer type and race among female patients

Sex	Race recode (White, Black, Other)	Selected Events	Site recode ICD-O-3/WHO 2008	Observed	Expected	Persons	Person Years at Risk	SMR(95%CI)	AER(95%CI)
Female	All races	Septicemia	All Sites	5518	3984.86	1494691	10858486.12	1.38#(1.35, 1.42)	1.41(1.23, 1.59)
Female	All races	Septicemia	Oral Cavity and Pharynx	100	52.74	22358	156106.9	1.90#(1.54, 2.31)	3.03(1.48, 4.58)
Female	All races	Septicemia	Stomach	88	32.23	22753	65213.99	2.73#(2.19, 3.36)	8.55(5.26, 11.84)
Female	All races	Septicemia	Colon and Rectum	854	628.13	158955	1044263.43	1.36#(1.27, 1.45)	2.16(1.44, 2.88)
Female	All races	Septicemia	Liver	45	5.04	10715	19724.82	8.92#(6.51, 11.94)	20.26(13.24, 27.28)
Female	All races	Septicemia	Pancreas	101	16.4	43310	42582.07	6.16#(5.02, 7.48)	19.87(14.89, 24.85)
Female	All races	Septicemia	Lung and Bronchus	377	136.14	165079	345793.11	2.77#(2.5, 3.06)	6.97(5.69, 8.25)
Female	All races	Septicemia	Skin excluding Basal and Squamous	134	162.99	58202	677670.98	0.82#(0.69, 0.97)	-0.43(-0.93, 0.07)
Female	All races	Septicemia	Breast	1569	1625.2	435180	4270479.55	0.97(0.92, 1.01)	-0.13(-0.39, 0.13)
Female	All races	Septicemia	Cervix Uteri	143	62.82	33146	380886.83	2.28#(1.92, 2.68)	2.11(1.37, 2.85)
Female	All races	Septicemia	Corpus Uteri	541	436.36	99144	1032864.05	1.24#(1.14, 1.35)	1.01(0.42, 1.6)
Female	All races	Septicemia	Ovary	140	79.47	53219	308846.41	1.76#(1.48, 2.08)	1.96(1.02, 2.9)
Female	All races	Septicemia	Urinary Bladder	198	133.35	29550	225723.74	1.48#(1.29, 1.71)	2.86(1.28, 4.44)
Female	All races	Septicemia	Kidney and Renal Pelvis	123	70.22	29131	196825.52	1.75#(1.46, 2.09)	2.68(1.3, 4.06)
Female	All races	Septicemia	Thyroid	59	84	52653	633167.76	0.70#(0.53, 0.91)	-0.39(-0.76, -0.02)
Female	All races	Septicemia	Lymphoma	239	152.69	65880	494216.11	1.57#(1.37, 1.78)	1.75(0.97, 2.53)
Female	All races	Septicemia	Leukemia	136	70.37	39767	223949.91	1.93#(1.62, 2.29)	2.93(1.67, 4.19)
Female	White	Septicemia	All Sites	4421	3497.07	1250120	9276889.34	1.26#(1.23, 1.3)	1(0.81, 1.19)
Female	White	Septicemia	Oral Cavity and Pharynx	79	46.1	18335	127654.78	1.71#(1.36, 2.14)	2.58(0.86, 4.3)
Female	White	Septicemia	Stomach	58	22.29	15874	42300.45	2.60#(1.98, 3.36)	8.44(4.29, 12.59)
Female	White	Septicemia	Colon and Rectum	678	540.6	132592	871366.28	1.25#(1.16, 1.35)	1.58(0.8, 2.36)
Female	White	Septicemia	Liver	24	3.26	6843	12332.17	7.36#(4.72, 10.95)	16.82(8.53, 25.11)
Female	White	Septicemia	Pancreas	70	13.08	35253	33566.24	5.35#(4.17, 6.76)	16.96(11.64, 22.28)
Female	White	Septicemia	Lung and Bronchus	305	116.12	140478	294463.13	2.63#(2.34, 2.94)	6.41(5.04, 7.78)
Female	White	Septicemia	Skin excluding Basal and Squamous	131	159.49	56530	659681.04	0.82#(0.69, 0.97)	-0.43(-0.94, 0.08)
Female	White	Septicemia	Breast	1302	1428.05	361507	3634496.81	0.91#(0.86, 0.96)	-0.35(-0.63, -0.07)
Female	White	Septicemia	Cervix Uteri	95	45.64	25299	301858.84	2.08#(1.68, 2.54)	1.64(0.87, 2.41)
Female	White	Septicemia	Corpus Uteri	460	399.7	83260	906744.58	1.15#(1.05, 1.26)	0.66(0.03, 1.29)
Female	White	Septicemia	Ovary	112	70.51	45537	262086	1.59#(1.31, 1.91)	1.58(0.57, 2.59)
Female	White	Septicemia	Urinary Bladder	172	122.04	26443	207579.36	1.41#(1.21, 1.64)	2.41(0.79, 4.03)
Female	White	Septicemia	Kidney and Renal Pelvis	88	59.74	24197	163924.91	1.47#(1.18, 1.81)	1.72(0.27, 3.17)
Female	White	Septicemia	Thyroid	42	70.2	42159	518337.79	0.60#(0.43, 0.81)	-0.54(-0.94, -0.14)
Female	White	Septicemia	Lymphoma	194	136.23	56139	428831.23	1.42#(1.23, 1.64)	1.35(0.52, 2.18)
Female	White	Septicemia	Leukemia	110	64.54	34361	195517.75	1.70#(1.4, 2.05)	2.32(1, 3.64)
Female	Black	Septicemia	All Sites	638	312.43	106850	621972.44	2.04#(1.89, 2.21)	5.23(4.26, 6.2)
Female	Black	Septicemia	Oral Cavity and Pharynx	12	3.61	1519	9454.76	3.32#(1.72, 5.8)	8.87(0.68, 17.06)
Female	Black	Septicemia	Stomach	10	5	2276	6934.27	2(0.96, 3.68)	7.21(-3.73, 18.15)
Female	Black	Septicemia	Colon and Rectum	106	56.14	12055	71477.79	1.89#(1.55, 2.28)	6.98(3.49, 10.47)
Female	Black	Septicemia	Liver	8	0.61	1069	1627.73	13.12#(5.66, 25.85)	45.4(10.15, 80.65)
Female	Black	Septicemia	Pancreas	17	2.26	3870	4146.46	7.53#(4.39, 12.06)	35.56(14.85, 56.27)
Female	Black	Septicemia	Lung and Bronchus	44	13.1	11720	22791.43	3.36#(2.44, 4.51)	13.56(7.07, 20.05)
Female	Black	Septicemia	Skin excluding Basal and Squamous	2	2.03	603	7147.5	0.99(0.12, 3.56)	-0.04(-5.54, 5.46)
Female	Black	Septicemia	Breast	161	128.12	32156	252399.01	1.26#(1.07, 1.47)	1.3(-0.02, 2.62)
Female	Black	Septicemia	Cervix Uteri	31	12.03	3681	34986.54	2.58#(1.75, 3.66)	5.42(1.75, 9.09)
Female	Black	Septicemia	Corpus Uteri	46	21.83	6114	37062.3	2.11#(1.54, 2.81)	6.52(2.17, 10.87)
Female	Black	Septicemia	Ovary	13	5.26	2992	15461.09	2.47#(1.31, 4.22)	5(-0.42, 10.42)
Female	Black	Septicemia	Urinary Bladder	15	7.08	1515	7330.93	2.12#(1.19, 3.5)	10.81(-1.74, 23.36)
Female	Black	Septicemia	Kidney and Renal Pelvis	21	7.43	2495	17212.93	2.83#(1.75, 4.32)	7.88(1.81, 13.95)
Female	Black	Septicemia	Thyroid	4	6.84	2825	29150.05	0.58(0.16, 1.5)	-0.97(-3.18, 1.24)
Female	Black	Septicemia	Lymphoma	24	10.27	4222	30259.67	2.34#(1.5, 3.48)	4.54(0.75, 8.33)
Female	Black	Septicemia	Leukemia	18	4.39	2438	12234.27	4.10#(2.43, 6.49)	11.13(3.55, 18.71)
Female	Other (American Indian/AK Native, Asian/Pacific Islander)	Septicemia	All Sites	459	175.37	137721	959624.34	2.62#(2.38, 2.87)	2.96(2.45, 3.47)
Female	Other (American Indian/AK Native, Asian/Pacific Islander)	Septicemia	Oral Cavity and Pharynx	9	3.02	2504	18997.35	2.98#(1.36, 5.65)	3.15(-0.43, 6.73)
Female	Other (American Indian/AK Native, Asian/Pacific Islander)	Septicemia	Stomach	20	4.94	4603	15979.27	4.05#(2.47, 6.25)	9.42(3.3, 15.54)
Female	Other (American Indian/AK Native, Asian/Pacific Islander)	Septicemia	Colon and Rectum	70	31.39	14308	101419.36	2.23#(1.74, 2.82)	3.81(1.86, 5.76)
Female	Other (American Indian/AK Native, Asian/Pacific Islander)	Septicemia	Liver	13	1.17	2803	5764.91	11.09#(5.9, 18.96)	20.52(7.74, 33.3)
Female	Other (American Indian/AK Native, Asian/Pacific Islander)	Septicemia	Pancreas	14	1.06	4187	4869.37	13.18#(7.2, 22.11)	26.57(10.97, 42.17)
Female	Other (American Indian/AK Native, Asian/Pacific Islander)	Septicemia	Lung and Bronchus	28	6.92	12881	28538.55	4.05#(2.69, 5.85)	7.39(3.33, 11.45)
Female	Other (American Indian/AK Native, Asian/Pacific Islander)	Septicemia	Skin excluding Basal and Squamous	1	1.47	1069	10842.44	0.68(0.02, 3.78)	-0.44(-3.28, 2.4)
Female	Other (American Indian/AK Native, Asian/Pacific Islander)	Septicemia	Breast	106	69.02	41517	383583.73	1.54#(1.26, 1.86)	0.96(0.28, 1.64)
Female	Other (American Indian/AK Native, Asian/Pacific Islander)	Septicemia	Cervix Uteri	17	5.15	4166	44041.45	3.30#(1.92, 5.29)	2.69(0.6, 4.78)
Female	Other (American Indian/AK Native, Asian/Pacific Islander)	Septicemia	Corpus Uteri	35	14.83	9770	89057.17	2.36#(1.64, 3.28)	2.27(0.72, 3.82)
Female	Other (American Indian/AK Native, Asian/Pacific Islander)	Septicemia	Ovary	15	3.7	4690	31299.36	4.05#(2.27, 6.68)	3.61(0.9, 6.32)
Female	Other (American Indian/AK Native, Asian/Pacific Islander)	Septicemia	Urinary Bladder	11	4.23	1592	10813.44	2.60#(1.3, 4.66)	6.26(-0.81, 13.33)
Female	Other (American Indian/AK Native, Asian/Pacific Islander)	Septicemia	Kidney and Renal Pelvis	14	3.05	2439	15687.68	4.59#(2.51, 7.71)	6.98(1.82, 12.14)
Female	Other (American Indian/AK Native, Asian/Pacific Islander)	Septicemia	Thyroid	13	6.96	7669	85679.91	1.87(0.99, 3.2)	0.71(-0.31, 1.73)
Female	Other (American Indian/AK Native, Asian/Pacific Islander)	Septicemia	Lymphoma	21	6.2	5519	35125.21	3.39#(2.1, 5.18)	4.21(1.3, 7.12)
Female	Other (American Indian/AK Native, Asian/Pacific Islander)	Septicemia	Leukemia	8	1.44	2968	16197.89	5.55#(2.4, 10.94)	4.05(0.33, 7.77)
Female	Unknown	Septicemia	All Sites	0	0	0	0	0(0, 0)	#DIV/0!
Female	Unknown	Septicemia	Oral Cavity and Pharynx	0	0	0	0	0(0, 0)	#DIV/0!
Female	Unknown	Septicemia	Stomach	0	0	0	0	0(0, 0)	#DIV/0!
Female	Unknown	Septicemia	Colon and Rectum	0	0	0	0	0(0, 0)	#DIV/0!
Female	Unknown	Septicemia	Liver	0	0	0	0	0(0, 0)	#DIV/0!
Female	Unknown	Septicemia	Pancreas	0	0	0	0	0(0, 0)	#DIV/0!
Female	Unknown	Septicemia	Lung and Bronchus	0	0	0	0	0(0, 0)	#DIV/0!
Female	Unknown	Septicemia	Skin excluding Basal and Squamous	0	0	0	0	0(0, 0)	#DIV/0!
Female	Unknown	Septicemia	Breast	0	0	0	0	0(0, 0)	#DIV/0!
Female	Unknown	Septicemia	Cervix Uteri	0	0	0	0	0(0, 0)	#DIV/0!
Female	Unknown	Septicemia	Corpus Uteri	0	0	0	0	0(0, 0)	#DIV/0!
Female	Unknown	Septicemia	Ovary	0	0	0	0	0(0, 0)	#DIV/0!
Female	Unknown	Septicemia	Urinary Bladder	0	0	0	0	0(0, 0)	#DIV/0!
Female	Unknown	Septicemia	Kidney and Renal Pelvis	0	0	0	0	0(0, 0)	#DIV/0!
Female	Unknown	Septicemia	Thyroid	0	0	0	0	0(0, 0)	#DIV/0!
Female	Unknown	Septicemia	Lymphoma	0	0	0	0	0(0, 0)	#DIV/0!
Female	Unknown	Septicemia	Leukemia	0	0	0	0	0(0, 0)	#DIV/0!

Table S18

Septicemia SMRs and AERs by marital status among male cancer patients

Sex	Marital status at diagnosis-fpc	Selected Events	Site recode ICD-O-3/WHO 2008	Observed	Expected	Persons	Person Years at Risk	SMR(95%CI)	AER(95%CI)
Male	Single (never married)	Septicemia	All Sites	751	319.81	220975	1403392.73	2.35#(2.18, 2.52)	3.07(2.61, 3.53)
Male	Single (never married)	Septicemia	Oral Cavity and Pharynx	24	8.51	8232	45658.55	2.82#(1.81, 4.19)	3.39(0.94, 5.84)
Male	Single (never married)	Septicemia	Stomach	7	2.6	4066	8442.15	2.69#(1.08, 5.54)	5.21(-1.98, 12.4)
Male	Single (never married)	Septicemia	Colon and Rectum	112	37.63	19821	102581.13	2.98#(2.45, 3.58)	7.25(4.91, 9.59)
Male	Single (never married)	Septicemia	Liver	31	1.7	6027	11283.56	18.28#(12.42, 25.95)	25.97(16.05, 35.89)
Male	Single (never married)	Septicemia	Pancreas	13	1.1	5098	4815.13	11.79#(6.28, 20.16)	24.71(9.44, 39.98)
Male	Single (never married)	Septicemia	Lung and Bronchus	68	10.99	25853	38269.34	6.19#(4.8, 7.84)	14.9(10.35, 19.45)
Male	Single (never married)	Septicemia	Skin excluding Basal and Squamous	8	12.28	7942	82984.79	0.65(0.28, 1.28)	-0.52(-1.58, 0.54)
Male	Single (never married)	Septicemia	Prostate	157	160.07	37103	288353.47	0.98(0.83, 1.15)	-0.11(-1.32, 1.1)
Male	Single (never married)	Septicemia	Urinary Bladder	51	22.28	7842	57176.61	2.29#(1.7, 3.01)	5.02(2.09, 7.95)
Male	Single (never married)	Septicemia	Kidney and Renal Pelvis	30	8.33	7194	49388.59	3.60#(2.43, 5.14)	4.39(1.93, 6.85)
Male	Single (never married)	Septicemia	Thyroid	8	2.83	3370	39027.49	2.82#(1.22, 5.56)	1.32(-0.33, 2.97)
Male	Single (never married)	Septicemia	Lymphoma	47	12.8	19187	164176.84	3.67#(2.7, 4.88)	2.08(1.16, 3)
Male	Single (never married)	Septicemia	Leukemia	28	6.39	11998	102343.84	4.38#(2.91, 6.33)	2.11(0.99, 3.23)
Male	Married (including common law)	Septicemia	All Sites	4042	3253.26	1001790	6308848.01	1.24#(1.2, 1.28)	1.25(0.98, 1.52)
Male	Married (including common law)	Septicemia	Oral Cavity and Pharynx	120	65.92	28268	192629.53	1.82#(1.51, 2.18)	2.81(1.42, 4.2)
Male	Married (including common law)	Septicemia	Stomach	87	28.96	24875	62972.24	3.00#(2.41, 3.71)	9.22(5.87, 12.57)
Male	Married (including common law)	Septicemia	Colon and Rectum	547	387.66	105279	706502.48	1.41#(1.3, 1.53)	2.26(1.41, 3.11)
Male	Married (including common law)	Septicemia	Liver	58	7.72	16350	27602.36	7.52#(5.71, 9.72)	18.22(12.47, 23.97)
Male	Married (including common law)	Septicemia	Pancreas	51	10.39	29742	28993.78	4.91#(3.65, 6.45)	14.01(8.72, 19.3)
Male	Married (including common law)	Septicemia	Lung and Bronchus	360	104.26	149823	255725.3	3.45#(3.11, 3.83)	10(8.35, 11.65)
Male	Married (including common law)	Septicemia	Skin excluding Basal and Squamous	103	116.43	35287	338826.76	0.88(0.72, 1.07)	-0.4(-1.26, 0.46)
Male	Married (including common law)	Septicemia	Prostate	1408	1796.42	300330	2799709.66	0.78#(0.74, 0.83)	-1.39(-1.79, -0.99)
Male	Married (including common law)	Septicemia	Urinary Bladder	359	273.47	55111	455939.73	1.31#(1.18, 1.46)	1.88(0.8, 2.96)
Male	Married (including common law)	Septicemia	Kidney and Renal Pelvis	114	76.53	31817	198299.72	1.49#(1.23, 1.79)	1.89(0.53, 3.25)
Male	Married (including common law)	Septicemia	Thyroid	21	24.41	10833	119486.8	0.86(0.53, 1.32)	-0.29(-1.4, 0.82)
Male	Married (including common law)	Septicemia	Lymphoma	197	103.49	46606	318760.53	1.90#(1.65, 2.19)	2.93(1.86, 4)
Male	Married (including common law)	Septicemia	Leukemia	116	57.67	30734	142545.81	2.01#(1.66, 2.41)	4.09(2.28, 5.9)
Male	Unmarried or Domestic Partner	Septicemia	All Sites	4	1.5	2391	6039.91	2.66(0.73, 6.82)	4.14(-3.47, 11.75)
Male	Unmarried or Domestic Partner	Septicemia	Oral Cavity and Pharynx	0	0.08	123	343.32	0(0, 47.74)	-2.25(-18.4, 13.9)
Male	Unmarried or Domestic Partner	Septicemia	Stomach	0	0.03	55	67.56	0(0, 146.69)	-3.72(-53.96, 46.52)
Male	Unmarried or Domestic Partner	Septicemia	Colon and Rectum	0	0.13	222	597.21	0(0, 29.21)	-2.11(-13.94, 9.72)
Male	Unmarried or Domestic Partner	Septicemia	Liver	0	0.03	96	126.35	0(0, 132.02)	-2.21(-29.08, 24.66)
Male	Unmarried or Domestic Partner	Septicemia	Pancreas	0	0.02	90	86.49	0(0, 154.31)	-2.76(-34.8, 29.28)
Male	Unmarried or Domestic Partner	Septicemia	Lung and Bronchus	0	0.12	244	353.68	0(0, 31.31)	-3.33(-22.52, 15.86)
Male	Unmarried or Domestic Partner	Septicemia	Skin excluding Basal and Squamous	0	0.06	97	289.53	0(0, 63.99)	-1.99(-18.57, 14.59)
Male	Unmarried or Domestic Partner	Septicemia	Prostate	0	0.53	491	1577.92	0(0, 6.99)	-3.35(-12.39, 5.69)
Male	Unmarried or Domestic Partner	Septicemia	Urinary Bladder	0	0.1	111	298.68	0(0, 36.99)	-3.34(-24.09, 17.41)
Male	Unmarried or Domestic Partner	Septicemia	Kidney and Renal Pelvis	0	0.07	98	312.23	0(0, 52.06)	-2.27(-18.88, 14.34)
Male	Unmarried or Domestic Partner	Septicemia	Thyroid	0	0.02	38	126.1	0(0, 195.05)	-1.5(-23.48, 20.48)
Male	Unmarried or Domestic Partner	Septicemia	Lymphoma	0	0.08	162	485.6	0(0, 46.89)	-1.62(-13.04, 9.8)
Male	Unmarried or Domestic Partner	Septicemia	Leukemia	1	0.05	78	211.96	20.44(0.52, 113.87)	44.87(-49.67, 139.41)
Male	Unknown	Septicemia	All Sites	455	362.15	109521	713156.69	1.26#(1.14, 1.38)	1.3(0.51, 2.09)
Male	Unknown	Septicemia	Oral Cavity and Pharynx	19	9.54	3633	23972.71	1.99#(1.2, 3.11)	3.95(-0.42, 8.32)
Male	Unknown	Septicemia	Stomach	8	1.39	1318	3352.74	5.74#(2.48, 11.31)	19.71(1.81, 37.61)
Male	Unknown	Septicemia	Colon and Rectum	29	19.36	6794	40141.24	1.50#(1, 2.15)	2.4(-0.99, 5.79)
Male	Unknown	Septicemia	Liver	7	0.59	1451	2209.18	11.81#(4.75, 24.33)	29(4.59, 53.41)
Male	Unknown	Septicemia	Pancreas	4	0.56	1433	1439.71	7.17#(1.95, 18.35)	23.91(-5.13, 52.95)
Male	Unknown	Septicemia	Lung and Bronchus	24	4.75	7528	11413.46	5.05#(3.24, 7.52)	16.87(7.67, 26.07)
Male	Unknown	Septicemia	Skin excluding Basal and Squamous	47	53.02	16590	163832.24	0.89(0.65, 1.18)	-0.37(-1.57, 0.83)
Male	Unknown	Septicemia	Prostate	185	213.61	43449	308894.76	0.87(0.75, 1)	-0.93(-2.2, 0.34)
Male	Unknown	Septicemia	Urinary Bladder	26	16.57	4462	29489.22	1.57#(1.03, 2.3)	3.2(-1.13, 7.53)
Male	Unknown	Septicemia	Kidney and Renal Pelvis	5	3.51	1819	9262.83	1.42(0.46, 3.33)	1.61(-4.56, 7.78)
Male	Unknown	Septicemia	Thyroid	1	1.12	777	6204.38	0.89(0.02, 4.97)	-0.2(-4.8, 4.4)
Male	Unknown	Septicemia	Lymphoma	21	9.67	4330	29854.15	2.17#(1.34, 3.32)	3.8(0.17, 7.43)
Male	Unknown	Septicemia	Leukemia	18	9.66	3701	21830.44	1.86#(1.1, 2.95)	3.82(-0.9, 8.54)
Male	SDW	Septicemia	All Sites	1156	599.31	238329	938783.27	1.93#(1.82, 2.04)	5.93(5.06, 6.8)
Male	SDW	Septicemia	Oral Cavity and Pharynx	45	14.85	9305	37135.65	3.03#(2.21, 4.05)	8.12(4.04, 12.2)
Male	SDW	Septicemia	Stomach	18	6.67	6337	10546.72	2.70#(1.6, 4.27)	10.75(1.53, 19.97)
Male	SDW	Septicemia	Colon and Rectum	167	77.62	25800	112624.82	2.15#(1.84, 2.5)	7.94(5.22, 10.66)
Male	SDW	Septicemia	Liver	24	2.5	5954	7848.57	9.59#(6.14, 14.27)	27.39(14.55, 40.23)
Male	SDW	Septicemia	Pancreas	34	2.62	7576	5094.88	12.99#(8.99, 18.15)	61.6(38.39, 84.81)
Male	SDW	Septicemia	Lung and Bronchus	119	29.81	48721	59933.16	3.99#(3.31, 4.78)	14.88(10.89, 18.87)
Male	SDW	Septicemia	Skin excluding Basal and Squamous	20	16.42	5180	33181.75	1.22(0.74, 1.88)	1.08(-2.48, 4.64)
Male	SDW	Septicemia	Prostate	329	303.66	57086	395950.98	1.08(0.97, 1.21)	0.64(-0.6, 1.88)
Male	SDW	Septicemia	Urinary Bladder	95	54.13	13750	71675.29	1.75#(1.42, 2.15)	5.7(2.36, 9.04)
Male	SDW	Septicemia	Kidney and Renal Pelvis	36	12.97	6581	28725.71	2.77#(1.94, 3.84)	8.02(3.25, 12.79)
Male	SDW	Septicemia	Thyroid	1	2.72	1177	10028.62	0.37(0.01, 2.05)	-1.71(-5.48, 2.06)
Male	SDW	Septicemia	Lymphoma	53	17.86	9171	41073.45	2.97#(2.22, 3.88)	8.56(4.55, 12.57)
Male	SDW	Septicemia	Leukemia	34	12.16	6530	21134.68	2.80#(1.94, 3.91)	10.33(4.03, 16.63)

Table S19

Septicemia SMRs and AERs by marital status among female cancer patients

Sex	Marital status at diagnosis-fpc	Selected Events	Site recode ICD-O-3/WHO 2008	Observed	Expected	Persons	Person Years at Risk	SMR(95%CI)	AER(95%CI)
Female	Single (never married)	Septicemia	All Sites	673	314.38	198748	1555418.91	2.14#(1.98, 2.31)	2.31(1.91, 2.71)
Female	Single (never married)	Septicemia	Oral Cavity and Pharynx	14	4.24	3008	23768.68	3.30#(1.81, 5.54)	4.11(0.59, 7.63)
Female	Single (never married)	Septicemia	Stomach	12	2.35	2369	6936.75	5.11#(2.64, 8.92)	13.91(3.21, 24.61)
Female	Single (never married)	Septicemia	Colon and Rectum	82	45.44	16798	97097.02	1.80#(1.44, 2.24)	3.77(1.49, 6.05)
Female	Single (never married)	Septicemia	Liver	3	0.55	1647	5298.42	5.41#(1.12, 15.81)	4.62(-2.35, 11.59)
Female	Single (never married)	Septicemia	Pancreas	10	1.35	4220	4995.33	7.38#(3.54, 13.58)	17.31(4.1, 30.52)
Female	Single (never married)	Septicemia	Lung and Bronchus	38	10.26	15816	33813.75	3.70#(2.62, 5.08)	8.2(4.18, 12.22)
Female	Single (never married)	Septicemia	Skin excluding Basal and Squamous	18	11.15	7546	96511.81	1.61(0.96, 2.55)	0.71(-0.39, 1.81)
Female	Single (never married)	Septicemia	Breast	159	127.22	52233	458241.54	1.25#(1.06, 1.46)	0.69(-0.03, 1.41)
Female	Single (never married)	Septicemia	Cervix Uteri	20	5.89	6390	65815.33	3.39#(2.07, 5.24)	2.14(0.62, 3.66)
Female	Single (never married)	Septicemia	Corpus Uteri	80	37.59	14680	129755.32	2.13#(1.69, 2.65)	3.27(1.63, 4.91)
Female	Single (never married)	Septicemia	Ovary	20	7.89	8152	62030.68	2.54#(1.55, 3.92)	1.95(0.28, 3.62)
Female	Single (never married)	Septicemia	Urinary Bladder	29	10.13	3041	22164.68	2.86#(1.92, 4.11)	8.52(2.99, 14.05)
Female	Single (never married)	Septicemia	Kidney and Renal Pelvis	18	5.75	4289	37263.22	3.13#(1.85, 4.94)	3.29(0.73, 5.85)
Female	Single (never married)	Septicemia	Thyroid	8	8.6	11341	137907.91	0.93(0.4, 1.83)	-0.04(-0.62, 0.54)
Female	Single (never married)	Septicemia	Lymphoma	39	11.44	10967	110062.52	3.41#(2.42, 4.66)	2.5(1.24, 3.76)
Female	Single (never married)	Septicemia	Leukemia	20	4.91	8627	79903.14	4.08#(2.49, 6.3)	1.89(0.67, 3.11)
Female	Married (including common law)	Septicemia	All Sites	2258	1898.06	712264	6161689.58	1.19#(1.14, 1.24)	0.58(0.37, 0.79)
Female	Married (including common law)	Septicemia	Oral Cavity and Pharynx	41	22.72	9662	81899.17	1.80#(1.3, 2.45)	2.23(0.32, 4.14)
Female	Married (including common law)	Septicemia	Stomach	29	11.71	9218	32509.32	2.48#(1.66, 3.56)	5.32(1.47, 9.17)
Female	Married (including common law)	Septicemia	Colon and Rectum	326	257.67	66724	548057.78	1.27#(1.13, 1.41)	1.25(0.39, 2.11)
Female	Married (including common law)	Septicemia	Liver	21	1.95	4207	8256.76	10.78#(6.67, 16.47)	23.07(11.71, 34.43)
Female	Married (including common law)	Septicemia	Pancreas	47	5.65	17845	21488.22	8.31#(6.11, 11.05)	19.24(12.63, 25.85)
Female	Married (including common law)	Septicemia	Lung and Bronchus	157	54.04	68482	171952.61	2.91#(2.47, 3.4)	5.99(4.33, 7.65)
Female	Married (including common law)	Septicemia	Skin excluding Basal and Squamous	51	71.79	25788	332709.18	0.71#(0.53, 0.93)	-0.62(-1.27, 0.03)
Female	Married (including common law)	Septicemia	Breast	688	826.8	241526	2652151.04	0.83#(0.77, 0.9)	-0.52(-0.81, -0.23)
Female	Married (including common law)	Septicemia	Cervix Uteri	45	29.86	15371	208036.88	1.51#(1.1, 2.02)	0.73(-0.09, 1.55)
Female	Married (including common law)	Septicemia	Corpus Uteri	271	244.77	52449	630046.5	1.11(0.98, 1.25)	0.42(-0.29, 1.13)
Female	Married (including common law)	Septicemia	Ovary	59	41.48	26644	177223.13	1.42#(1.08, 1.83)	0.99(-0.12, 2.1)
Female	Married (including common law)	Septicemia	Urinary Bladder	71	55.15	11860	117336.23	1.29#(1.01, 1.62)	1.35(-0.53, 3.23)
Female	Married (including common law)	Septicemia	Kidney and Renal Pelvis	46	32.86	13876	102873.98	1.40#(1.02, 1.87)	1.28(-0.41, 2.97)
Female	Married (including common law)	Septicemia	Thyroid	34	51.65	31662	400397.67	0.66#(0.46, 0.92)	-0.44(-0.89, 0.01)
Female	Married (including common law)	Septicemia	Lymphoma	87	70.39	30244	256593.43	1.24(0.99, 1.52)	0.65(-0.31, 1.61)
Female	Married (including common law)	Septicemia	Leukemia	50	26.17	15002	81999.32	1.91#(1.42, 2.52)	2.91(0.82, 5)
Female	Unmarried or Domestic Partner	Septicemia	All Sites	2	0.92	2332	6517.78	2.18(0.26, 7.88)	1.66(-3.48, 6.8)
Female	Unmarried or Domestic Partner	Septicemia	Oral Cavity and Pharynx	0	0.03	34	107.48	0(0, 144.23)	-2.38(-33.96, 29.2)
Female	Unmarried or Domestic Partner	Septicemia	Stomach	0	0.01	19	33.57	0(0, 314.63)	-3.49(-61.87, 54.89)
Female	Unmarried or Domestic Partner	Septicemia	Colon and Rectum	0	0.07	164	454.76	0(0, 55.09)	-1.47(-12.87, 9.93)
Female	Unmarried or Domestic Partner	Septicemia	Liver	0	0.01	33	41.64	0(0, 414.57)	-2.14(-49.2, 44.92)
Female	Unmarried or Domestic Partner	Septicemia	Pancreas	0	0.01	51	62.39	0(0, 380.03)	-1.56(-32.97, 29.85)
Female	Unmarried or Domestic Partner	Septicemia	Lung and Bronchus	1	0.07	211	314.3	14.91(0.38, 83.07)	29.68(-34.73, 94.09)
Female	Unmarried or Domestic Partner	Septicemia	Skin excluding Basal and Squamous	0	0.02	94	311.09	0(0, 154.7)	-0.77(-9.68, 8.14)
Female	Unmarried or Domestic Partner	Septicemia	Breast	0	0.35	790	2596.68	0(0, 10.66)	-1.33(-5.8, 3.14)
Female	Unmarried or Domestic Partner	Septicemia	Cervix Uteri	1	0.01	86	202.24	94.88#(2.4, 528.63)	48.93(-48.23, 146.09)
Female	Unmarried or Domestic Partner	Septicemia	Corpus Uteri	0	0.09	189	588.23	0(0, 40.69)	-1.54(-11.54, 8.46)
Female	Unmarried or Domestic Partner	Septicemia	Ovary	0	0.03	85	233.44	0(0, 141.46)	-1.12(-15.66, 13.42)
Female	Unmarried or Domestic Partner	Septicemia	Urinary Bladder	0	0.02	21	56.95	0(0, 163.91)	-3.95(-52.61, 44.71)
Female	Unmarried or Domestic Partner	Septicemia	Kidney and Renal Pelvis	0	0.02	48	137.23	0(0, 163.08)	-1.65(-21.85, 18.55)
Female	Unmarried or Domestic Partner	Septicemia	Thyroid	0	0.03	135	417.24	0(0, 107.58)	-0.82(-8.96, 7.32)
Female	Unmarried or Domestic Partner	Septicemia	Lymphoma	0	0.02	71	197.42	0(0, 155.22)	-1.2(-15.24, 12.84)
Female	Unmarried or Domestic Partner	Septicemia	Leukemia	0	0.03	58	221.55	0(0, 106.98)	-1.56(-16.88, 13.76)
Female	Unknown	Septicemia	All Sites	304	199.18	80309	569137.7	1.53#(1.36, 1.71)	1.84(1.07, 2.61)
Female	Unknown	Septicemia	Oral Cavity and Pharynx	5	4.86	1785	12086.98	1.03(0.33, 2.4)	0.12(-4.97, 5.21)
Female	Unknown	Septicemia	Stomach	2	1.6	955	2960.09	1.25(0.15, 4.53)	1.37(-11.19, 13.93)
Female	Unknown	Septicemia	Colon and Rectum	46	24.47	6877	42858.95	1.88#(1.38, 2.51)	5.02(1.18, 8.86)
Female	Unknown	Septicemia	Liver	1	0.27	507	932.92	3.66(0.09, 20.41)	7.79(-15.88, 31.46)
Female	Unknown	Septicemia	Pancreas	6	0.59	1466	1455.03	10.21#(3.75, 22.22)	37.2(2.69, 71.71)
Female	Unknown	Septicemia	Lung and Bronchus	13	5.06	6076	11773.7	2.57#(1.37, 4.39)	6.74(-0.33, 13.81)
Female	Unknown	Septicemia	Skin excluding Basal and Squamous	30	40.31	15757	172152.75	0.74(0.5, 1.06)	-0.6(-1.55, 0.35)
Female	Unknown	Septicemia	Breast	64	53.74	17353	132100.93	1.19(0.92, 1.52)	0.78(-0.83, 2.39)
Female	Unknown	Septicemia	Cervix Uteri	10	3.06	1884	20163.3	3.27#(1.57, 6.01)	3.44(-0.07, 6.95)
Female	Unknown	Septicemia	Corpus Uteri	22	13.01	4070	33403.12	1.69#(1.06, 2.56)	2.69(-0.78, 6.16)
Female	Unknown	Septicemia	Ovary	9	2.46	1757	9169.57	3.66#(1.67, 6.95)	7.14(-0.09, 14.37)
Female	Unknown	Septicemia	Urinary Bladder	6	6.52	1734	11634.02	0.92(0.34, 2)	-0.44(-6.4, 5.52)
Female	Unknown	Septicemia	Kidney and Renal Pelvis	6	2.43	1201	6806.36	2.47(0.91, 5.37)	5.24(-3.12, 13.6)
Female	Unknown	Septicemia	Thyroid	4	3.05	2529	22495.22	1.31(0.36, 3.35)	0.42(-1.89, 2.73)
Female	Unknown	Septicemia	Lymphoma	20	10.26	3835	26457.3	1.95#(1.19, 3.01)	3.68(-0.39, 7.75)
Female	Unknown	Septicemia	Leukemia	16	9.29	2983	18375.51	1.72(0.98, 2.8)	3.65(-1.71, 9.01)
Female	SDW	Septicemia	All Sites	2281	1572.32	501038	2565722.14	1.45#(1.39, 1.51)	2.76(2.29, 3.23)
Female	SDW	Septicemia	Oral Cavity and Pharynx	40	20.9	7869	38244.6	1.91#(1.37, 2.61)	4.99(0.99, 8.99)
Female	SDW	Septicemia	Stomach	45	16.57	10192	22774.26	2.72#(1.98, 3.63)	12.49(5.74, 19.24)
Female	SDW	Septicemia	Colon and Rectum	400	300.48	68392	355794.91	1.33#(1.2, 1.47)	2.8(1.34, 4.26)
Female	SDW	Septicemia	Liver	20	2.26	4321	5195.07	8.86#(5.41, 13.68)	34.15(16.38, 51.92)
Female	SDW	Septicemia	Pancreas	38	8.79	19728	14581.12	4.32#(3.06, 5.93)	20.03(10.85, 29.21)
Female	SDW	Septicemia	Lung and Bronchus	168	66.71	74494	127938.76	2.52#(2.15, 2.93)	7.92(5.57, 10.27)
Female	SDW	Septicemia	Skin excluding Basal and Squamous	35	39.72	9017	75986.16	0.88(0.61, 1.23)	-0.62(-2.85, 1.61)
Female	SDW	Septicemia	Breast	658	617.09	123278	1025389.35	1.07(0.99, 1.15)	0.4(-0.28, 1.08)
Female	SDW	Septicemia	Cervix Uteri	67	23.99	9415	86669.09	2.79#(2.16, 3.55)	4.96(2.8, 7.12)
Female	SDW	Septicemia	Corpus Uteri	168	140.89	27756	239070.88	1.19#(1.02, 1.39)	1.13(-0.31, 2.57)
Female	SDW	Septicemia	Ovary	52	27.62	16581	60189.6	1.88#(1.41, 2.47)	4.05(1.15, 6.95)
Female	SDW	Septicemia	Urinary Bladder	92	61.53	12894	74531.85	1.50#(1.21, 1.83)	4.09(0.83, 7.35)
Female	SDW	Septicemia	Kidney and Renal Pelvis	53	29.16	9717	49744.74	1.82#(1.36, 2.38)	4.79(1.22, 8.36)
Female	SDW	Septicemia	Thyroid	13	20.66	6986	71949.72	0.63(0.33, 1.08)	-1.07(-2.65, 0.51)
Female	SDW	Septicemia	Lymphoma	93	60.58	20763	100905.45	1.54#(1.24, 1.88)	3.21(0.8, 5.62)
Female	SDW	Septicemia	Leukemia	50	29.97	13097	43450.39	1.67#(1.24, 2.2)	4.61(0.58, 8.64)

Table S20

Septicemia SMRs and AERs by tumor grade among male cancer patients

Sex	Grade (thru 2017)-fpc	Selected Events	Site recode ICD-O-3/WHO 2008	Observed	Expected	Persons	Person Years at Risk	SMR(95%CI)	AER(95%CI)
Male	Well differentiated; Grade I	Septicemia	All Sites	707	567.12	114196	966960.87	1.25#(1.16, 1.34)	1.45(0.73, 2.17)
Male	Well differentiated; Grade I	Septicemia	Oral Cavity and Pharynx	44	22.14	6405	54130.09	1.99#(1.44, 2.67)	4.04(1.1, 6.98)
Male	Well differentiated; Grade I	Septicemia	Stomach	13	3.67	1784	7502.26	3.55#(1.89, 6.06)	12.44(1.78, 23.1)
Male	Well differentiated; Grade I	Septicemia	Colon and Rectum	121	70.13	16113	126474.24	1.73#(1.43, 2.06)	4.02(1.88, 6.16)
Male	Well differentiated; Grade I	Septicemia	Liver	14	1.93	2490	6980.06	7.25#(3.96, 12.16)	17.29(6.09, 28.49)
Male	Well differentiated; Grade I	Septicemia	Pancreas	11	1.67	2381	5366.92	6.59#(3.29, 11.79)	17.38(4.39, 30.37)
Male	Well differentiated; Grade I	Septicemia	Lung and Bronchus	29	10.45	6518	23534.19	2.78#(1.86, 3.99)	7.88(2.65, 13.11)
Male	Well differentiated; Grade I	Septicemia	Skin excluding Basal and Squamous	4	0.83	246	2551.77	4.81#(1.31, 12.3)	12.41(-4.46, 29.28)
Male	Well differentiated; Grade I	Septicemia	Prostate	320	339.96	50291	458560.5	0.94(0.84, 1.05)	-0.44(-1.54, 0.66)
Male	Well differentiated; Grade I	Septicemia	Urinary Bladder	82	66.05	10384	122693.16	1.24(0.99, 1.54)	1.3(-0.64, 3.24)
Male	Well differentiated; Grade I	Septicemia	Kidney and Renal Pelvis	19	10.66	3022	29559.68	1.78#(1.07, 2.78)	2.82(-0.79, 6.43)
Male	Well differentiated; Grade I	Septicemia	Thyroid	4	4.07	1972	24776.26	0.98(0.27, 2.52)	-0.03(-2.28, 2.22)
Male	Well differentiated; Grade I	Septicemia	Lymphoma	9	3.51	1323	9810.59	2.57#(1.17, 4.87)	5.6(-1.46, 12.66)
Male	Well differentiated; Grade I	Septicemia	Leukemia	0	0.16	58	409.47	0(0, 23.18)	-3.89(-23.03, 15.25)
Male	Moderately differentiated; Grade II	Septicemia	All Sites	1966	1892.69	381645	3220170.21	1.04(0.99, 1.09)	0.23(-0.15, 0.61)
Male	Moderately differentiated; Grade II	Septicemia	Oral Cavity and Pharynx	48	26.43	14046	82344.22	1.82#(1.34, 2.41)	2.62(0.57, 4.67)
Male	Moderately differentiated; Grade II	Septicemia	Stomach	29	11.48	7479	21479.66	2.53#(1.69, 3.63)	8.15(2.35, 13.95)
Male	Moderately differentiated; Grade II	Septicemia	Colon and Rectum	451	303.46	79380	538227.66	1.49#(1.35, 1.63)	2.74(1.74, 3.74)
Male	Moderately differentiated; Grade II	Septicemia	Liver	14	2.52	3357	9252.99	5.56#(3.04, 9.32)	12.41(3.81, 21.01)
Male	Moderately differentiated; Grade II	Septicemia	Pancreas	15	2.47	5268	7515.6	6.06#(3.39, 10)	16.67(5.78, 27.56)
Male	Moderately differentiated; Grade II	Septicemia	Lung and Bronchus	94	29.5	24766	67266.59	3.19#(2.58, 3.9)	9.59(6.35, 12.83)
Male	Moderately differentiated; Grade II	Septicemia	Skin excluding Basal and Squamous	0	1.38	380	4244.23	0(0, 2.68)	-3.24(-8.66, 2.18)
Male	Moderately differentiated; Grade II	Septicemia	Prostate	968	1294.18	185037	2009423.93	0.75#(0.7, 0.8)	-1.62(-2.08, -1.16)
Male	Moderately differentiated; Grade II	Septicemia	Urinary Bladder	172	137.69	23578	233053.46	1.25#(1.07, 1.45)	1.47(-0.01, 2.95)
Male	Moderately differentiated; Grade II	Septicemia	Kidney and Renal Pelvis	41	29.88	10789	87435.46	1.37(0.98, 1.86)	1.27(-0.62, 3.16)
Male	Moderately differentiated; Grade II	Septicemia	Thyroid	0	1.28	501	6501.23	0(0, 2.88)	-1.97(-5.38, 1.44)
Male	Moderately differentiated; Grade II	Septicemia	Lymphoma	4	2.43	923	7238.48	1.65(0.45, 4.22)	2.17(-4.69, 9.03)
Male	Moderately differentiated; Grade II	Septicemia	Leukemia	0	0.02	13	93.84	0(0, 162.65)	-2.42(-31.95, 27.11)
Male	Poorly differentiated; Grade III	Septicemia	All Sites	1156	896.8	301408	1645995.22	1.29#(1.22, 1.37)	1.57(1.03, 2.11)
Male	Poorly differentiated; Grade III	Septicemia	Oral Cavity and Pharynx	50	18.41	11310	65193.62	2.72#(2.02, 3.58)	4.84(2.35, 7.33)
Male	Poorly differentiated; Grade III	Septicemia	Stomach	44	14.52	16082	33143.09	3.03#(2.2, 4.07)	8.89(4.37, 13.41)
Male	Poorly differentiated; Grade III	Septicemia	Colon and Rectum	94	55.75	21908	107753.43	1.69#(1.36, 2.06)	3.55(1.32, 5.78)
Male	Poorly differentiated; Grade III	Septicemia	Liver	9	0.71	2211	2834.73	12.61#(5.77, 23.94)	29.23(7.72, 50.74)
Male	Poorly differentiated; Grade III	Septicemia	Pancreas	11	1.72	6353	4991.18	6.38#(3.19, 11.42)	18.59(4.6, 32.58)
Male	Poorly differentiated; Grade III	Septicemia	Lung and Bronchus	136	40.81	56734	101882.09	3.33#(2.8, 3.94)	9.34(6.78, 11.9)
Male	Poorly differentiated; Grade III	Septicemia	Skin excluding Basal and Squamous	3	2.15	602	3846.78	1.4(0.29, 4.09)	2.22(-9.34, 13.78)
Male	Poorly differentiated; Grade III	Septicemia	Prostate	544	639.15	131535	1058386.38	0.85#(0.78, 0.93)	-0.9(-1.54, -0.26)
Male	Poorly differentiated; Grade III	Septicemia	Urinary Bladder	122	75.34	17263	116181.54	1.62#(1.34, 1.93)	4.02(1.65, 6.39)
Male	Poorly differentiated; Grade III	Septicemia	Kidney and Renal Pelvis	32	15.02	7449	42315.83	2.13#(1.46, 3.01)	4.01(0.83, 7.19)
Male	Poorly differentiated; Grade III	Septicemia	Thyroid	1	0.67	325	2327.68	1.5(0.04, 8.34)	1.43(-9.45, 12.31)
Male	Poorly differentiated; Grade III	Septicemia	Lymphoma	11	5.8	3055	21554.01	1.9(0.95, 3.4)	2.41(-1.32, 6.14)
Male	Poorly differentiated; Grade III	Septicemia	Leukemia	0	0.12	50	266.9	0(0, 29.84)	-4.63(-30.06, 20.8)
Male	Undifferentiated; anaplastic; Grade IV	Septicemia	All Sites	205	89.64	69654	221559.43	2.29#(1.98, 2.62)	5.21(3.69, 6.73)
Male	Undifferentiated; anaplastic; Grade IV	Septicemia	Oral Cavity and Pharynx	4	2.62	1834	12284.35	1.53(0.42, 3.91)	1.12(-2.98, 5.22)
Male	Undifferentiated; anaplastic; Grade IV	Septicemia	Stomach	1	1.05	1294	2630.61	0.95(0.02, 5.3)	-0.2(-10.87, 10.47)
Male	Undifferentiated; anaplastic; Grade IV	Septicemia	Colon and Rectum	5	3.66	2039	7459.65	1.36(0.44, 3.18)	1.79(-5.94, 9.52)
Male	Undifferentiated; anaplastic; Grade IV	Septicemia	Liver	3	0.11	340	479.73	27.60#(5.69, 80.67)	60.27(-11.56, 132.1)
Male	Undifferentiated; anaplastic; Grade IV	Septicemia	Pancreas	1	0.12	817	521.16	8.12(0.21, 45.25)	16.83(-22.94, 56.6)
Male	Undifferentiated; anaplastic; Grade IV	Septicemia	Lung and Bronchus	52	10.38	26424	33997.47	5.01#(3.74, 6.57)	12.24(7.69, 16.79)
Male	Undifferentiated; anaplastic; Grade IV	Septicemia	Skin excluding Basal and Squamous	0	0.82	268	1601.75	0(0, 4.52)	-5.1(-16.18, 5.98)
Male	Undifferentiated; anaplastic; Grade IV	Septicemia	Prostate	10	10.16	2677	14257.49	0.98(0.47, 1.81)	-0.11(-6.28, 6.06)
Male	Undifferentiated; anaplastic; Grade IV	Septicemia	Urinary Bladder	87	46.27	15021	72171.39	1.88#(1.51, 2.32)	5.64(2.51, 8.77)
Male	Undifferentiated; anaplastic; Grade IV	Septicemia	Kidney and Renal Pelvis	7	3.28	2628	9398.21	2.14(0.86, 4.4)	3.96(-2.72, 10.64)
Male	Undifferentiated; anaplastic; Grade IV	Septicemia	Thyroid	0	0.23	405	691.26	0(0, 16.06)	-3.32(-16.92, 10.28)
Male	Undifferentiated; anaplastic; Grade IV	Septicemia	Lymphoma	5	1	1121	7113.86	5.02#(1.63, 11.72)	5.63(-1.12, 12.38)
Male	Undifferentiated; anaplastic; Grade IV	Septicemia	Leukemia	0	0.03	98	281.57	0(0, 114.69)	-1.14(-13.2, 10.92)
Male	Unknown	Septicemia	All Sites	2001	936.51	544750	2863569.29	2.14#(2.04, 2.23)	3.72(3.35, 4.09)
Male	Unknown	Septicemia	Oral Cavity and Pharynx	57	28.37	12167	82403.12	2.01#(1.52, 2.6)	3.47(1.27, 5.67)
Male	Unknown	Septicemia	Stomach	30	8.52	8290	19449.18	3.52#(2.38, 5.03)	11.04(4.79, 17.29)
Male	Unknown	Septicemia	Colon and Rectum	171	87.23	30163	175553.89	1.96#(1.68, 2.28)	4.77(2.98, 6.56)
Male	Unknown	Septicemia	Liver	74	6.78	18960	27949.66	10.91#(8.57, 13.7)	24.05(17.75, 30.35)
Male	Unknown	Septicemia	Pancreas	56	8.06	25928	20266.02	6.95#(5.25, 9.03)	23.66(15.93, 31.39)
Male	Unknown	Septicemia	Lung and Bronchus	237	56.36	108098	133090.59	4.21#(3.69, 4.78)	13.57(11.05, 16.09)
Male	Unknown	Septicemia	Skin excluding Basal and Squamous	168	191.17	57805	601720.12	0.88(0.75, 1.02)	-0.39(-1.01, 0.23)
Male	Unknown	Septicemia	Prostate	226	180.46	38308	225096.97	1.25#(1.09, 1.43)	2.02(0.27, 3.77)
Male	Unknown	Septicemia	Urinary Bladder	63	38.63	9548	65618.22	1.63#(1.25, 2.09)	3.71(0.7, 6.72)
Male	Unknown	Septicemia	Kidney and Renal Pelvis	83	41.48	19191	113347.24	2.00#(1.59, 2.48)	3.66(1.73, 5.59)
Male	Unknown	Septicemia	Thyroid	26	24.63	11335	139058.92	1.06(0.69, 1.55)	0.1(-0.9, 1.1)
Male	Unknown	Septicemia	Lymphoma	126	49.8	28601	262993.61	2.53#(2.11, 3.01)	2.9(1.91, 3.89)
Male	Unknown	Septicemia	Leukemia	129	52.86	35352	184587.32	2.44#(2.04, 2.9)	4.12(2.69, 5.55)

Table S21

Septicemia SMRs and AERs by tumor grade among female cancer patients

Sex	Grade (thr	Selected Events	Site recode ICD-O-3/WHO 2008	Observed	Expected	Persons	Person Years at Risk	SMR(95%CI)	AER(95%CI)
Female	Well differe	Septicemia	All Sites	671	639.17	150489	1601358.83	1.05(0.97, 1.13)	0.2(-0.24, 0.64)
Female	Well differe	Septicemia	Oral Cavity and Pharynx	22	12.4	3562	29907.27	1.77#(1.11, 2.69)	3.21(-0.63, 7.05)
Female	Well differe	Septicemia	Stomach	4	3.24	1172	6244.23	1.23(0.34, 3.16)	1.22(-7.22, 9.66)
Female	Well differe	Septicemia	Colon and Rectum	97	82.39	15807	135899.32	1.18(0.95, 1.44)	1.08(-0.85, 3.01)
Female	Well differe	Septicemia	Liver	4	0.59	797	2289.02	6.73#(1.83, 17.23)	14.88(-3.45, 33.21)
Female	Well differe	Septicemia	Pancreas	7	1.66	2277	5807.2	4.22#(1.7, 8.7)	9.2(-0.73, 19.13)
Female	Well differe	Septicemia	Lung and Bronchus	28	15.7	6364	34233.55	1.78#(1.19, 2.58)	3.59(-0.19, 7.37)
Female	Well differe	Septicemia	Skin excluding Basal and Squamous	0	0.72	205	2489.96	0(0, 5.13)	-2.89(-9.57, 3.79)
Female	Well differe	Septicemia	Breast	189	250.79	59677	605562.2	0.75#(0.65, 0.87)	-1.02(-1.7, -0.34)
Female	Well differe	Septicemia	Cervix Uteri	16	5.4	2673	35208.52	2.96#(1.69, 4.81)	3.01(0.44, 5.58)
Female	Well differe	Septicemia	Corpus Uteri	192	179.91	31607	446645.08	1.07(0.92, 1.23)	0.27(-0.58, 1.12)
Female	Well differe	Septicemia	Ovary	8	10.39	3305	45353.01	0.77(0.33, 1.52)	-0.53(-2.38, 1.32)
Female	Well differe	Septicemia	Urinary Bladder	41	28.27	4047	52617.9	1.45#(1.04, 1.97)	2.42(-0.68, 5.52)
Female	Well differe	Septicemia	Kidney and Renal Pelvis	15	9.45	2536	26543.24	1.59(0.89, 2.62)	2.09(-1.56, 5.74)
Female	Well differe	Septicemia	Thyroid	9	11.51	6805	92113.89	0.78(0.36, 1.48)	-0.27(-1.23, 0.69)
Female	Well differe	Septicemia	Lymphoma	2	4.63	1237	10779.85	0.43(0.05, 1.56)	-2.44(-7.12, 2.24)
Female	Well differe	Septicemia	Leukemia	0	0.11	43	324.98	0(0, 32.82)	-3.46(-23.46, 16.54)
Female	Moderately	Septicemia	All Sites	1409	1159.81	307134	2679956.57	1.21#(1.15, 1.28)	0.93(0.56, 1.3)
Female	Moderately	Septicemia	Oral Cavity and Pharynx	31	15.28	6803	46636.56	2.03#(1.38, 2.88)	3.37(0.51, 6.23)
Female	Moderately	Septicemia	Stomach	16	7.49	3514	12096.15	2.14#(1.22, 3.47)	7.04(-0.81, 14.89)
Female	Moderately	Septicemia	Colon and Rectum	458	348.61	76894	570166.11	1.31#(1.2, 1.44)	1.92(0.94, 2.9)
Female	Moderately	Septicemia	Liver	7	0.92	1128	3166.99	7.59#(3.05, 15.64)	19.19(1.79, 36.59)
Female	Moderately	Septicemia	Pancreas	14	2.67	5202	8031.74	5.24#(2.87, 8.8)	14.11(4.15, 24.07)
Female	Moderately	Septicemia	Lung and Bronchus	53	27.21	16987	65960.01	1.95#(1.46, 2.55)	3.91(1.25, 6.57)
Female	Moderately	Septicemia	Skin excluding Basal and Squamous	3	1.32	305	3736.22	2.26(0.47, 6.62)	4.48(-6.42, 15.38)
Female	Moderately	Septicemia	Breast	439	484.21	124425	1259743.5	0.91#(0.82, 1)	-0.36(-0.83, 0.11)
Female	Moderately	Septicemia	Cervix Uteri	22	13.73	7966	83823.72	1.60#(1, 2.43)	0.99(-0.41, 2.39)
Female	Moderately	Septicemia	Corpus Uteri	168	134.3	23138	294748.8	1.25#(1.07, 1.46)	1.14(-0.02, 2.3)
Female	Moderately	Septicemia	Ovary	21	13.56	6424	55197.36	1.55(0.96, 2.37)	1.35(-0.74, 3.44)
Female	Moderately	Septicemia	Urinary Bladder	55	51.71	8352	87255.37	1.06(0.8, 1.38)	0.38(-1.94, 2.7)
Female	Moderately	Septicemia	Kidney and Renal Pelvis	26	20.03	6584	56559.39	1.3(0.85, 1.9)	1.06(-1.29, 3.41)
Female	Moderately	Septicemia	Thyroid	4	3.62	1532	23021.89	1.11(0.3, 2.83)	0.17(-2.18, 2.52)
Female	Moderately	Septicemia	Lymphoma	5	3.27	958	8330.61	1.53(0.5, 3.57)	2.07(-4.69, 8.83)
Female	Moderately	Septicemia	Leukemia	0	0.06	18	148.33	0(0, 64.25)	-3.87(-36.23, 28.49)
Female	Poorly diffe	Septicemia	All Sites	922	607.54	258789	1703061.58	1.52#(1.42, 1.62)	1.85(1.4, 2.3)
Female	Poorly diffe	Septicemia	Oral Cavity and Pharynx	24	6.85	3907	22643.78	3.51#(2.25, 5.22)	7.58(2.77, 12.39)
Female	Poorly diffe	Septicemia	Stomach	41	11.78	9844	24899.3	3.48#(2.5, 4.72)	11.73(6.02, 17.44)
Female	Poorly diffe	Septicemia	Colon and Rectum	124	88.14	24915	134448.26	1.41#(1.17, 1.68)	2.67(0.55, 4.79)
Female	Poorly diffe	Septicemia	Liver	5	0.4	1015	1440.22	12.62#(4.1, 29.45)	31.97(0.4, 63.54)
Female	Poorly diffe	Septicemia	Pancreas	13	1.51	5467	4637.87	8.59#(4.58, 14.7)	24.77(8.69, 40.85)
Female	Poorly diffe	Septicemia	Lung and Bronchus	91	27.38	34758	76972.07	3.32#(2.68, 4.08)	8.27(5.5, 11.04)
Female	Poorly diffe	Septicemia	Skin excluding Basal and Squamous	1	1.37	377	2931.39	0.73(0.02, 4.05)	-1.28(-11.57, 9.01)
Female	Poorly diffe	Septicemia	Breast	326	315.03	107585	1018026.36	1.03(0.93, 1.15)	0.11(-0.38, 0.6)
Female	Poorly diffe	Septicemia	Cervix Uteri	44	13.06	7848	70921.79	3.37#(2.45, 4.52)	4.36(2.27, 6.45)
Female	Poorly diffe	Septicemia	Corpus Uteri	75	55.28	14659	115444.42	1.36#(1.07, 1.7)	1.71(-0.23, 3.65)
Female	Poorly diffe	Septicemia	Ovary	40	20.79	15112	72710.09	1.92#(1.37, 2.62)	2.64(0.54, 4.74)
Female	Poorly diffe	Septicemia	Urinary Bladder	50	25.17	6527	38166.97	1.99#(1.47, 2.62)	6.51(2.06, 10.96)
Female	Poorly diffe	Septicemia	Kidney and Renal Pelvis	14	8.29	3842	22730.39	1.69(0.92, 2.83)	2.51(-1.56, 6.58)
Female	Poorly diffe	Septicemia	Thyroid	1	0.92	525	4455.51	1.09(0.03, 6.09)	0.19(-5.9, 6.28)
Female	Poorly diffe	Septicemia	Lymphoma	10	7.71	2893	22968.2	1.3(0.62, 2.38)	1(-2.59, 4.59)
Female	Poorly diffe	Septicemia	Leukemia	0	0.04	34	183.72	0(0, 92.08)	-2.18(-23.51, 19.15)
Female	Undifferen	Septicemia	All Sites	179	88.31	59202	257796.85	2.03#(1.74, 2.35)	3.52(2.28, 4.76)
Female	Undifferen	Septicemia	Oral Cavity and Pharynx	1	1.3	819	6166.8	0.77(0.02, 4.28)	-0.49(-5.31, 4.33)
Female	Undifferen	Septicemia	Stomach	3	0.67	779	1775.49	4.47(0.92, 13.06)	13.12(-8.01, 34.25)
Female	Undifferen	Septicemia	Colon and Rectum	12	6.08	2472	10188.04	1.97#(1.02, 3.45)	5.81(-2.37, 13.99)
Female	Undifferen	Septicemia	Liver	1	0.05	145	439.61	19.19(0.49, 106.89)	21.56(-24.08, 67.2)
Female	Undifferen	Septicemia	Pancreas	0	0.11	581	461.15	0(0, 33.68)	-2.37(-16.46, 11.72)
Female	Undifferen	Septicemia	Lung and Bronchus	28	6.94	16628	26375.36	4.04#(2.68, 5.83)	7.99(3.6, 12.38)
Female	Undifferen	Septicemia	Skin excluding Basal and Squamous	3	0.58	193	1316.2	5.16#(1.06, 15.07)	18.37(-9.78, 46.52)
Female	Undifferen	Septicemia	Breast	29	28.33	6899	81745.14	1.02(0.69, 1.47)	0.08(-1.74, 1.9)
Female	Undifferen	Septicemia	Cervix Uteri	2	1.22	817	5822.34	1.65(0.2, 5.94)	1.35(-4.69, 7.39)
Female	Undifferen	Septicemia	Corpus Uteri	28	9.83	4403	22887.63	2.85#(1.89, 4.12)	7.94(2.68, 13.2)
Female	Undifferen	Septicemia	Ovary	16	6.73	5550	24988.45	2.38#(1.36, 3.86)	3.71(-0.03, 7.45)
Female	Undifferen	Septicemia	Urinary Bladder	25	14.04	5143	22069.58	1.78#(1.15, 2.63)	4.97(-0.58, 10.52)
Female	Undifferen	Septicemia	Kidney and Renal Pelvis	3	2.31	1542	6169.62	1.3(0.27, 3.8)	1.12(-6.2, 8.44)
Female	Undifferen	Septicemia	Thyroid	0	0.41	680	1416.98	0(0, 9.09)	-2.86(-11.72, 6)
Female	Undifferen	Septicemia	Lymphoma	5	0.85	572	3869.51	5.86#(1.9, 13.68)	10.72(-1.52, 22.96)
Female	Undifferen	Septicemia	Leukemia	0	0	60	121.81	0(0, 1140.42)	-0.27(-0.27, -0.27)
Female	Unknown	Septicemia	All Sites	2065	1356.57	572977	4228520.59	1.52#(1.46, 1.59)	1.68(1.41, 1.95)
Female	Unknown	Septicemia	Oral Cavity and Pharynx	20	16.55	5751	49424.84	1.21(0.74, 1.87)	0.7(-1.7, 3.1)
Female	Unknown	Septicemia	Stomach	23	8.82	6347	19439.25	2.61#(1.65, 3.91)	7.29(1.61, 12.97)
Female	Unknown	Septicemia	Colon and Rectum	152	100.89	31541	187455.12	1.51#(1.28, 1.77)	2.73(1.07, 4.39)
Female	Unknown	Septicemia	Liver	27	2.92	6791	11846.28	9.24#(6.09, 13.45)	20.33(11.29, 29.37)
Female	Unknown	Septicemia	Pancreas	61	9.94	26918	22159.31	6.14#(4.69, 7.88)	23.04(15.6, 30.48)
Female	Unknown	Septicemia	Lung and Bronchus	164	56.58	80782	135812.21	2.90#(2.47, 3.38)	7.91(5.77, 10.05)
Female	Unknown	Septicemia	Skin excluding Basal and Squamous	127	157.97	52300	662943.81	0.80#(0.67, 0.96)	-0.47(-0.97, 0.03)
Female	Unknown	Septicemia	Breast	575	540.03	104829	1275099.96	1.06(0.98, 1.16)	0.27(-0.24, 0.78)
Female	Unknown	Septicemia	Cervix Uteri	57	29.28	12267	183715.17	1.95#(1.47, 2.52)	1.51(0.52, 2.5)
Female	Unknown	Septicemia	Corpus Uteri	71	55.49	17745	146216.04	1.28(1, 1.61)	1.06(-0.45, 2.57)
Female	Unknown	Septicemia	Ovary	52	27.58	20499	108711.37	1.89#(1.41, 2.47)	2.25(0.64, 3.86)
Female	Unknown	Septicemia	Urinary Bladder	27	13.48	3695	24067.21	2.00#(1.32, 2.92)	5.62(0.44, 10.8)
Female	Unknown	Septicemia	Kidney and Renal Pelvis	64	29.56	12181	82675.55	2.16#(1.67, 2.76)	4.17(1.88, 6.46)
Female	Unknown	Septicemia	Thyroid	44	67.16	38353	507813.92	0.66#(0.48, 0.88)	-0.46(-0.87, -0.05)
Female	Unknown	Septicemia	Lymphoma	85	55.52	23868	233422.44	1.53#(1.22, 1.89)	1.26(0.26, 2.26)
Female	Unknown	Septicemia	Leukemia	95	46.65	27594	150814.65	2.04#(1.65, 2.49)	3.21(1.66, 4.76)

Table S22

Septicemia risk by tumor stage and site in male cancer patients

Sex	SEER historic stage A (1973-2015)-fpc	Selected Events	Site recode ICD-O-3/WHO 2008	Observed	Expected	Persons	Person Years at Risk	SMR(95%CI)	AER(95%CI)
Male	Localized	Septicemia	All Sites	1512	1071.1	259155	2565969	1.41#(1.34, 1.48)	1.72(1.33, 2.11)
Male	Localized	Septicemia	Oral Cavity and Pharynx	68	49.76	12208	124258.35	1.37#(1.06, 1.73)	1.47(-0.24, 3.18)
Male	Localized	Septicemia	Stomach	47	19.26	6174	36127.48	2.44#(1.79, 3.24)	7.68(3.27, 12.09)
Male	Localized	Septicemia	Colon and Rectum	411	285.81	50133	492616.17	1.44#(1.3, 1.58)	2.54(1.49, 3.59)
Male	Localized	Septicemia	Liver	53	7.06	9013	28087.23	7.51#(5.62, 9.82)	16.36(10.96, 21.76)
Male	Localized	Septicemia	Pancreas	8	2.74	3074	6573.28	2.92#(1.26, 5.76)	8.01(-1.76, 17.78)
Male	Localized	Septicemia	Lung and Bronchus	116	44.13	18568	82423.33	2.63#(2.17, 3.15)	8.72(5.71, 11.73)
Male	Localized	Septicemia	Skin excluding Basal and Squamous	134	163.34	41286	510164.41	0.82#(0.69, 0.97)	-0.58(-1.24, 0.08)
Male	Localized	Septicemia	Prostate	0	0	0	0	0(0, 0)	#DIV/0!
Male	Localized	Septicemia	Urinary Bladder	375	305.99	51356	508610.77	1.23#(1.1, 1.36)	1.36(0.35, 2.37)
Male	Localized	Septicemia	Kidney and Renal Pelvis	107	68.95	19409	193560.3	1.55#(1.27, 1.88)	1.97(0.63, 3.31)
Male	Localized	Septicemia	Thyroid	18	16.86	6300	88701.83	1.07(0.63, 1.69)	0.13(-1.17, 1.43)
Male	Localized	Septicemia	Lymphoma	0	0	0	0	0(0, 0)	#DIV/0!
Male	Localized	Septicemia	Leukemia	0	0	0	0	0(0, 0)	#DIV/0!
Male	Regional	Septicemia	All Sites	919	413.76	197255	1063299.95	2.22#(2.08, 2.37)	4.75(4.08, 5.42)
Male	Regional	Septicemia	Oral Cavity and Pharynx	98	34.3	20587	123023.01	2.86#(2.32, 3.48)	5.18(3.35, 7.01)
Male	Regional	Septicemia	Stomach	36	12.61	10692	29682.48	2.86#(2, 3.95)	7.88(3.28, 12.48)
Male	Regional	Septicemia	Colon and Rectum	293	192.63	50924	359919.91	1.52#(1.35, 1.71)	2.79(1.59, 3.99)
Male	Regional	Septicemia	Liver	29	2.18	6851	9415.12	13.28#(8.89, 19.07)	28.48(16.87, 40.09)
Male	Regional	Septicemia	Pancreas	41	4.93	9528	14212.81	8.32#(5.97, 11.29)	25.38(16.05, 34.71)
Male	Regional	Septicemia	Lung and Bronchus	139	35.29	35823	83761.98	3.94#(3.31, 4.65)	12.38(9.29, 15.47)
Male	Regional	Septicemia	Skin excluding Basal and Squamous	25	16.65	6872	55201.42	1.5(0.97, 2.22)	1.51(-0.78, 3.8)
Male	Regional	Septicemia	Prostate	0	0	0	0	0(0, 0)	#DIV/0!
Male	Regional	Septicemia	Urinary Bladder	102	38.77	13539	66079.51	2.63#(2.15, 3.19)	9.57(6.05, 13.09)
Male	Regional	Septicemia	Kidney and Renal Pelvis	40	20.6	8128	55256.9	1.94#(1.39, 2.64)	3.51(0.75, 6.27)
Male	Regional	Septicemia	Thyroid	10	11.19	5268	69214.88	0.89(0.43, 1.64)	-0.17(-1.47, 1.13)
Male	Regional	Septicemia	Lymphoma	0	0	0	0	0(0, 0)	#DIV/0!
Male	Regional	Septicemia	Leukemia	0	0	0	0	0(0, 0)	#DIV/0!
Male	Distant	Septicemia	All Sites	729	222.35	277432	659562.51	3.28#(3.04, 3.53)	7.68(6.76, 8.6)
Male	Distant	Septicemia	Oral Cavity and Pharynx	19	4.95	5791	18851.76	3.84#(2.31, 5.99)	7.45(2.36, 12.54)
Male	Distant	Septicemia	Stomach	18	3.33	12959	10603.94	5.40#(3.2, 8.53)	13.83(5.3, 22.36)
Male	Distant	Septicemia	Colon and Rectum	79	19.43	33074	55164.2	4.07#(3.22, 5.07)	10.8(7.28, 14.32)
Male	Distant	Septicemia	Liver	8	0.6	4641	2628.85	13.33#(5.75, 26.26)	28.15(6.32, 49.98)
Male	Distant	Septicemia	Pancreas	28	3.2	20620	11048.7	8.75#(5.81, 12.64)	22.44(12.54, 32.34)
Male	Distant	Septicemia	Lung and Bronchus	133	21.91	80438	58991.34	6.07#(5.08, 7.19)	18.83(14.7, 22.96)
Male	Distant	Septicemia	Skin excluding Basal and Squamous	3	2.09	2947	6605.28	1.43(0.3, 4.19)	1.38(-5.31, 8.07)
Male	Distant	Septicemia	Prostate	68	34.75	14879	47963.68	1.96#(1.52, 2.48)	6.93(2.79, 11.07)
Male	Distant	Septicemia	Urinary Bladder	11	1.55	2858	3403.3	7.12#(3.55, 12.73)	27.78(7.41, 48.15)
Male	Distant	Septicemia	Kidney and Renal Pelvis	15	4.45	9732	16280.93	3.37#(1.89, 5.56)	6.48(1.17, 11.79)
Male	Distant	Septicemia	Thyroid	1	1.41	1054	7426.08	0.71(0.02, 3.94)	-0.56(-4.66, 3.54)
Male	Distant	Septicemia	Lymphoma	0	0	0	0	0(0, 0)	#DIV/0!
Male	Distant	Septicemia	Leukemia	183	82.66	46314	277843.49	2.21#(1.9, 2.56)	3.61(2.46, 4.76)
Male	Localized/regional (Prostate cases)	Septicemia	All Sites	1004	1426.31	227922	2405141.42	0.70#(0.66, 0.75)	-1.76(-2.16, -1.36)
Male	Localized/regional (Prostate cases)	Septicemia	Oral Cavity and Pharynx	0	0	0	0	0(0, 0)	#DIV/0!
Male	Localized/regional (Prostate cases)	Septicemia	Stomach	0	0	0	0	0(0, 0)	#DIV/0!
Male	Localized/regional (Prostate cases)	Septicemia	Colon and Rectum	0	0	0	0	0(0, 0)	#DIV/0!
Male	Localized/regional (Prostate cases)	Septicemia	Liver	0	0	0	0	0(0, 0)	#DIV/0!
Male	Localized/regional (Prostate cases)	Septicemia	Pancreas	0	0	0	0	0(0, 0)	#DIV/0!
Male	Localized/regional (Prostate cases)	Septicemia	Lung and Bronchus	0	0	0	0	0(0, 0)	#DIV/0!
Male	Localized/regional (Prostate cases)	Septicemia	Skin excluding Basal and Squamous	0	0	0	0	0(0, 0)	#DIV/0!
Male	Localized/regional (Prostate cases)	Septicemia	Prostate	1004	1426.31	227922	2405141.42	0.70#(0.66, 0.75)	-1.76(-2.16, -1.36)
Male	Localized/regional (Prostate cases)	Septicemia	Urinary Bladder	0	0	0	0	0(0, 0)	#DIV/0!
Male	Localized/regional (Prostate cases)	Septicemia	Kidney and Renal Pelvis	0	0	0	0	0(0, 0)	#DIV/0!
Male	Localized/regional (Prostate cases)	Septicemia	Thyroid	0	0	0	0	0(0, 0)	#DIV/0!
Male	Localized/regional (Prostate cases)	Septicemia	Lymphoma	0	0	0	0	0(0, 0)	#DIV/0!
Male	Localized/regional (Prostate cases)	Septicemia	Leukemia	0	0	0	0	0(0, 0)	#DIV/0!
Male	Unstaged	Septicemia	All Sites	855	324.18	207213	1045073.58	2.64#(2.46, 2.82)	5.08(4.44, 5.72)
Male	Unstaged	Septicemia	Oral Cavity and Pharynx	11	5.8	2511	15097.67	1.9(0.95, 3.39)	3.44(-1.88, 8.76)
Male	Unstaged	Septicemia	Stomach	11	3	3350	5114.23	3.66#(1.83, 6.56)	15.64(1.31, 29.97)
Male	Unstaged	Septicemia	Colon and Rectum	37	16.49	7176	28858.43	2.24#(1.58, 3.09)	7.11(2.15, 12.07)
Male	Unstaged	Septicemia	Liver	13	1.04	4025	3322.26	12.45#(6.63, 21.3)	35.99(13.92, 58.06)
Male	Unstaged	Septicemia	Pancreas	11	2.11	4489	3856.96	5.21#(2.6, 9.32)	23.04(4.66, 41.42)
Male	Unstaged	Septicemia	Lung and Bronchus	25	7.72	10553	13280.89	3.24#(2.1, 4.78)	13.01(4.57, 21.45)
Male	Unstaged	Septicemia	Skin excluding Basal and Squamous	9	8.86	2559	27061.86	1.02(0.46, 1.93)	0.05(-3.01, 3.11)
Male	Unstaged	Septicemia	Prostate	47	49.29	8098	57990.39	0.95(0.7, 1.27)	-0.39(-3.71, 2.93)
Male	Unstaged	Septicemia	Urinary Bladder	22	10.68	2762	18671.19	2.06#(1.29, 3.12)	6.07(0.07, 12.07)
Male	Unstaged	Septicemia	Kidney and Renal Pelvis	10	3.43	1700	7023.68	2.92#(1.4, 5.37)	9.36(-0.86, 19.58)
Male	Unstaged	Septicemia	Thyroid	2	0.74	314	3684.18	2.7(0.33, 9.77)	3.42(-5.38, 12.22)
Male	Unstaged	Septicemia	Lymphoma	300	138.74	69052	537757.73	2.16#(1.92, 2.42)	3(2.24, 3.76)
Male	Unstaged	Septicemia	Leukemia	0	0	0	0	0(0, 0)	#DIV/0!

Table S23

Septicemia risk by tumor stage and site in female cancer patients

Sex	SEER historic stage A (1973-2015)-fpc	Selected Events	Site recode ICD-O-3/WHO 2008	Observed	Expected	Persons	Person Years at Risk	SMR(95%CI)	AER(95%CI)
Female	Localized	Septicemia	All Sites	2397	2455.76	511503	6308978.82	0.98(0.94, 1.02)	-0.09(-0.31, 0.13)
Female	Localized	Septicemia	Oral Cavity and Pharynx	29	29.45	7417	82132.51	0.98(0.66, 1.41)	-0.06(-1.88, 1.76)
Female	Localized	Septicemia	Stomach	30	17.25	4656	32511.19	1.74#(1.17, 2.48)	3.92(-0.22, 8.06)
Female	Localized	Septicemia	Colon and Rectum	371	334.44	50423	529869.6	1.11(1, 1.23)	0.69(-0.29, 1.67)
Female	Localized	Septicemia	Liver	15	2.82	3365	11084.71	5.32#(2.98, 8.78)	10.99(3.53, 18.45)
Female	Localized	Septicemia	Pancreas	11	3.46	3560	8938.94	3.18#(1.59, 5.69)	8.44(0.11, 16.77)
Female	Localized	Septicemia	Lung and Bronchus	107	50.67	18324	105330.16	2.11#(1.73, 2.55)	5.35(3.01, 7.69)
Female	Localized	Septicemia	Skin excluding Basal and Squamous	102	135.55	40043	577784.73	0.75#(0.61, 0.91)	-0.58(-1.1, -0.06)
Female	Localized	Septicemia	Breast	896	1150.12	218046	2760290.93	0.78#(0.73, 0.83)	-0.92(-1.24, -0.6)
Female	Localized	Septicemia	Cervix Uteri	62	39.88	15436	271350.13	1.55#(1.19, 1.99)	0.82(0.09, 1.55)
Female	Localized	Septicemia	Corpus Uteri	382	367.28	61049	850241.94	1.04(0.94, 1.15)	0.17(-0.46, 0.8)
Female	Localized	Septicemia	Ovary	26	32.53	8964	135885.48	0.8(0.52, 1.17)	-0.48(-1.58, 0.62)
Female	Localized	Septicemia	Urinary Bladder	127	109.72	17289	185482.66	1.16(0.96, 1.38)	0.93(-0.7, 2.56)
Female	Localized	Septicemia	Kidney and Renal Pelvis	90	50.78	13216	142705.11	1.77#(1.43, 2.18)	2.75(1.12, 4.38)
Female	Localized	Septicemia	Thyroid	34	55.39	26761	392547.61	0.61#(0.43, 0.86)	-0.54(-1.01, -0.07)
Female	Localized	Septicemia	Lymphoma	0	0	0	0	0(0, 0)	#DIV/0!
Female	Localized	Septicemia	Leukemia	0	0	0	0	0(0, 0)	#DIV/0!
Female	Regional	Septicemia	All Sites	1327	863.17	298131	2426543.24	1.54#(1.46, 1.62)	1.91(1.53, 2.29)
Female	Regional	Septicemia	Oral Cavity and Pharynx	44	16.27	8142	50452.32	2.70#(1.96, 3.63)	5.5(2.49, 8.51)
Female	Regional	Septicemia	Stomach	26	9.12	5724	18998.32	2.85#(1.86, 4.18)	8.89(2.78, 15)
Female	Regional	Septicemia	Colon and Rectum	313	245.43	53302	408170.68	1.28#(1.14, 1.42)	1.66(0.53, 2.79)
Female	Regional	Septicemia	Liver	6	0.77	2202	3603.2	7.77#(2.85, 16.91)	14.51(0.37, 28.65)
Female	Regional	Septicemia	Pancreas	31	5.16	9725	14411.52	6.01#(4.08, 8.52)	17.93(9.76, 26.1)
Female	Regional	Septicemia	Lung and Bronchus	76	30.53	26700	78375.73	2.49#(1.96, 3.12)	5.8(3.22, 8.38)
Female	Regional	Septicemia	Skin excluding Basal and Squamous	17	14.02	4749	47545.4	1.21(0.71, 1.94)	0.63(-1.67, 2.93)
Female	Regional	Septicemia	Breast	499	390.24	119141	1236788.95	1.28#(1.17, 1.4)	0.88(0.41, 1.35)
Female	Regional	Septicemia	Cervix Uteri	61	17.95	9902	79759.31	3.40#(2.6, 4.37)	5.4(3.22, 7.58)
Female	Regional	Septicemia	Corpus Uteri	76	41.53	12438	104176.01	1.83#(1.44, 2.29)	3.31(1.27, 5.35)
Female	Regional	Septicemia	Ovary	14	7.5	3281	27174.52	1.87#(1.02, 3.13)	2.39(-0.95, 5.73)
Female	Regional	Septicemia	Urinary Bladder	49	15.95	5939	26218.41	3.07#(2.27, 4.06)	12.6(6.58, 18.62)
Female	Regional	Septicemia	Kidney and Renal Pelvis	11	12.11	4511	31744.92	0.91(0.45, 1.63)	-0.35(-3.32, 2.62)
Female	Regional	Septicemia	Thyroid	17	23.19	13797	195923.33	0.73(0.43, 1.17)	-0.32(-0.95, 0.31)
Female	Regional	Septicemia	Lymphoma	0	0	0	0	0(0, 0)	#DIV/0!
Female	Regional	Septicemia	Leukemia	0	0	0	0	0(0, 0)	#DIV/0!
Female	Distant	Septicemia	All Sites	755	230.77	272152	743091.13	3.27#(3.04, 3.51)	7.05(6.22, 7.88)
Female	Distant	Septicemia	Oral Cavity and Pharynx	17	1.99	2048	7199.45	8.53#(4.97, 13.66)	20.85(9, 32.7)
Female	Distant	Septicemia	Stomach	17	1.97	7196	6156.03	8.62#(5.02, 13.81)	24.41(10.56, 38.26)
Female	Distant	Septicemia	Colon and Rectum	97	20.46	31426	52917.89	4.74#(3.84, 5.78)	14.46(10.45, 18.47)
Female	Distant	Septicemia	Liver	5	0.22	1589	1332.76	22.94#(7.45, 53.54)	35.88(2.34, 69.42)
Female	Distant	Septicemia	Pancreas	34	3.34	18486	10274.73	10.17#(7.04, 14.21)	29.84(18.2, 41.48)
Female	Distant	Septicemia	Lung and Bronchus	96	19.24	61835	56384.52	4.99#(4.04, 6.09)	13.61(9.88, 17.34)
Female	Distant	Septicemia	Skin excluding Basal and Squamous	3	1.35	1480	4049.17	2.22(0.46, 6.49)	4.07(-6.02, 14.16)
Female	Distant	Septicemia	Breast	82	25.59	26364	90035.01	3.20#(2.55, 3.98)	6.27(4.01, 8.53)
Female	Distant	Septicemia	Cervix Uteri	10	1.29	3058	7405.71	7.74#(3.71, 14.24)	11.76(2.87, 20.65)
Female	Distant	Septicemia	Corpus Uteri	42	10.19	7883	28824.38	4.12#(2.97, 5.57)	11.03(6.12, 15.94)
Female	Distant	Septicemia	Ovary	84	34.18	33252	127501.04	2.46#(1.96, 3.04)	3.91(2.24, 5.58)
Female	Distant	Septicemia	Urinary Bladder	7	0.69	1477	1454.55	10.10#(4.06, 20.8)	43.36(6.08, 80.64)
Female	Distant	Septicemia	Kidney and Renal Pelvis	8	2.27	5267	9467.52	3.52#(1.52, 6.94)	6.05(-0.58, 12.68)
Female	Distant	Septicemia	Thyroid	5	1.74	1570	13571.56	2.88(0.94, 6.72)	2.4(-1.35, 6.15)
Female	Distant	Septicemia	Lymphoma	0	0	0	0	0(0, 0)	#DIV/0!
Female	Distant	Septicemia	Leukemia	131	68.22	35116	216863.89	1.92#(1.61, 2.28)	2.89(1.61, 4.17)
Female	Localized/regional (Prostate cases)	Septicemia	All Sites	0	0	0	0	0(0, 0)	#DIV/0!
Female	Localized/regional (Prostate cases)	Septicemia	Oral Cavity and Pharynx	0	0	0	0	0(0, 0)	#DIV/0!
Female	Localized/regional (Prostate cases)	Septicemia	Stomach	0	0	0	0	0(0, 0)	#DIV/0!
Female	Localized/regional (Prostate cases)	Septicemia	Colon and Rectum	0	0	0	0	0(0, 0)	#DIV/0!
Female	Localized/regional (Prostate cases)	Septicemia	Liver	0	0	0	0	0(0, 0)	#DIV/0!
Female	Localized/regional (Prostate cases)	Septicemia	Pancreas	0	0	0	0	0(0, 0)	#DIV/0!
Female	Localized/regional (Prostate cases)	Septicemia	Lung and Bronchus	0	0	0	0	0(0, 0)	#DIV/0!
Female	Localized/regional (Prostate cases)	Septicemia	Skin excluding Basal and Squamous	0	0	0	0	0(0, 0)	#DIV/0!
Female	Localized/regional (Prostate cases)	Septicemia	Breast	0	0	0	0	0(0, 0)	#DIV/0!
Female	Localized/regional (Prostate cases)	Septicemia	Cervix Uteri	0	0	0	0	0(0, 0)	#DIV/0!
Female	Localized/regional (Prostate cases)	Septicemia	Corpus Uteri	0	0	0	0	0(0, 0)	#DIV/0!
Female	Localized/regional (Prostate cases)	Septicemia	Ovary	0	0	0	0	0(0, 0)	#DIV/0!
Female	Localized/regional (Prostate cases)	Septicemia	Urinary Bladder	0	0	0	0	0(0, 0)	#DIV/0!
Female	Localized/regional (Prostate cases)	Septicemia	Kidney and Renal Pelvis	0	0	0	0	0(0, 0)	#DIV/0!
Female	Localized/regional (Prostate cases)	Septicemia	Thyroid	0	0	0	0	0(0, 0)	#DIV/0!
Female	Localized/regional (Prostate cases)	Septicemia	Lymphoma	0	0	0	0	0(0, 0)	#DIV/0!
Female	Localized/regional (Prostate cases)	Septicemia	Leukemia	0	0	0	0	0(0, 0)	#DIV/0!
Female	Unstaged	Septicemia	All Sites	728	328.69	184122	982856.82	2.21#(2.06, 2.38)	4.06(3.41, 4.71)
Female	Unstaged	Septicemia	Oral Cavity and Pharynx	5	3.41	1372	8808.83	1.47(0.48, 3.42)	1.8(-4.65, 8.25)
Female	Unstaged	Septicemia	Stomach	12	3.1	2942	4980.26	3.87#(2, 6.76)	17.87(2.59, 33.15)
Female	Unstaged	Septicemia	Colon and Rectum	45	19.82	8900	30019.66	2.27#(1.66, 3.04)	8.39(3.14, 13.64)
Female	Unstaged	Septicemia	Liver	10	0.7	1819	1877.75	14.20#(6.81, 26.11)	49.5(15.44, 83.56)
Female	Unstaged	Septicemia	Pancreas	9	2.97	5911	4688.36	3.03#(1.39, 5.75)	12.86(-1.59, 27.31)
Female	Unstaged	Septicemia	Lung and Bronchus	20	7.74	8848	13271.45	2.59#(1.58, 3.99)	9.24(1.47, 17.01)
Female	Unstaged	Septicemia	Skin excluding Basal and Squamous	8	7.99	2345	31249.96	1(0.43, 1.97)	0(-2.51, 2.51)
Female	Unstaged	Septicemia	Breast	51	31.99	9827	70288.4	1.59#(1.19, 2.1)	2.7(0.16, 5.24)
Female	Unstaged	Septicemia	Cervix Uteri	5	3.18	1634	17312.61	1.57(0.51, 3.67)	1.05(-2.19, 4.29)
Female	Unstaged	Septicemia	Corpus Uteri	21	11.3	2922	24112.12	1.86#(1.15, 2.84)	4.02(-0.6, 8.64)
Female	Unstaged	Septicemia	Ovary	4	3.62	2877	11177.39	1.1(0.3, 2.83)	0.34(-4.5, 5.18)
Female	Unstaged	Septicemia	Urinary Bladder	10	4.35	1332	7073.92	2.30#(1.1, 4.23)	7.98(-2.51, 18.47)
Female	Unstaged	Septicemia	Kidney and Renal Pelvis	10	2.93	1373	5245.6	3.42#(1.64, 6.28)	13.48(0.06, 26.9)
Female	Unstaged	Septicemia	Thyroid	1	2.02	987	13635.59	0.5(0.01, 2.76)	-0.75(-3.25, 1.75)
Female	Unstaged	Septicemia	Lymphoma	226	148.81	57499	480699.74	1.52#(1.33, 1.73)	1.61(0.82, 2.4)
Female	Unstaged	Septicemia	Leukemia	0	0	0	0	0(0, 0)	#DIV/0!

Table S24

Odds ratios and hazard ratios of Sepsis among male cancer patients.

	Logistic regression model				Cox proportional hazards model			
**Variables**	**OR**	**P value**	**95% CI**		**HR**	**P value**	**95% CI**	
Age at Diagnosis								
0-19	1				1			
20-29	1.287008	0.357	0.7523349	2.201664	2.151811	0.007	1.232316	3.757389
30-39	1.57581	0.064	0.9742861	2.548714	3.807934	0	2.296612	6.313805
40-49	2.433578	0	1.551247	3.817769	8.525398	0	5.297944	13.71899
50-59	3.394873	0	2.187245	5.26926	16.53636	0	10.3794	26.34555
60-69	4.797223	0	3.09694	7.430997	29.98493	0	18.83967	47.72357
70-79	6.230258	0	4.02109	9.653133	55.0756	0	34.56673	87.75264
80+	6.682541	0	4.303437	10.3769	98.22515	0	61.44915	157.0108
Race								
White	1				1			
Black	1.84986	0	1.705037	2.006985	2.21649	0	2.036838	2.411988
Other (American Indian/AK Native, Asian/Pacific Islander)	1.051756	0.312	0.9537815	1.159795	1.059703	0.267	0.9565957	1.173923
Unknown	0.4113638	0.001	0.2471153	0.6847822	0.4164309	0.001	0.2459955	0.7049508
Year of diagnosis								
1975-1979	1				1			
1980-1989	1.474612	0	1.310336	1.659485	1.263777	0	1.118674	1.427702
1990-1999	1.102778	0.891	0.2734885	4.44669	0.8367646	0.802	0.2080499	3.36542
2000-2009	1.002871	0.997	0.248721	4.043685	0.8936561	0.874	0.2221381	3.595157
2010-2019	0.5141956	0.35	0.1273821	2.075622	0.8909643	0.871	0.2211598	3.589338
Grade								
Grade I	1				1			
Grade II	0.8336741	0	0.7632185	0.9106338	0.9333018	0.133	0.8529523	1.02122
Grade III	0.6799841	0	0.617403	0.7489085	1.059857	0.253	0.9593474	1.170897
Grade IV	0.5770462	0	0.4930255	0.6753856	1.152267	0.099	0.9734866	1.363881
Unknown	0.7420877	0	0.6791142	0.8109008	1.036746	0.442	0.9456009	1.136678
Stage								
Localized	1				1			
Regional	0.7965623	0	0.7313674	0.8675687	1.268713	0	1.160753	1.386714
Distant	0.7253873	0	0.661645	0.7952704	1.081155	0.165	0.9684599	1.206963
Unstaged	0.4653817	0	0.421631	0.5136723	0.8720208	0	0.8083238	0.9407371
Localized/regional(Prostatecases)	0.6973427	0	0.6483365	0.7500533	0.4725094	0	0.4282813	0.5213049
Surgery								
No	1				1			
Yes	1.077136	0.017	1.013358	1.144928	0.7016963	0	0.6548077	0.7519425
Radiation recode								
No	1				1			
Yes	0.8673384	0	0.8152376	0.922769	0.9103465	0.004	0.8533014	0.9712053
Chemotherapy recode								
No	1				1			
Yes	0.9984739	0.967	0.9293842	1.0727	1.294399	0	1.199873	1.396372
Marital status at diagnosis								
Single	1				1			
Married	0.9004537	0.002	0.8422204	0.9627134	0.7079029	0	0.659606	0.759736
Unmarried or Domestic Partner	1.058791	0.241	0.9623014	1.164956	1.02098	0.688	0.9227397	1.129679
Divorced Separated Widowed	0.9832709	0.765	0.8803728	1.098196	0.7272641	0	0.6485229	0.8155658
Unknown	0.7910855	0.641	0.2955559	2.117421	0.9904651	0.985	0.3702976	2.649278
Median household income								
< $35,000	1				1			
$35,000 - $39,999	0.6901601	0.227	0.3781235	1.259697	0.803416	0.506	0.4215037	1.531368
$40,000 - $44,999	0.8457472	0.508	0.5149137	1.389142	0.8489277	0.557	0.4916097	1.465956
$45,000 - $49,999	0.8997701	0.659	0.5632278	1.437405	0.8729848	0.608	0.5197405	1.466313
$50,000 - $54,999	0.8309194	0.428	0.5255382	1.313752	0.8230089	0.452	0.4952378	1.367714
$55,000 - $59,999	0.800494	0.342	0.5060364	1.266294	0.7398302	0.246	0.4446127	1.231068
$60,000 - $64,999	0.9528229	0.836	0.6025562	1.5067	0.912227	0.723	0.5485767	1.51694
$65,000 - $69,999	0.9526247	0.835	0.6025246	1.506153	0.9276676	0.772	0.55782	1.542733
$70,000 - $74,999	1.181811	0.475	0.7474335	1.868632	1.136154	0.623	0.6831052	1.889673
$75,000+	0.8914317	0.621	0.56555	1.405093	0.8373642	0.491	0.5050313	1.388387
Rural – Urban Continuum Code								
Counties in metropolitan areas ge 1 million pop	1				1			
Counties in metropolitan areas of 250,000 to 1 million pop	1.3842	0	1.290237	1.485005	1.390296	0	1.292178	1.495864
Counties in metropolitan areas of lt 250 thousand pop	0.8722254	0.051	0.760307	1.000618	0.8623439	0.044	0.7467084	0.9958867
Nonmetropolitan counties adjacent to a metropolitan area	1.209327	0.004	1.062252	1.376766	1.197916	0.01	1.044559	1.373787
Nonmetropolitan counties not adjacent to a metropolitan a	1.026589	0.74	0.879364	1.198464	1.056703	0.508	0.8975372	1.244095

Table S25

Odds ratios and hazard ratios of Sepsis among female cancer patients.								
	Logistic regression model				Cox proportional hazards model			
Variables	OR	P value	95% CI		HR	P value	95% CI	
Age at Diagnosis								
0-19	1				1			
20-29	0.8988883	0.677	0.5439532	1.485422	1.658464	0.059	0.9805899	2.804948
30-39	0.9478891	0.812	0.6097785	1.473476	2.437704	0	1.52873	3.88715
40-49	1.544877	0.039	1.022674	2.33373	4.935409	0	3.178634	7.663122
50-59	2.476806	0	1.651519	3.714499	9.937949	0	6.439238	15.33766
60-69	3.470712	0	2.31842	5.195712	19.16965	0	12.42883	29.56637
70-79	3.990531	0	2.665054	5.975241	32.71429	0	21.18202	50.52515
80+	3.874621	0	2.582101	5.814135	51.81905	0	33.43661	80.3076
Race								
White	1				1			
Black	2.066116	0	1.89285	2.255242	2.318246	0	2.115023	2.540995
Other (American Indian/AK Native, Asian/Pacific Islander)	1.113208	0.043	1.003469	1.234949	1.090898	0.114	0.9793924	1.215099
Unknown	0.1839973	0.001	0.0689055	0.4913249	0.1470415	0.001	0.0473542	0.4565843
Year of diagnosis								
1975-1979	1				1			
1980-1989	1.296671	0	1.157496	1.45258	1.403753	0	1.247276	1.579861
1990-1999	3.085433	0.007	1.364909	6.974745	3.67222	0.002	1.631577	8.265131
2000-2009	2.633964	0.02	1.166	5.950054	3.92069	0.001	1.741873	8.824874
2010-2019	1.426939	0.394	0.6298985	3.232511	3.843014	0.001	1.701176	8.6815
Grade								
Grade I	1				1			
Grade II	0.9869482	0.783	0.8990391	1.083453	1.071472	0.151	0.9750584	1.177418
Grade III	0.8445184	0.001	0.7619662	0.9360143	1.132889	0.019	1.020723	1.257381
Grade IV	0.7880253	0.006	0.6657086	0.9328165	1.312427	0.002	1.101706	1.563452
Unknown	0.8946006	0.018	0.8159743	0.9808033	0.9321885	0.142	0.8487117	1.023876
Other								
Stage								
Localized	1				1			
Regional	0.9382532	0.077	0.874218	1.006979	1.388827	0	1.292519	1.492311
Distant	0.5562385	0	0.504728	0.6130059	1.669591	0	1.496822	1.862301
Unstaged	0.635833	0	0.5812967	0.6954858	1.30307	0	1.180607	1.438236
Surgery								
No	1				1			
Yes	1.031064	0.431	0.955484	1.112622	0.5644017	0	0.5170916	0.6160402
Radiation recode								
No	1				1			
Yes	0.8547594	0	0.8022745	0.9106778	0.9380333	0.051	0.8796332	1.000311
Chemotherapy recode								
No	1				1			
Yes	0.919327	0.021	0.8560727	0.9872551	0.963066	0.319	0.8944175	1.036983
Marital status at diagnosis								
Single	1				1			
Married	0.905579	0.002	0.8498903	0.9649167	0.7389551	0	0.6921221	0.7889571
Unmarried or Domestic Partner	1.145833	0.003	1.04611	1.255064	1.139705	0.007	1.036926	1.252671
Divorced Separated Widowed	1.092662	0.156	0.9668262	1.234875	0.8676855	0.029	0.7636656	0.9858741
Unknown	0.5611311	0.415	0.1398819	2.250957	0.8541991	0.824	0.2130705	3.424482
Median household income								
< $35,000	1				1			
$35,000 - $39,999	0.8245523	0.593	0.406255	1.673546	0.8773308	0.723	0.425383	1.80945
$40,000 - $44,999	0.9984063	0.996	0.5476961	1.820015	0.854003	0.62	0.4576578	1.593595
$45,000 - $49,999	0.9372025	0.825	0.5268332	1.667223	0.7721256	0.398	0.4240797	1.405816
$50,000 - $54,999	0.8971301	0.706	0.5105064	1.576557	0.7233965	0.279	0.4025566	1.299948
$55,000 - $59,999	0.8656301	0.616	0.4922481	1.522232	0.6940039	0.222	0.3859832	1.24783
$60,000 - $64,999	1.062891	0.832	0.6048326	1.867852	0.8652131	0.628	0.4815209	1.554644
$65,000 - $69,999	1.018402	0.949	0.5793277	1.790253	0.8557454	0.603	0.4761261	1.538038
$70,000 - $74,999	1.327308	0.325	0.7553093	2.332484	1.106863	0.734	0.6160068	1.988852
$75,000+	1.006326	0.982	0.5744552	1.762874	0.8095662	0.477	0.452019	1.449934
Rural – Urban Continuum Code								
Counties in metropolitan areas ge 1 million pop	1				1			
Counties in metropolitan areas of 250,000 to 1 million pop	1.363548	0	1.261934	1.473344	1.390223	0	1.282319	1.507207
Counties in metropolitan areas of lt 250 thousand pop	0.8653794	0.067	0.7411387	1.010447	0.8951722	0.175	0.7627482	1.050587
Nonmetropolitan counties adjacent to a metropolitan area	1.261495	0.002	1.089984	1.459994	1.25157	0.004	1.073265	1.459497
Nonmetropolitan counties not adjacent to a metropolitan a	1.147553	0.117	0.9662527	1.362872	1.152256	0.127	0.9604913	1.382308
